# Metabolite Profiling and Transcriptome Analysis Provide Insight into Seed Coat Color in *Brassica juncea*

**DOI:** 10.3390/ijms22137215

**Published:** 2021-07-05

**Authors:** Shulin Shen, Yunshan Tang, Chao Zhang, Nengwen Yin, Yuanyi Mao, Fujun Sun, Si Chen, Ran Hu, Xueqin Liu, Guoxia Shang, Liezhao Liu, Kun Lu, Jiana Li, Cunmin Qu

**Affiliations:** 1 Chongqing Rapeseed Engineering Research Center, College of Agronomy and Biotechnology, Southwest University, Chongqing 400715, China; ssl9942@email.swu.edu.cn (S.S.); tys98@email.swu.edu.cn (Y.T.); 18083606406@163.com (C.Z.); nwyin80@126.com (N.Y.); myy19990831@email.swu.edu.cn (Y.M.); drsunfujun@email.swu.edu.cn (F.S.); sichen96@email.swu.edu.cn (S.C.); hr1996@email.swu.edu.cn (R.H.); swuliuxueqin@email.swu.edu.cn (X.L.); liezhao2003@126.com (L.L.); drlukun@swu.edu.cn (K.L.); 2 Academy of Agricultural Sciences, Southwest University, Chongqing 400715, China; 3 Engineering Research Center of South Upland Agriculture, Ministry of Education, Chongqing 400715, China; 4 Academy of Agricultural and Forestry Sciences, Qinghai University, Xining 810016, China; shangguoxia@126.com

**Keywords:** *Brassica juncea* L., transcriptome, flavonoids, *TRANSPARENT TESTA* *8*, metabolic profiling, expression patterns

## Abstract

The allotetraploid species *Brassica juncea* (mustard) is grown worldwide as oilseed and vegetable crops; the yellow seed-color trait is particularly important for oilseed crops. Here, to examine the factors affecting seed coat color, we performed a metabolic and transcriptomic analysis of yellow- and dark-seeded *B*. *juncea* seeds. In this study, we identified 236 compounds, including 31 phenolic acids, 47 flavonoids, 17 glucosinolates, 38 lipids, 69 other hydroxycinnamic acid compounds, and 34 novel unknown compounds. Of these, 36 compounds (especially epicatechin and its derivatives) accumulated significantly different levels during the development of yellow- and dark-seeded *B*. *juncea*. In addition, the transcript levels of *BjuDFR*, *BjuANS*,*BjuBAN*, *BjuTT8,* and *BjuTT19* were closely associated with changes to epicatechin and its derivatives during seed development, implicating this pathway in the seed coat color determinant in *B*. *juncea*. Furthermore, we found numerous variations of sequences in the *TT8A* genes that may be associated with the stability of seed coat color in *B*. *rapa*, *B*. *napus,* and *B*. *juncea*, which might have undergone functional differentiation during polyploidization in the *Brassica* species. The results provide valuable information for understanding the accumulation of metabolites in the seed coat color of *B. juncea* and lay a foundation for exploring the underlying mechanism.

## 1. Introduction

*Brassica juncea* L. (AABB, *n* = 18), also called mustard, is an important crop that was formed by means of the hybridization of the ancestors of the diploid species *Brassica rapa* (AA, *n* = 10) and *Brassica nigra* (BB, *n* = 8) [1]. *B. juncea* has a long history of cultivation and is cultivated worldwide in countries such as China, India, Canada, and Europe. Different varieties and cultivars have been classified for root-use, stem-use, leaf-use and oil-use based on different edible organs [2,3,4]. In *Brassica* oilseed crops, the yellow-seeded varieties are preferred over the dark-seeded varieties due to their thin seed coat and high oil content [5,6]. Among the oilseed *Brassica* species, the naturally yellow-seeded germplasm recourses usually exist in the diploid *B. rapa* (Yellow Sarson type) and allotetraploid *B. juncea* varieties [7,8] but are not found in *Brassica oleracea* and *Brassica napus*. Furthermore, numerous studies have shown that the regulatory mechanisms underlying seed coat color significantly differ among the different species of the *Brassica* genus [7,8,9,10,11,12,13,14,15,16].

In *Brassica* species, the seed pigments are predominantly flavonoid metabolites, i.e., flavonols, anthocyanins, and proanthocyanidins (PAs), which are mainly produced through the pathway of flavonoid biosynthesis [10,17,18]. PAs are the most numerous and widely distributed polyphenols in plants and determine seed coat color in *Arabidopsis thaliana* [9,10,11], *Medicago truncatula* [19,20], sheepgrass (*Leymus chinensis*) [21], and *B*. *napus* [13,16,22]. In addition, advances in metabolomics technologies have led to numerous flavonoids being identified in *B. oleracea* [23], *B. rapa* [24], *B. napus* [14,25], and *B. juncea* [26,27]. However, the pathway of flavonoid biosynthesis has been well studied by the *TT* (*TRANSPARENT TESTA*) loci in model plant *A. thaliana* [18,28,29,30,31,32,33,34], providing valuable resources and references for elucidating the mechanism of seed coat color. Meanwhile, homologs of these *TT* genes have been reported in *B. rapa* [12], *B. napus* [13,15], and *B. juncea* [8,35]. Generally, the flavonoid biosynthesis genes are mainly divided into two categories: structural genes and regulatory genes [18]. Among the structural genes, *chalcone synthase* (*CHS*), *chalcone isomerase* (*CHI*), *flavanone 3-hydroxylase* (*F3H*), and *flavanone 3′-hydroxylase* (*F3′H*) are involved in the production of common precursors (i.e., flavonols and other flavonoid compounds) and have also been called the early biosynthetic genes (EBGs), while the downstream genes of the pathway, including dihydroflavonol reductase (DFR), anthocyanidin synthase (ANS; synonym leucocyanidin dioxygenase, LDOX), and anthocyanidin reductase (ANR/BAN), are usually called the late biosynthetic genes (LBGs) [36,37,38]. Another regulatory genes category mainly includes a series of transcription factor genes (TT1, TT2, TT8, TT16, and TTG1), which are involved in the control of the production of anthocyanin/PAs [18,30]. For example, the transcription factors TT2 (R2R3-MYB), TT8 (basic helix-loop-helix, bHLH), and TTG1 (WD40) [39]. These form the ternary MYB–bHLH–WDR (MBW) complex, which plays a pivotal role in the flavonoid biosynthesis pathway by regulating the expression levels and patterns of the structural genes [39,40,41,42]. Among them, the expression of BjuTT8 is closely associated with seed coat color [8], as seen in A. thaliana [34] and B. rapa [43]. In addition, the R2R3-MYB transcription factors, i.e., PAP1/MYB75, PAP2, MYB113, and MYB114, also positively regulate the accumulation of anthocyanin [44,45]. In addition, some gene encoding transfer proteins, such as transporters glutathione S-transferase 26 (GST26/TT19), auto-inhibited H+-ATPase isoform 10 (AHA10), MATE transporter (TT12), and a laccase gene, *laccase 15* (*LAC15*) have been found to be involved in flavonoid transport [18,29,30,46,47,48]. These results lay a foundation for elucidating the mechanisms of seed coloration in Brassica species. 

Recently, multi-omics analysis (integrating transcriptomics, metabolomics, proteomics, and genomics) has become an effective tool for screening and analyzing the key genes of various metabolic pathways in plants [42,49,50]. Furthermore, the gene to metabolite networks are well understood, especially in the flavonoid biosynthesis pathways of many species, including *A*. *thaliana* [51], red and green kale (*B*. *oleracea*) [52], red mustard greens [27], and purple tumorous stem mustard [42]. However, the metabolome and transcriptome of yellow- and dark *B*. *juncea* seeds remain to be explored, and this will provide important clues for elucidating the molecular mechanism underlying seed coat color formation of *B*. *juncea*.

*Brassica* species are ideal models for studying polyploid genome evolution [53], and *B*. *juncea* has the yellow-seeded trait genus naturally [8]. Therefore, we used the genome sequence of *B*. *juncea* [1,54] to perform comprehensive transcriptome and metabolic profiling of developing seeds in yellow- and dark-seeded *B*. *juncea*. In this study, our results identified differentially abundant metabolic compounds, including phenolic acids, flavonoids, lipid compounds, hydroxycinnamic acids, and novel unknown compounds. Moreover, the expression profiles of some genes were investigated by transcriptomic analysis and RT-qPCR and were highly consistent with the accumulation patterns of differential metabolites during seed development in yellow- and dark-seeded *B*. *juncea*. In addition, the different variants of *TT8A* are associated with the seed coat color among the *Brassica* species, indicating that *TT8* underwent functional differentiation during polyploid genome evolution. Our results provide insight into the function of the key genes involved in flavonoid biosynthesis and help to elucidate the molecular mechanisms of seed coat coloration in *B*. *juncea*.

## 2. Results

### 2.1. Metabolite Profiling of Developing B. juncea Seeds

**Figure 1:**
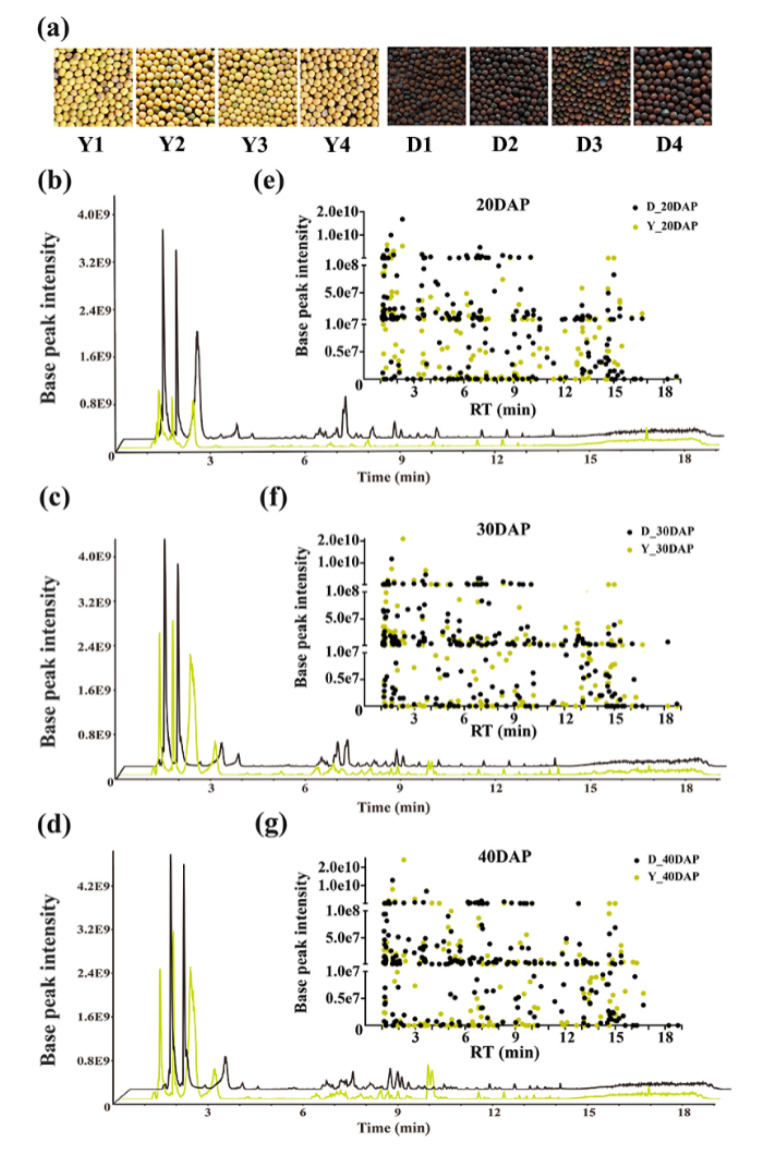
MS data analysis in yellow- and dark-seeded *B*. *juncea*. (**a**) Seed coat color of yellow- and dark-seeded *B*. *juncea*. (**b–d**) Base peak chromatograms of seeds under full scanning (*m*/*z* =100–1500) at 20, 30, and 40 days after pollination (DAP), respectively. The black and green lines indicate dark- and yellow-seeded *B*. *juncea*, respectively. (**e–g**) All of the detected compounds were generated from seeds at 20, 30, and 40 DAP, respectively. The green and black dots represent compounds from yellow- and dark-seeded *B*. *juncea*, respectively. The horizontal and vertical coordinates represent the retention times (RTs) and peak areas of the corresponding compounds, respectively.

To investigate the metabolic differences between yellow- and dark-seeded *B*. *juncea*, we selected eight *B*. *juncea* seed samples at different stages of development (20, 30, and 40 DAP), which showed obvious differences in seed coat color (Figure 1a). The relative metabolite contents of all of the samples were analyzed by UPLC-HESI-MS/MS in negative mode. A total of 13,574 base peak chromatograms identified by MS-DIAL version 4.18 software were produced from seeds at different developmental stages in yellow- and dark-seeded *B*. *juncea* (Figure 1b–d). Among them, 1440 could be identified and recorded by MS-DIAL ver. 4.18 software with a mass bank negative MS/MS database. We extracted 236 discernible base peak chromatograms according to their retention times (RT), accurate MS, and MS/MS spectral data together with the available key standards and previously reported information in this study, including 31 phenolic acids, 47 flavonoids, 17 glucosinolates, 38 lipid compounds, 69 other hydroxycinnamic acid compounds, and 34 unknown compounds (Figure 1e–g, Supplementary Table S1). Both the total base peak chromatograms and the base peak intensity of the extracted compounds differed between the dark mustard seeds and the yellow mustard seeds (Figure 1b–g).

### 2.2. Differential Metabolic Profiling Analysis Based on OPLS-DA

All of the peak areas of the discernible chromatograms were calculated using Xcalibur 3.1 software. The PCA score plot revealed a clear separation between the developing seeds from the yellow- and dark-seeded *B*. *juncea*, which was indicated by PCA1 (32%) and PCA2 (21%) (Supplementary Figure S1a), suggesting a significantly different metabolic profile among them, with a dynamic change accumulation during seed development. Meanwhile, the biological replicates of the same sample types showed a good stability and high reliability through the UPLC-HESI-MS/MS detection (Supplementary Figure S1a). 

To identify metabolites that were differentially abundant between the yellow- and dark-seeded *B*. *juncea*, we performed differential analysis on the samples from three developmental stages based on OPLS-DA. The OPLS-DA model compared the metabolite content of the samples in pairs to evaluate the differences the between yellow- and dark-seeded *B*. *juncea* at 20 DAP (R2X = 0.488, R2Y = 0.979, Q2 = 0.915), 30 DAP (R2X = 0.453, R2Y = 0.989, Q2 = 0.958) and 40 DAP (R2X = 0.408, R2Y = 0.996, Q2 = 0.977). All Q2 values of the model were close to 1, and all blue Q2 values to the left are lower than the original points to the right (Supplementary Figure S2a–c), indicating that these models could be used to further screen for differentially abundant metabolites. 

**Figure 2:**
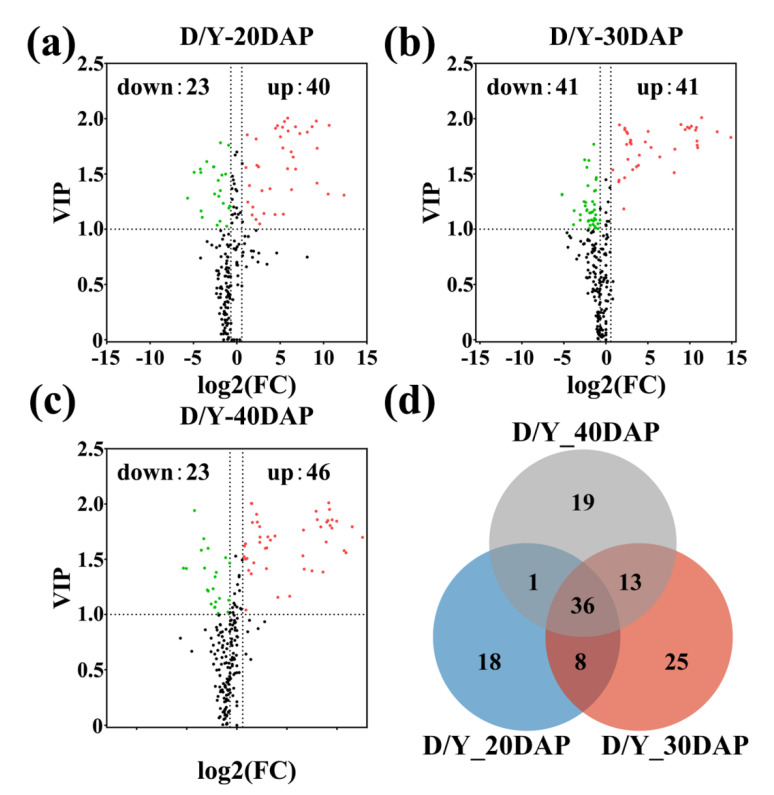
Identification of common differentially abundant metabolites in developing seeds of *B*. *juncea*. (**a–c**) Volcano plots of differentially abundant metabolites. Each point in a volcano plot represents a metabolite; the abscissa represents the logarithm of the quantitative difference multiples of a metabolite in two samples, and the ordinate represents the variable importance in project (VIP) value. The green dots represent down-regulated differentially abundant metabolites, the red dots represent up-regulated differentially abundant metabolites, and the black dots represent metabolites with no difference. (**d**) Venn diagram of differentially abundant metabolites in developing seeds of B. juncea. DAP = days after pollination.

We conducted a subsequent analysis of the differential metabolites that accumulated in the yellow- versus the dark-seeded *B*. *juncea*, with a VIP value ≥ 1, *p* value < 0.05, and a fold change value ≥ 1.6 or ≤ 0.625 for each group (Supplementary Table S2). As shown in the scatter diagram (Figure 2a–c), there were 63 significantly differentially abundant metabolites at 20 DAP (23 down-regulated, 40 up-regulated), 82 at 30 DAP (41 down-regulated, 41 up-regulated), and 70 at 40 DAP (23 down-regulated, 47 up-regulated). In addition, the principal component analysis (PCA) of the metabolite profiles showed that these metabolite profiles were obviously distinct at the three stages development of both the yellow- and dark-seeded varieties (Supplementary Figure S1b), and the metabolite profiles at 30 DAP and 40 DAP were closer than the profiles at 20 DAP in the yellow-seeded variety, indicating that the metabolite contents have dynamic variation during seed development. Meanwhile, the Venn diagram shows that 36 common differentially abundant metabolites were observed at all three stages (Figure 2d, Supplementary Table S3). As expected, there were 17 different metabolites in the flavonoid group, which is more than any other group (Table 1). At 40 DAP, epicatechin (243.94-fold), [DP2]-1 (549.83-fold), [DP2]-2 (263.83-fold), [DP3]-2 (643.99-fold), [DP3]-1 (1926.20-fold), and [DP4] (7.948-fold) were produced in greater concentrations in the dark-seeded variety than in the yellow-seeded variety, respectively (Table 1, Supplementary Table S4). In addition, the eriodictyol contents (615.06-fold) had higher accumulation levels in the dark-seeded variety than in yellow-seeded variety. Our results support that epicatechin and PAs are important factors affecting seed coat formation in *B*. *juncea*.

### 2.3. Metabolite Accumulation Patterns

**Figure 3:**
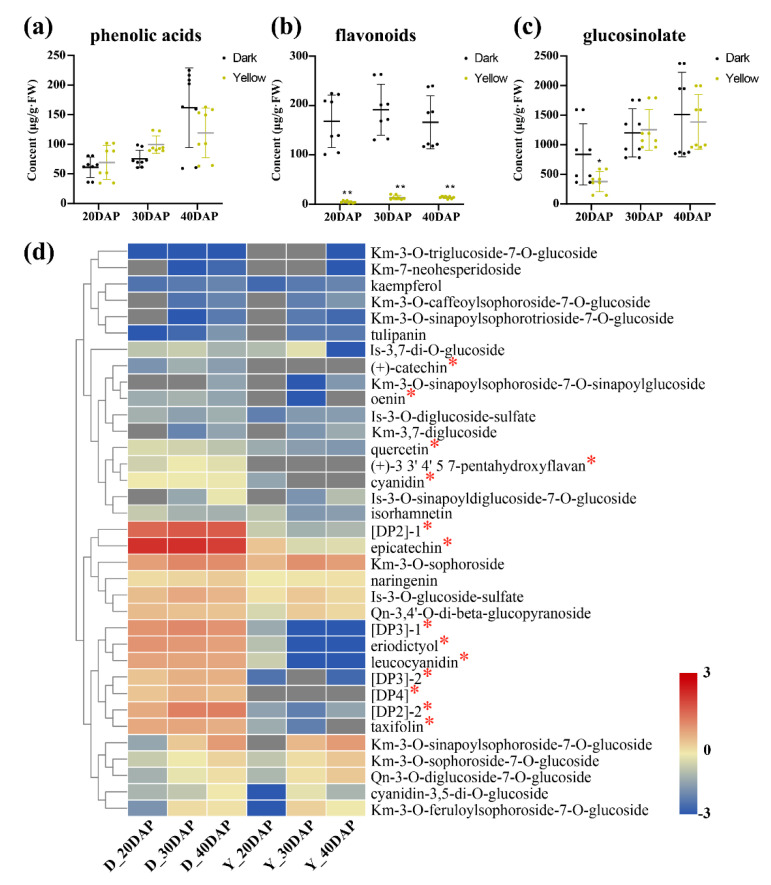
Analysis of metabolite accumulation patterns in *B. juncea*. (**a**–**c**) Distribution and accumulation pattern of phenolic acids (**a**), flavonoids (**b**), and glucosinolates (**c**) in yellow- and dark-seeded *B. juncea*. The horizontal and vertical coordinates represent the content of the constituent and the developmental stage, respectively. Error bars represent the SDs of the replications among the biological samples in this study. Statistical significance was calculated using Student’s *t*-test: *, *p* < 0.05; **, *p* < 0.01, respectively. (**d**) The accumulation patterns of flavonoid compounds in yellow- and dark-seeded *B. juncea*. Red indicates high abundance; blue indicates relatively low metabolite abundance. The bar represents the log_2_ contents (μg/g FW). DAP, days after pollination. The red asterisk indicates significantly different metabolites at three stages. DP2, procyanidin B; DP3, procyanidin C; DP4, procyanidin D.

To investigate the dynamic accumulation of the differential metabolites, these compounds were quantified from the calibration curve of corresponding or similar standard compounds. In this study, 92 metabolites could be relatively quantified using 9 commercial standards in B. juncea (Supplementary Table S1). Furthermore, we subjected the remaining 78 metabolites to PCA analysis after omitting the trace contents (<0.01 µg/g FW), which included 22 phenolic acids, 39 flavonoids, and 17 glucosinolates. The PCA results were similar to those obtained by peak-surface analysis (Supplementary Figure S1b) and revealed clear separation between the dark- and yellow-seeded samples as well as the samples from different developmental stages (Supplementary Figure S1b, Supplementary Table S4). In terms of seed development, the contents of glucosinolates and phenolic acids tended to accumulate in yellow- and dark-seeded *B*. *juncea* (Figure 3a,c), while the total flavonoid contents were significantly different (*p* < 0.01) between the yellow- and dark-seeded *B*. *juncea* during seed development (Figure 3b). These results indicate that flavonoids might be involved in the determination of seed coat color in *B*. *juncea*. 

**Figure 4:**
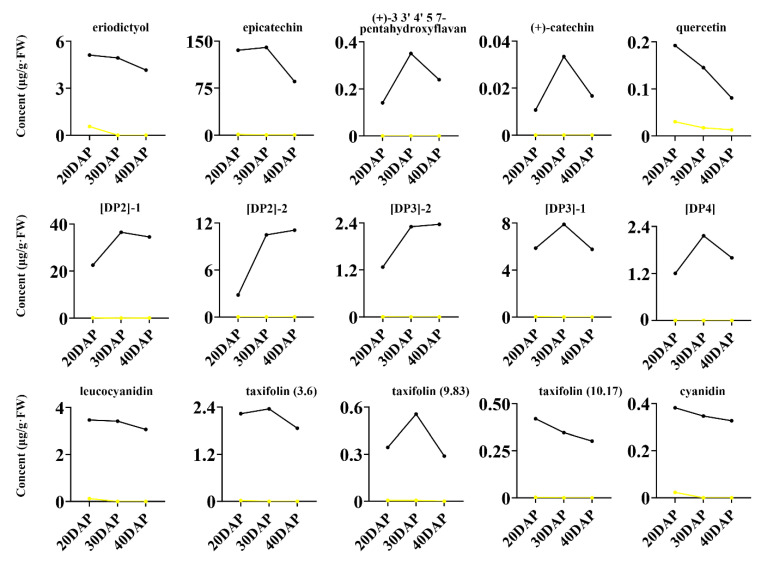
Qualitative and quantitative analysis of the common differentially abundant metabolites in yellow- and dark-seeded *B. juncea*. Accumulation of differentially abundant metabolites during seed development in yellow- and dark-seeded *B. juncea*. The black and yellow lines represent dark- and yellow-seeded *B. juncea*, respectively. The title number represents the code of proposed compounds and the related information is listed in Supplementary Table S1. DAP, days after pollination. DP2, procyanidin B; DP3, procyanidin C; DP4, procyanidin D.

To determine whether the dark seed coat color is caused by flavonoids, we analyzed 16 soluble flavonoids that were differentially abundant between yellow- and dark-seeded *B*. *juncea* seeds throughout development (Figure 4). Among them, Is-3-*O*-glucoside-7-*O*-glucoside (<0.01 µg/g FW) and oenin (0.02 µg/g FW) were omitted because they were present in trace amounts. These common differentially abundant metabolites showed various accumulation patterns during seed development in yellow- and dark-seeded *B*. *juncea*, and their levels were higher in the dark variety seeds than in the yellow *B*. *juncea* seeds throughout development (Figure 3d). For example, the larger amount of components, including epicatechin (139.975 µg/g FW), [DP2]-1 (36.52 µg/g FW), [DP2]-2 (10.495 µg/g FW), and [DP3]-1 (7.893 µg/g FW), were abundant in the dark-seeded variety at 30 DAP (Supplementary Table S4, Supplementary Figure S3). Our results indicate that the differentially abundant epicatechin and PAs might be associated with the seed coat coloration of *B*. *juncea*.

### 2.4. Transcriptome Analysis of Flavonoid Biosynthesis Genes in B. juncea

**Figure 5:**
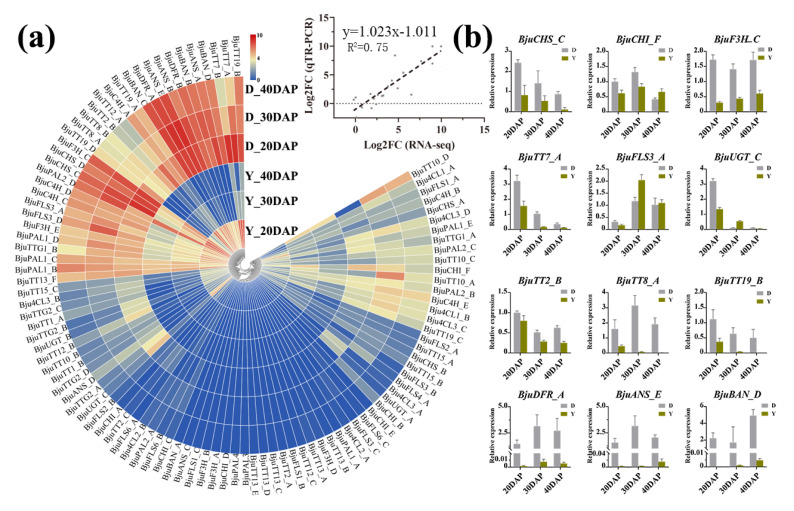
Analysis of the expression pattern of flavonoid genes in yellow and dark *B. juncea* seeds at various developmental stages. (**a**) Expression profiles of the flavonoid genes in yellow- and dark-seeded *B. juncea* at 20, 30, and 40 days after pollination (DAP). The scale bar denotes the log_2_ (FPKM + 1). The heatmap was generated using TBtools (version 1.055). The color represents relative gene expression levels. (**b**) RT-qPCR validation of flavonoid gene expression from the RNA-seq data at 20, 30, and 40 DAP. The candidate genes were named using the species abbreviation of the source organism (italicized), the gene family name, and the capital letter for the identified homologous, e.g., *BjuTT1_A*.

Flavonoids are responsible for the coloration of fruits, flowers, and seeds, and some genes regulating flavonoid biosynthesis have been found in *Brassica* species [35,55]. Therefore, we performed RNA-seq analysis to elucidate the differences in the expression patterns of flavonoid biosynthesis genes during seed development in yellow- and dark-seeded *B*. *juncea*. Based on the flavonoid biosynthesis gene and protein sequences of 31 *A*. *thaliana* obtained from the TAIR10 database (Supplementary Table S5), a total of 101 homologous genes were identified in this study (Supplementary Table S5, Supplementary Figure S4), and the functionally relevant amino acid residues of these identified genes had highly conserved positions (Supplementary Table S6). Subsequently, we investigated their transcript levels during seed development in yellow- and dark-seeded *B*. *juncea* using RNA-seq analysis (BioProject ID: PRJNA723131). To validate RNA-Seq results, 12 flavonoid biosynthesis genes with seed coat color formation were selected for RT-qPCR analysis, which had high expression levels with obvious differences (Figure 5b). Linear regression analysis indicated that the fold changes for gene expression investigated by RT-qPCR and RNA-Seq data were significantly positively correlated (R2 = 0.75; Figure 5a), suggesting that these results were reliable.

According to the FPKM values of 101 flavonoid biosynthesis genes, 25 of these had almost no expression in *B*. *juncea*, and different copies of homologous genes displayed different expression profiles (FPKM < 1, Figure 5a, Supplementary Table S7), possibly suggesting that they possess different biological functions. Consistently, most of the structural genes (*BjuCHSs*, *BjuCHIs*, *BjuF3Hs*, *BjuDFRs*, *BjuANSs*, and *BjuBANs*) were more abundant in the dark-seeded than in yellow-seeded *B*. *juncea* seeds, while *BjuCHI_D*, *BjuCHI_E*, *BjuF3H_A*, *BjuF3H_B*, *BjuANS_C*, and *BjuBAN_A* had almost no expression in the *B*. *juncea* seeds (Figure 5a, Supplementary Table S6), supporting that these homologous copies are likely not related to seed coat color formation in *B*. *juncea*. In addition, *BjuFLS3_A* showed a contrary expression pattern, which had relatively high expression levels in the yellow-seeded variety, and other *FLSs* were highly expressed in the dark-seeded variety (Figure 5). Among them, structural genes (*BjuCHSs*, *BjuCHIs*, and *BjuF3Hs*), also named the EBGs [36], play a role in the common precursors of flavonoids [31,32,33,48], indicating that these structural genes may determine the accumulation levels of the common precursors of flavonoids. However, structural genes, *BjuDFRs*, *BjuANSs*, and *BjuBANs*, leading to anthocyanin and proanthocyanidin [35,36,37,38] were expressed at very low levels in yellow-seeded *B*. *juncea* seeds, and they were expressed at much higher levels in dark-seeded *B*. *juncea* seeds; meanwhile, the transcription levels of *BjuTT19* also displayed an obvious difference, with a continuous decline during seed development in both the yellow- and dark-seeded varieties (Figure 5). Our findings suggest that trace PA accumulation in the yellow seeds is associated with the low expression level of flavonoid biosynthesis genes, in accordance with previous results [35,56].

**Figure 6:**
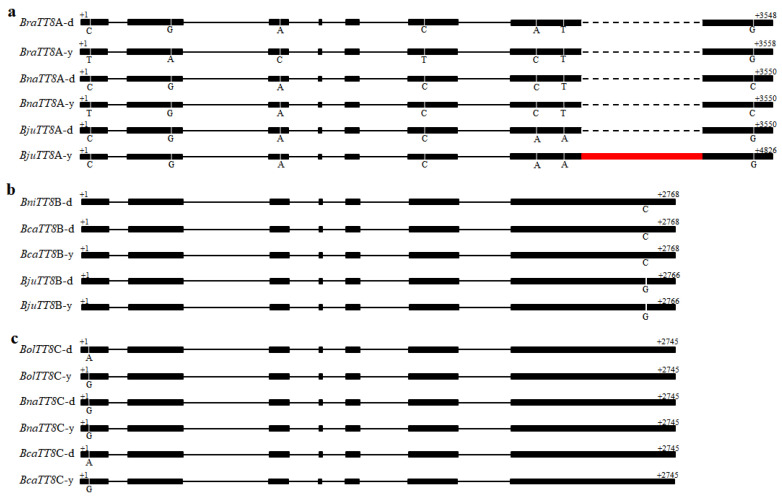
Structural organization of *TT8* sequences from *Brassica* species. (**a**) Structure of *TT8* sequences from *B*. *rapa*, *B*. *napus*, and *B*. *juncea*; (**b**) Structure of *TT8* sequences from *B*. *nigra*, *B*. *carinata,* and *B*. *juncea*; (**c**) Structure of *TT8* sequences from *B*. *oleracea*, *B*. *napus,* and *B*. *carinata*. The black rectangles represent the exons. The red rectangle represents the insertion in *BjuTT8*A-y. The lowercase letter y and d at the end of gene name represent the yellow- and dark-seeded seed coat color, respectively. Details of the sequence and structural organization of *TT8* are shown in Supplementary File S1.

Previous results showed that LBGs were also positively activated by MBW complexes [39,40,41,48]. In this study, the dynamic expression of *BjuTT2*, *BjuTT8*, and *BjuTTG1* showed similar expression patterns with LBGs (Figure 6). Among them, studies have also shown that *TT8* is required to positively activate the transcription of LBGs, which is involved in the seed coat color of *B*. *juncea* [8], *B*. *rapa* [12], and *B*. *napus* [7,11,13]. However, the yellow-seeded trait had different variations in *Brassica* species. Therefore, we further compared the sequences of *TT8* from dark-seeded *B*. *nigra* and yellow- and dark-seeded *B*. *rapa*, *B*. *oleracea*, *B*. *napus*, *B*. *juncea*, and *B*. *carinata* (Figure 6, Supplementary File S1). The results showed that the length of the *TT8* varied among *Brassica* species from 3548 bp in dark-seeded *B*. *rapa* (*BraTT8A*-d) to 4826 bp in yellow-seeded *B*. *juncea* (*BjuTT8A*-y). An insertion of 1276 bp was located in exon 7 of the yellow-seeded *B*. *juncea* sequence (*BjuTT8A*-y) (Figure 6a, Supplementary File S1). Five SNPs were also identified in the exons (exons 1, 2, 3, 6, and 7) of the *TT8A* gene between yellow- and dark-seeded *B*. *rapa*, but only one SNP was detected between the yellow- and dark-seeded *B*. *napus* (Figure 6a, Supplementary Figure S5). Many other SNPs and indels were also found among the *TT8A* sequences (Supplementary Figure S5), but the extron sequences of *TT8B* and *TT8C* copies were highly conserved among these *Brassica* species. For example, one SNP was detected in the seventh extron of *BjuTT8B* and *BcaTT8B* between *B*. *juncea* and *B*. *carinata*, and one SNP in first extrons of *BolTT8C* and *BcaTT8C* was detected in yellow- and dark-seeded *B*. *oleracea* and *B*. *carinata* (Figure 6b,c, Supplementary Figures S6 and S7). Our results support the idea that variants in the *TT8A* gene might be a crucial factor for seed coat color in *Brassica* species. Additionally, the gene *BjuTT19s*, which encodes a protein reported to be involved in procyanidin transport [57,58], also had relatively low expression levels in yellow- *B*. *juncea* (Figure 5a), supporting the low accumulation of PAs in the yellow-seeded *B*. *juncea* (Table 1). Our results thus suggest that genes for the accumulation of PAs play an important role in seed coat color of *B*. *juncea*. 

**Figure 7:**
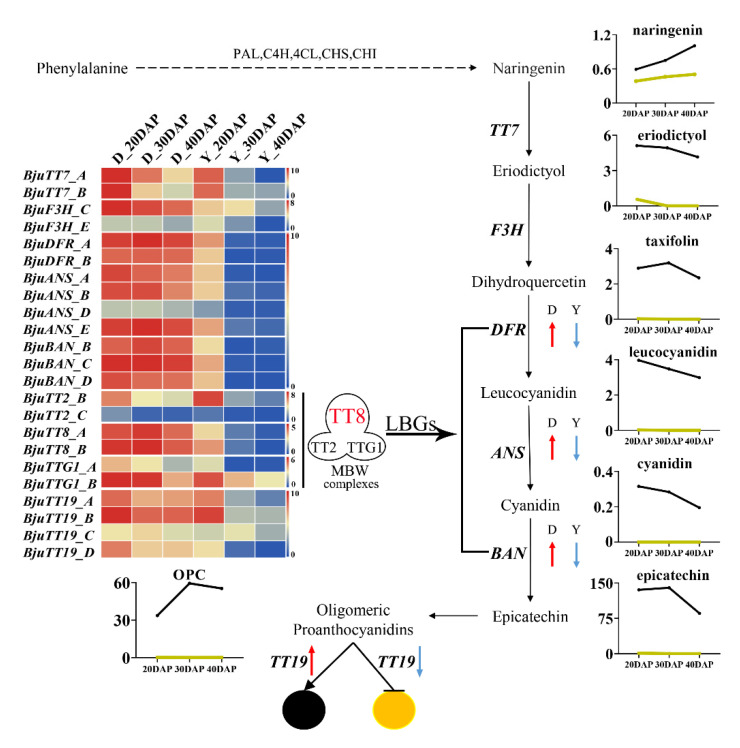
Schematic diagram of the flavonoid biosynthetic pathway in *B*. *juncea*. The scale bar denotes the log2 (FPKM + 1). Table 1. 055). The color represents relative gene expression levels.

To further explore the regulatory network of flavonoids in yellow- and dark-seeded *B*. *juncea*, we analyzed the identified flavonoids using UPLC-HESI-MS/MS and the expression patterns of differentially expressed genes related to flavonoid biosynthesis by means of RNA-seq analysis (BioProject ID: PRJNA723131). At the first dedicated step of the flavonoid biosynthetic pathway, the expression levels of *BjuCHIs*, *BjuF3Hs*, and *BjuTT7s* showed different expression patterns in yellow- and dark-seeded *B*. *juncea* (Figure 5a), and the corresponding metabolites (i.e., naringenin, eriodictyol, quercetin isorhamnetin, and its corresponding derivatives) also had relatively low accumulation levels in the yellow seeds (Supplementary Table S4), indicating that these EBGs play important roles in promoting the production of enzymes to catalyze the metabolism of the substrates. Importantly, we found that the epicatechin and procyanidin oligomers ([DP2]-1, [DP2]-2, [DP3]-2, [DP3]-1, and [DP4]) had trace content (< 0.01 µg/g FW) in yellow seeds. Transcriptome data showed that *BjuDFR*, *BjuANS*, and *BjuBAN* had almost no expression in the yellow seeds, indicating that these genes might be suppressed in promoting yellow seed pigmentation. We therefore speculated that the lower expression levels of the LBGs (*BjuDFR*, *BjuANS*, and *BjuBAN*) resulted in a deficiency of epicatechin and PAs, which predominantly determined the seed coat color formation in *B*. *juncea*. Meanwhile, *BjuTT19* encoding glutathione transferase were remarkably down-regulated or not expressed in the yellow-seeded variety (Figure 5), indicating that the accumulation of PAs is inhibited in the yellow-seeded variety. Overall, a diagram of the flavonoid metabolomics pathways in *B*. *juncea* shows that the expression patterns of most flavonoid biosynthesis genes were consistent with the metabolite accumulation patterns (Figure 7), while epicatechin and PAs contribute to dark seed coat color formation in *B*. *juncea*.

## 3. Discussion

*Brassica juncea*, a crop plant cultivated worldwide, produces black, brown, or yellow seeds; these colors are produced by flavonoids, the largest group of specialized metabolites, including flavonols, flavones, isoflavones, flavanols, and PAs [6,8,35,42]. To date, many phenolic compounds have been investigated in the *Brassica* species [13,14,16,59,60,61], but they are still not well understood in *B*. *juncea* seeds. Cultivated *Brassica* species include three diploid species (*B*. *rapa*, *B*. *nigra*, and *B*. *oleracea*) and three amphidiploid species (*B*. *juncea*, *B*. *napus*, and *B*. *carinata*), which are closely interrelated and well understood by the theory of the ‘triangle of U’ [62]. Therefore, these results provide important clues to discover the biochemical mechanisms of the flavonoid biosynthesis pathway in *B*. *juncea*. In addition, most metabolites involved in the seed coat color, such as epicatechin, isorhamnetin, kaempferol, quercetin, and their derivatives, are widely detected in *Brassica* crops [13,14,16,27,52,59,60,61,63], suggesting that they might share a common metabolic pathway. In this study, a total of 1440 major metabolic base peak chromatograms using UPLC-HESI-MS/MS analysis, and 236 metabolites were well-identified in the developmental seeds of *B*. *juncea*, including 31 phenolic acids, 47 flavonoids, 17 glucosinolates, 38 lipid compounds, 69 other hydroxycinnamic acid compounds, and 34 unknown compounds (Figure 1e–g, Supplementary Table S1). Previous studies highlighted the role of PAs in the seed coat of the *Brassica* species [13,14,16,27,52,59,60,61]. Based on levels of the identified metabolic compounds, yellow- and dark-seeded *B*. *juncea* could be distinguished (Supplementary Figure S1). Among them, we found that flavonoids are the predominately different metabolites between the yellow- and dark-seeded varieties (Table 1, Figure 3b), suggesting that they were involved in the modulating seed coat color formation of *B*. *juncea*. Meanwhile, we found that the greatest number of differentially expressed compounds was detected at 30 DAP (Figure 2b), indicating that most different metabolites might be catalyzed in this stage [13]. Importantly, 36 differential metabolic compounds were significantly accumulated throughout seed development (Figure 2d, Table 1), suggesting that they might be closely associated with the formation of seed coat color in *B*. *juncea*. Among them, 17 common differentially abundant metabolites were grouped into flavonoids (Table 1) such as eriodictyol, epicatechin, [DP2]-2, [DP3]-2, [DP3]-1 and [DP4]. Moreover, eriodictyol is a substrate of F3H/TT6 that catalyzes the synthesis of the dihydroflavonols that ar involved in plant coloration [64,65]; epicatechin and PAs have often been reported to be involved in the seed coat color of *Brassica* species [11,13,14,16]. In addition, 13 of 36 unknown compounds were detected and showed significant differences in the yellow- and dark-seeded *B*. *juncea* seeds (Table 1, Supplementary Table S1). In summary, our findings found that epicatechin and its corresponding derivatives were important in the seed coat color variation of *B*. *juncea* seeds, but additional studies are needed to verify whether these unknown metabolites are responsible for the seed coat color of *B*. *juncea* seeds.

In plants, flavonoids are widely distributed and contribute to coloration, which is well understood in *Arabidopsis* and *Brassica* [10,30,35,37,38,43,52,57,66]. The release of the genomic data for *B*. *juncea* [1,41], *B*. *rapa* [67,68], and *B*. *nigra* [69] offered us a chance to elucidate the flavonoid biosynthetic pathway at a genome-wide level in *B*. *juncea*. In this study, 101 flavonoid biosynthesis genes were identified based on the released *B*. *juncea* genome sequence (Supplementary Table S5). In addition, the color of the flowers, fruits, and seeds are mainly determined by the flavonoids, which are the most widely used metabolic pathway in plants [10,55]. Herein, we compared the expression patterns of these flavonoid biosynthesis genes during seed development in yellow- and dark-seeded *B*. *juncea* (Figure 5). In general, 25 genes were expressed at low levels in both yellow- and dark-seeded *B*. *juncea*, while 76 genes showed higher expression in the dark-seed variety than in the yellow-seed variety (Figure 5a). Interestingly, we found that transcripts of the structural genes (*BjuCHSs*, *BjuCHIs*, *BjuF3Hs*, and *BjuTT7s*), the first dedicated step of the flavonoid biosynthetic pathway, were more abundant in dark-seeded than in yellow-seeded *B*. *juncea*, while the LBGs, such as *BjuDFRs*, *BjuANSs*, and *BjuBANs*, had hardly or almost no expression in yellow-seeded *B*. *juncea* (Figure 5a), indicating that these were important factors affecting seed coat formation in *B*. *juncea* [35]. Similarly, numerous studies on the mechanism of seed coat color in *Brassica* species have shown that seed coat color is mainly influenced by the lower expression of the genes that regulate the flavonoid biosynthesis pathway [7,11,13,14,70,71]. Therefore, we speculate that these genes used for PA biosynthesis lead to the different seed coat color variation in *B*. *juncea*. 

To examine flavonoid accumulation during seed development, we performed further correlation analysis between the transcriptome and metabolite profiling, and the expression patterns of some flavonoid biosynthetic genes showed a high association with the accumulation of some flavonoids and their derivatives. Previous studies showed that TT8 is a central component of MYB–bHLH–WD repeat complexes and is essential in the flavonoid biosynthetic pathway [8,12,43]. As expected, *BjuTT8* also had significantly higher expression levels in the dark-seeded variety than in yellow-seeded variety of *B*. *juncea* (Figure 7), consistent with previous findings that *TT8* accounted for increasing levels of anthocyanin accumulation in plants [12,35,38,43]. In addition, recent studies suggest that *BjuTT8* could promote the expression of *BjuDFR* and *BjuTT19* thus increasing anthocyanin accumulation and conferring the purple color of *B*. *juncea* leaves [38], which also acts as a major regulator of flavonoid biosynthesis pathways in *Brassica* plants [42,72,73,74]. Herein, *BjuTT8* and LBGs (*BjuDFRs*, *BjuANSs*, and *BjuBANs*) are also closely related to epicatechin and its derivatives in this study (Figure 7). In Chinese cabbage, the yellow-seeded trait is due to a deletion of *TTG1* [75] and in yellow sarson, a trait is due to the insertion of a transposable element in *TT8* [12]. Therefore, we further investigated the variation of *TT8* structure and identified substantial variation among the sequences in *B*. *rapa*, *B*. *napus*, and *B*. *juncea* (Figure 6a, Supplementary Figures S5–S7). In fact, the alignment of the *BjuTT8A* gene revealed an insertion of 1276 bp in exon 7, similar to published results (Supplementary Figure S5) [8], but there is no mutation in *BjuTT8B* gene members, indicating that *TT8* homologous genes may have different functions in different *B*. *juncea* varieties. In addition, a large insertion was detected in the yellow-seeded *B*. *rapa* [12], while we found the obviously SNP variation between the yellow- and dark-seeded plants by alignment of *BraTT8A* (Figure 6a, Supplementary Figure S5) but no presence of *BnaTT8A*, suggesting that *TT8A* showed different functional variations in *B*. *rapa*, *B*. *juncea*, and *B*. *napus*. However, the sequences of the *TT8* genes were highly conserved in *B*. *nigra*, *B*. *oleracea*, *B*. *napus*, and *B*. *carinata* (Figure 6b,c, Supplementary Figures S6 and S7). Therefore, our findings propose that *TT8A* genes play important roles in the stability of the seed coat color among the *Brassica* species, which has undergone functional differentiation during the evolution of the *Brassica* genome. 

In this study, we also identified other differences that shed light on seed color in *B*. *juncea*. For example, four copies of the transport gene *BjuTT19* were also significantly up-regulated in dark-seeded *B*. *juncea* relative to yellow-seeded *B*. *juncea* (Figure 5), and the low levels of metabolites were detected in yellow-seeded *B*. *juncea* (Supplementary Table S2). Hence, we hypothesized that low expression levels of *BjuTT19* may be involved in the seed coat color through the inhibition of anthocyanin/PAs transport in the flavonoid biosynthesis pathway of *B*. *juncea*. In addition, the metabolites oenin and tulipanin were preliminarily identified in the developing seeds using accurate MS and MS/MS spectral data, which was catalyzed by the flavanone 3’,5’-hydroxylase (F3′5′H) [76]. Previous results have shown that multiple F3′5′H evolutions from F3′H have occurred in dicotyledonous plants [76] and have shown that F3′H and F3′5′H belong to cytochrome P450-dependent enzymes (CYP), a diverse class of heme-containing oxidases that are present in all types of organisms [77], indicating that the F3′H may have a redundant role in the control of the 3’5’-hydroxylated flavonoids of *Arabidopsis* and *Brassica* plants. Therefore, abundant metabolites were detected (Supplementary Table S1) and need to be further identified. In summary, our results improve our understanding of the molecular mechanism of seed coat color formation in Brassica species and demonstrate that combining metabolome and transcriptome analysis is an effective method for identifying key genes involved in flavonoid biosynthesis.

## 4. Materials and Methods

### 4.1. Plant Materials and Culture Conditions

Eight yellow- and dark-seeded *B*. *juncea* accessions (Figure 1a, Supplementary Table S8) were used in this study and were obtained from The Research Institute of Oil Crops in Guizhou. All eight accessions were sown in late September 2019 and grown under normal environmental conditions in the experimental field in Guiyang, Guizhou, China, located at the coordinates of 26°44′ N, 106°43′ W and at an altitude of 1250 m. Each accession was grown in randomized complete blocks with three rows at each site (0.4 m between rows and 0.2 m between plants) under normal field conditions. To investigate the dynamic metabolites of the seeds during maturation, the flowers were marked with different colored wool to keep the seeds at the same development stages together. At 20, 30, and 40 days after pollination (DAP), the immature seeds were gently harvested from five individual plants and pooled into a 5-mL centrifuge tube, immediately frozen in liquid nitrogen, and then stored at −80 °C until total RNA and crude metabolite extraction.

### 4.2. Chemicals and Calibration Curve

All commercial standards, including caffeic acid, ferulic acid, epicatechin (Ep), kaempferol (Km), isorhamnetin (Is), p-coumaric acid, quercetin (Qn), sinapic acid, and sinigrin of least LC/MS grade (purity > 99%) were purchased from Sigma-Aldrich Trading Co., Ltd. (Shanghai, China). The stock solutions for each standard were prepared individually in aqueous methanol and stored in the dark at −20 °C. A mixed stock solution for each standard was then prepared in aqueous methanol at 5 mg L⁻^1^. Spiked calibration curves at eight levels (0.001, 0.005, 0.01, 0.05, 0.20, 0.50, 1.0, and 2.0 mg L⁻^1^) were prepared in triplicate for calibration curve construction [14,25]. 

### 4.3. Metabolite Extraction and Analysis

Metabolites were extracted using the previously described methods [14] with slight modifications. Fresh seeds (100 mg) were weighed in pre-cooled microfuge tubes with liquid nitrogen (2-mL, Eppendorf, Germany), quickly crushed into powder, and homogenized in an aqueous solution of formic acid (0.1% (*v*/*v*)) in aqueous methanol (1 mL; 80% (*v*/*v*)). Samples were then sonicated (KQ-100E, Kunshan, China) for 1 h, and the crude extracts were centrifuged for 15 min at 10,000 × g. Subsequently, the precipitate was extracted again using the same method. Finally, the pooled supernatants were filtered via a 0.22-μm nylon filter and analyzed using ultra-high-performance liquid chromatography–heated electrospray ionization–tandem mass spectrometry (UPLC-HESI-MS/MS). All samples were analyzed in four biological duplicates.

After filtering, 10 μL samples were analyzed using a Dionex UltiMateTM 3000 UHPLC system (Thermo Fisher Scientific, Waltham, MA, USA) connected to a Thermo Scientific Q-Exactive System equipped with an S-Lens ionizer source (Thermo Scientific, USA) in negative mode. An Acquity UPLC BEH C18 chromatography column (2.1 i.d. × 150 mm, 1.7-μm particle size) (Waters, Ireland) was used with a guard column (Acquity UPLC BEH C18 1.7 μm VanGuardTM Pre-Column 2.1 × 5 mm, Waters, Ireland) controlled at 30 °C. The composition of the mobile phase was as follows: solutions A (0.1% (*v*/*v*) formic acid in H_2_O) and B (0.1% (*v*/*v*) formic acid in acetonitrile). The following mobile phase gradient was employed: 0–2 min, 5–10% solution B; 2–10 min, 10–25% solution B; 10–15 min, 25–50% solution B; 15– 0 min, 50–95% solution B; 20–23 min, 95% solution B; 23–23.5 min, 95–5% solution B; and 23.5–28 min, 5% solution B. The flow rate was set to 0.300 mL min^−1^. The spectra were recorded using full scan mode, covering a mass range from *m*/*z* 100 to 1500. The operation parameters were as follows: source voltage, 3.5 kV; sheath gas, 35 (arbitrary units); auxiliary gas, 10 (arbitrary units); sweep gas, 0 (arbitrary units); and capillary temperature, 350 °C.

All raw data were converted for free for use in the MS data analysis tool using the ABF (Analysis Base File) converter (http://www.reifycs.com/AbfConverter/index.html, accessed on 22 March 2020). The datasets were then analyzed using MS-DIAL version 4.18 software with a mass bank negative database (http://prime.psc.riken.jp/compms/msdial/main.html#MSP, accessed on 22 March 2020) [78]. Meanwhile, the raw UPLC-HESI-MS/MS data were further analyzed using Xcalibur 3.1 software. Discernible base peak areas from the built-in analyst quantitation wizard were manually corrected. Finally, the compounds were confirmed by comparing their retention times, accurate MS, and MS/MS spectral data together with the commercial standards and previously reported information [14,25]. Based on their mass spectrum, the concentrations of the identified compounds were quantified using calibration curves obtained with the corresponding or similar standards in this study. 

### 4.4. Statistical Analysis

Principal component analysis (PCA) and t-tests were performed on the web-based sever Metabolite Sets Enrichment Analysis 4.0 (MSEA 4.0 or MetaboAnalyst 4.0; http://www.metaboanalyst.ca, accessed on 22 March 2020) [79]. Multiple regression orthogonal partial least-squares discriminant analysis (OPLS-DA) was performed using SIMCA V14.1 (https://umetrics.com/, accessed on 22 March 2020) with the default parameters. The significantly differential metabolites were screened using the variable importance in the projection (VIP) value of the first principal component in the OPLS-DA model combined with fold change (FC). The metabolites with variable importance in projection value (VIP) ≥ 1, *p* value < 0.05, and fold change (FC) of ≥1.6 (up-regulated) or ≤0.625 (down-regulated) were considered to be differential metabolites. Volcano plots were used to filter metabolites of interest based on the log2(FC) of the metabolites [80,81]. Data were expressed as the mean ± standard deviation (SD) of four biological replicates.

### 4.5. Identification of Flavonoid Biosynthesis Genes in B. juncea

A total of 31 gene sequences in *A*. *thaliana* related to flavonoid biosynthesis were downloaded from The Arabidopsis Information Resource (TAIR) database (https://www.arabidopsis.org/, accessed on 22 July 2020). The *B*. *juncea* reference genome sequence (Bju15: *Brassica juncea* V1.5) and gene sequences from BRAD (http://brassicadb.cn, accessed on 22 July 2020) were used to identify the flavonoid biosynthesis genes in *B*. *juncea*. We used the flavonoid biosynthesis gene and protein sequences of 31 *A*. *thaliana* to align with the *B. juncea* genome and protein sequences using BLASTn and BLASTp with a cutoff E-value of ≤1 × 10^−20^, respectively. The homologous gene pairs were confirmed through multiple alignments performed using Geneious 4.8.5 software (http://www.geneious.com/, accessed on 22 July 2020; Biomatters, Auckland, New Zealand), which showed the syntenic analysis results across *B*. *juncea* and each *A*. *thaliana*. The conserved motifs of flaovonoid biosynthesis genes (Supplementary Figure S4) were characterized using Multiple Em for Motif Elicitation (MEME) 4.11.4 (http://meme-suite.org/tools/meme, accessed on 22 July 2020) with the following parameters: any repetitions; maximum 5 motifs; and 6 and 300 residues width of each motif [82]. Genes with an E-value of <1 × 10^−20^ were used for further analysis. Eventually, these identified flavonoid biosynthesis genes were confirmed through the knowledge-based identification of pathway enzymes (KIPEs; https://github.com/bpucker/KIPEs, accessed on 22 July 2020) [83]

### 4.6. Transcriptome Sequencing Analysis of Differentially Expressed Genes in the Flavonoid Pathway

To better understand the pathways of flavonoid biosynthesis in *B*. *juncea*, we performed BLASTP searches in the Brassica database (*B*. *juncea* genome V1.5; http://brassicadb.cn, accessed on 22 July 2020) [1] using the protein sequences related to flavonoid synthesis genes in *Arabidopsis* [28,30,84] as queries. Subsequently, the expression profiles of flavonoid biosynthesis genes were further revealed using transcriptome analysis based on RNA-seq data. For each developmental stage of each *B*. *juncea* line (20, 30 and 40 DAP), total RNA was extracted from the seed samples (approximately 100 mg) stored at −80 °C using an EZ-10 DNAaway RNA Mini-Preps Kit (Sangon Biotech Co., Ltd., Shanghai, China). The RNA quality was validated using 1% agarose gel, and the purity, concentration, and integrity were confirmed using a Nanodrop spectrophotometer (Thermo Fisher Scientific, Inc., Worcester, MA, USA) and an Agilent 2100 Bioanalyzer (Agilent Technologies, Inc., Santa Clara, CA, USA), respectively. The mRNA was enriched using the NEBNext^®^ Poly(A) mRNA Magnetic Isolation Module, and a cDNA library was constructed using the recommended program, NEBNext® mRNA Library Prep Master Mix Set, following manufacturer’s recommendations. The libraries were sequenced on an Illumina HiSeq2000 sequencing platform by Novogene Bioinformatic Technology Co. Ltd. (Tianjin, China). Two biological replicates were used for the transcriptome analysis.

The adapter sequences and unknown or low-quality reads were filtered using Trimmomatic version 0.32 [85], and clean reads were mapped to the *B*. *juncea* reference genome sequence [1] (Bju15: *Brassica juncea* V1.5; http://brassicadb.cn, accessed on 22 July 2020) using HISAT (hierarchical indexing for spliced alignment of transcripts; version 2.1.0) with the default parameters (phred33-p5—sensitive—no-discordant—no-mixed-I 1-X 1000) [86], and the number of mapped reads was quantified using HTseq [87]. Analysis of differential gene expression between the yellow- and dark-seeded varieties were performed with the DESeq2 (version 1.16.1) [88] with R version 3.2.3 (Supplementary Table S9). Genes with an adjusted *p*-value that was ≤ 0.05 and fold changes ≥ 2 were considered as differentially expressed. The gene expression profiles were evaluated using FPKM (fragments per kilo base of exon model per million) values. The heatmap was generated using TBtools (version 1.055) [89].

### 4.7. Reverse-Transcription Quantitative PCR (RT-qPCR) Analysis 

To validate the transcriptome data and to characterize the flavonoid genes that were differentially expressed in the developing seeds of yellow- and dark-seeded B. juncea, total RNA was isolated from the samples using a DNA away RNA Mini-Prep Kit (Sangon Biotech, Shanghai, China). Subsequently, the cDNAs were synthesized using an RNA PCR Kit (AMV, v3.0) based on the manufacturer’s protocols (Takara, Dalian, China). The cDNA was subjected to RT-qPCR analysis using SYBR qPCR SuperMix Plus (NovoStart) on a Bio-Rad CFX96 Real-Time System (Bio-Rad Laboratories, Hercules, CA, USA), as previously described [13]. Tonoplastic intrinsic proteins-41 (TIPS-41) was used as a reference gene to normalize the gene expression levels via the 2^−∆∆Ct^ method [90]. Three biological replicates were performed for all experiments. The specific primer sequences used in this study were obtained from the RT-qPCR Primer Database [91] and are listed in Supplementary Table S10.

### 4.8. Gene Cloning and Multi-Sequence Analysis

The genomic DNA and open reading frame of *TT8* were amplified using the genomic DNA and cDNA of dark-seeded *B*. *nigra* and yellow- and dark-seeded *B*. *rapa*, *B*. *oleracea*, *B*. *napus*, *B*. *juncea*, and *B*. *carinata* as templates, respectively. Subsequently, the sequences of *TT8* were subjected to multiple sequence alignments using ClustalW software (version 2.0) with default settings [92]. PCR, cloning of PCR production and the selection of positive clones were performed in three biological repetitions. Positive clones were sequenced using M13F/M13R primer (Tsingke Biological Technology Company, Beijing, China). The specific primer sequences used in this study are listed in Supplementary Table S10.

## 5. Conclusions

In this study, 236 metabolite compounds were identified from *B*. *juncea* seeds, including 31 phenolic acids, 47 flavonoids, 17 glucosinolates, 38 lipids, 69 other hydroxycinnamic acid compounds, and 34 novel unknown compounds. 35 of them showed significant differences in yellow and dark *B*. *juncea* seeds, especially with regard to flavonoids. In addition, 101 homologous flavonoid genes were identified, and their expression patterns were investigated using RNA-Seq and RT-qPCR analysis, most of them showed significant expression levels in yellow and dark *B*. *juncea* seeds. Importantly, comparative transcriptome and metabolome analyses of yellow and dark *B*. *juncea* revealed high consistent changes in flavonoid biosynthesis genes (*BjuDFR*, *BjuANS* and *BjuBAN*, *BjuTT8* and *BjuTT19*) and the levels of flavonoids (epicatechin and its derivatives). Further, *TT8* plays a crucial role in the seed coat color of *Brassica* species. Our results provide new insights into the understanding of the synthesis and accumulation of flavonoids in *B. juncea* seeds as well as lay a solid biological foundation for breeding improvements for the *Brassica* species.

## Figures and Tables

**Table 1:** Common differentially abundant metabolites in yellow- and dark-seeded *B*. *juncea* at three developmental stages.

ID	Type ^1^	Proposed Compound ^2^	RT ^3^	Average Mass	D/Y-20DAP			D/Y-30DAP			D/Y-40DAP		
					FC ^4^	VIP-Value ^5^	*p*-Value	FC	VIP-Value	*p*-Value	FC	VIP-Value	*p*-Value
30	1	2,5-dihydroxybenzoic acid	3.49	153.0182	608.61	1.417	0.007	281.69	1.417	0.023	106.51	1.411	0.069
105	2	eriodictyol	6.98	287.0565	45.634	1.974	0	2732.3	1.974	0	615.06	1.951	0
106	2	epicatechin	6.97	289.0723	60.921	1.889	0	494.35	1.889	0	243.94	1.934	0
108	2	(+)-3,3′,4′,5,7-pentahydroxyflavan	5.58	289.0728	4.677	1.817	0	4.753	1.817	0	2.729	2.005	0
110	2	(+)-catechin	13.16	289.0728	3.430	1.130	0.170	9.129	1.130	0	4.856	1.795	0
118	2	quercetin	13.12	301.0365	2.310	1.854	0.002	4.801	1.854	0	2.937	1.833	0
182	2	oenin	12.69	491.1215	79.572	1.358	0	15.839	1.358	0	7.814	1.415	0
197	2	[DP2]-2	9.52	577.1362	41.518	1.135	0.265	1168.5	1.135	0	263.83	1.858	0
198	2	[DP2]-1	6.30	577.1363	114.18	1.536	0	527.47	1.869	0	549.83	1.851	0
233	2	[DP3]-2	9.41	865.2006	110.57	1.544	0	1783.3	1.544	0	643.99	1.805	0
235	2	[DP3]-1	7.47	865.2016	57.917	1.547	0	1854.9	1.547	0	1926.2	1.559	0
249	2	[DP4]	7.84	1153.2640	5.695	1.564	0	8.064	1.564	0	7.948	1.603	0
261	2	Is-3-*O*-glucoside-7-*O*-glucoside	5.16	639.1576	8.740	1.142	0.215	25.923	1.142	0	8.558	1.703	0
309	2	leucocyanidin	3.6	305.0656	38.740	1.923	0	1650	1.900	0	9.504	1.780	0
310	2	taxifolin	3.6	303.0533	39.700	1.736	0	9818	1.880	0	11.54	1.790	0
311	2	taxifolin	9.83	303.0533	22.080	1.914	0	41.79	1.740	0	6.659	1.760	0
312	2	taxifolin	10.17	303.0533	32.850	1.837	0	3.086	1.940	0	2.323	1.70	0
313	2	cyanidin	3.6	285.0397	280.200	1.878	0	7.383	1.790	0	8.928	1.660	0
33	4	pimelic acid	6.3	159.0653	0.239	1.298	0.001	0.173	1.298	0	0.276	1.011	0.003
171	4	LysoPE 16:0	15.36	452.2786	0.261	1.072	0.010	0.027	1.072	0	0.108	1.420	0
7	5	4-Chlorophenol	3.25	127.0024	0.340	1.234	0	0.425	1.234	0	0.573	1.129	0.033
129	5	thymidine-5′-monophosphate	13.13	321.0446	2.090	1.558	0.119	4.714	1.558	0	10.239	1.673	0
287	6	un13	12.11	461.1082	14.225	1.366	0	32.625	1.366	0	4.923	1.653	0
288	6	un14	11.91	461.1083	90.449	1.653	0	85.958	1.653	0	96.966	1.531	0
294	6	un20	3.47	368.0614	154.720	1.866	0	5.568	1.866	0	1693.3	1.578	0
295	6	un21	3.6	403.0335	109.03	1.928	0	5.966	1.928	0	3.931	1.907	0
296	6	un22	3.54	437.1093	58.71	2.006	0	985.41	2.006	0	581.94	2.01	0
297	6	un23	5.09	447.1416	413.2	1.929	0	14.701	1.929	0	38.925	1.165	0
298	6	un24	5.68	447.1416	1613.9	1.94	0	12.554	1.94	0	17.16	1.156	0
299	6	un25	3.29	468.042	576.27	1.979	0	1897.9	1.979	0	736.6	1.858	0
300	6	un26	3.56	524.1769	25.956	1.936	0	5.574	1.936	0	2.827	2.005	0
301	6	un27	7.07	579.1514	613.78	1.733	0	30570	1.733	0	6037.4	1.699	0
302	6	un28	9.63	623.1410	5.023	1.578	0	847.06	1.578	0	334.75	1.793	0
303	6	un29	6.43	645.1224	77.343	1.7	0	697.36	1.7	0	506.53	1.836	0
304	6	un30	8.09	720.1609	5292.1	1.308	0	299.54	1.308	0	183.1	1.395	0
305	6	un31	10.18	720.1609	1484.9	1.317	0	1971.1	1.317	0	392.68	1.385	0

^1^ Type 1, phenolic acids; type 2, flavonoids; type 4, lipid compounds; type 5, other hydroxycinnamic acid compounds; type 6, unknown compounds. **^2^** Is= isorhamnetin; DP, degree of polymerization of the epicatechin unit; DP2, procyanidin B; DP3, procyanidin C; DP4, procyanidin D; un, unknown metabolites. ^3^ RT, retention time (min). ^4^ FC, fold change. ^5^ VIP, variable importance in projection.

## Data Availability

The data presented in this study are available upon request from the corresponding author. The RNA seq Illumina paired-end reads of the transcriptome for this study have been submitted to NCBI BioProject PRJNA723131.

## Supplementary Materials

The following are available online at https://www.mdpi.com/article/10.3390/ijms22137215/s1.
