# Supplementary material for: Metabolite Profiling and Transcriptome Analysis Provide Insight into Seed Coat Color in Brassica juncea
Source: Int J Mol Sci. 2021 Jul 5;22(13):7215. doi: 10.3390/ijms22137215 (PMC8268557; doi:10.3390/ijms22137215)
Supplement: Supplementary file 1 [file ijms-22-07215-s001.zip › ijms-1245090-SI/Supplementary Figure S1-S4.pdf]

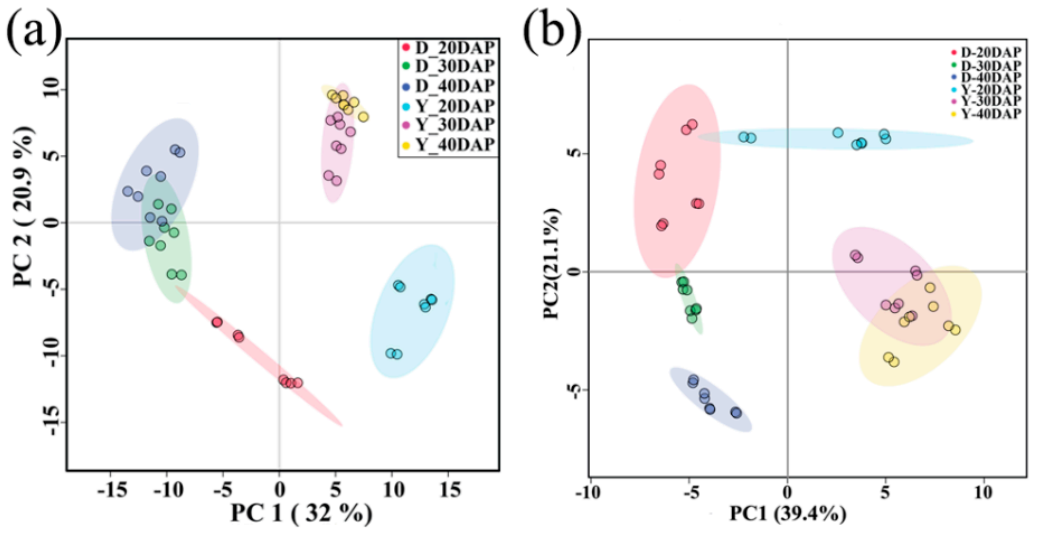

Supplementary Figure S1. Differential flavonoid metabolite analysis on the basis of OPLS-DA and principal component (PCA). (**a**) The OPLS-DA score plot of different yellow- and dark-seeded *B. juncea*. Different colored dots represent the different stages of seed. (**b**) Principal component analysis of 78 metabolites detected in yellow- and dark-seeded *B. juncea*. Different colored dots represent the different seed development stages.

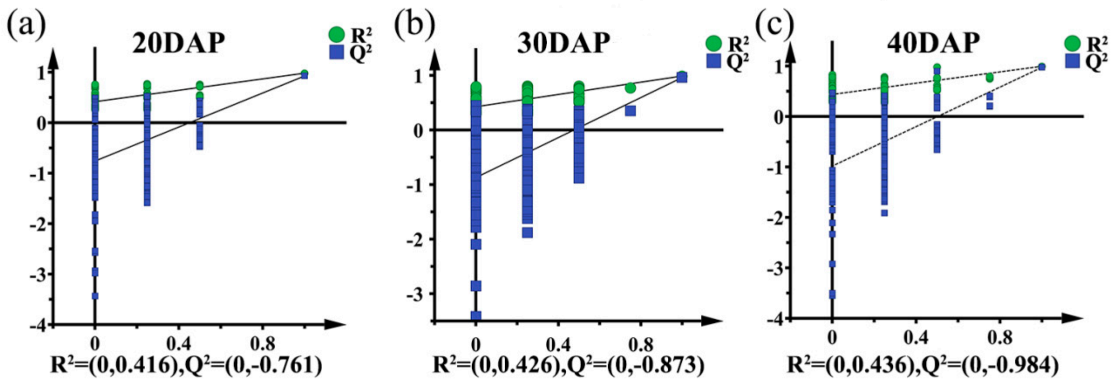

Supplementary Figure S2. The partial least squares-discriminant analysis (OPLS-DA). (**a-c**) OPLS-DA model plots for the permutation test at 20, 30 and 40 DAP, respectively.

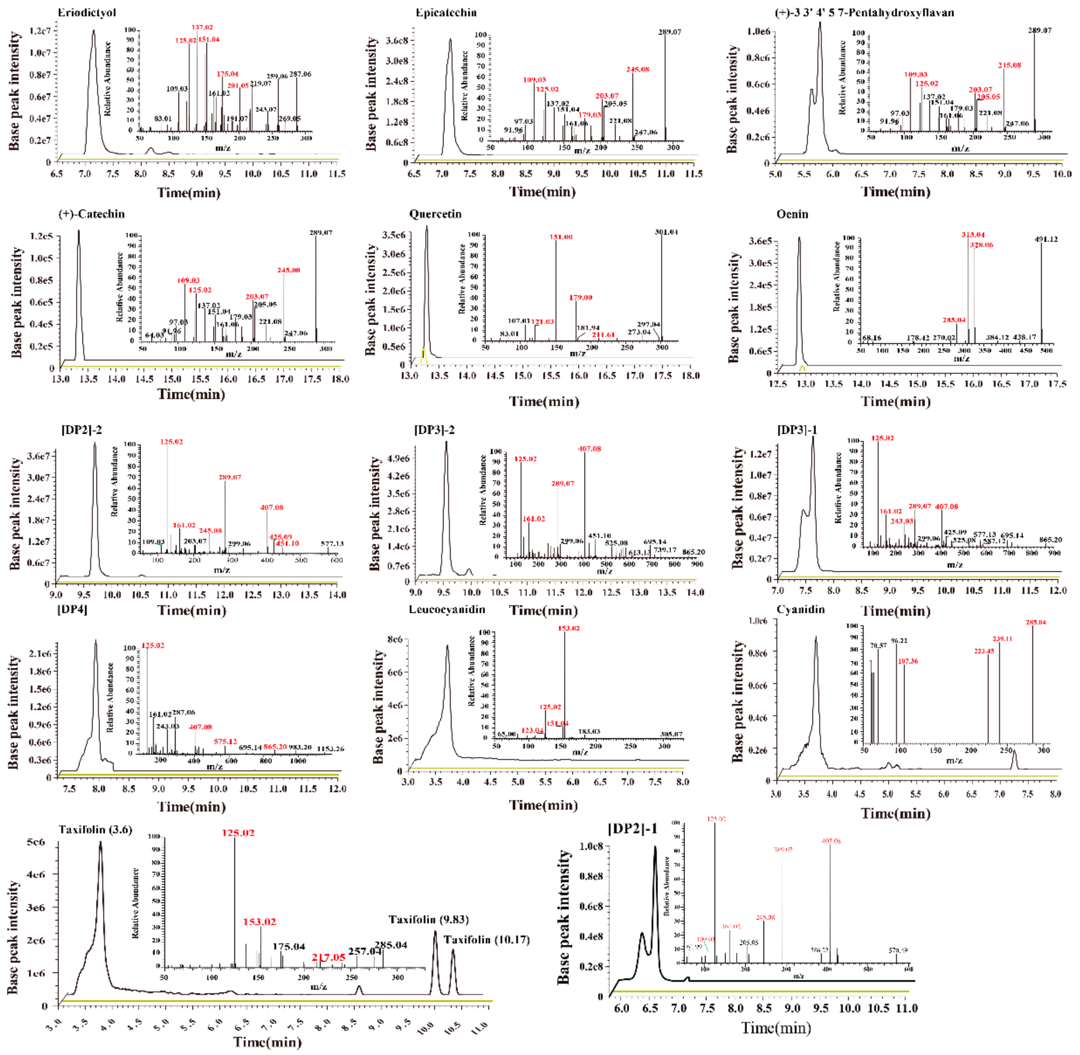

Supplementary Figure S3. UPLC-HESI-MS/MS chromatograms of significant constituents in yellow- and dark-seeded *B. juncea*. DP2, procyanidin B; DP3, procyanidin C; DP4, procyanidin D.

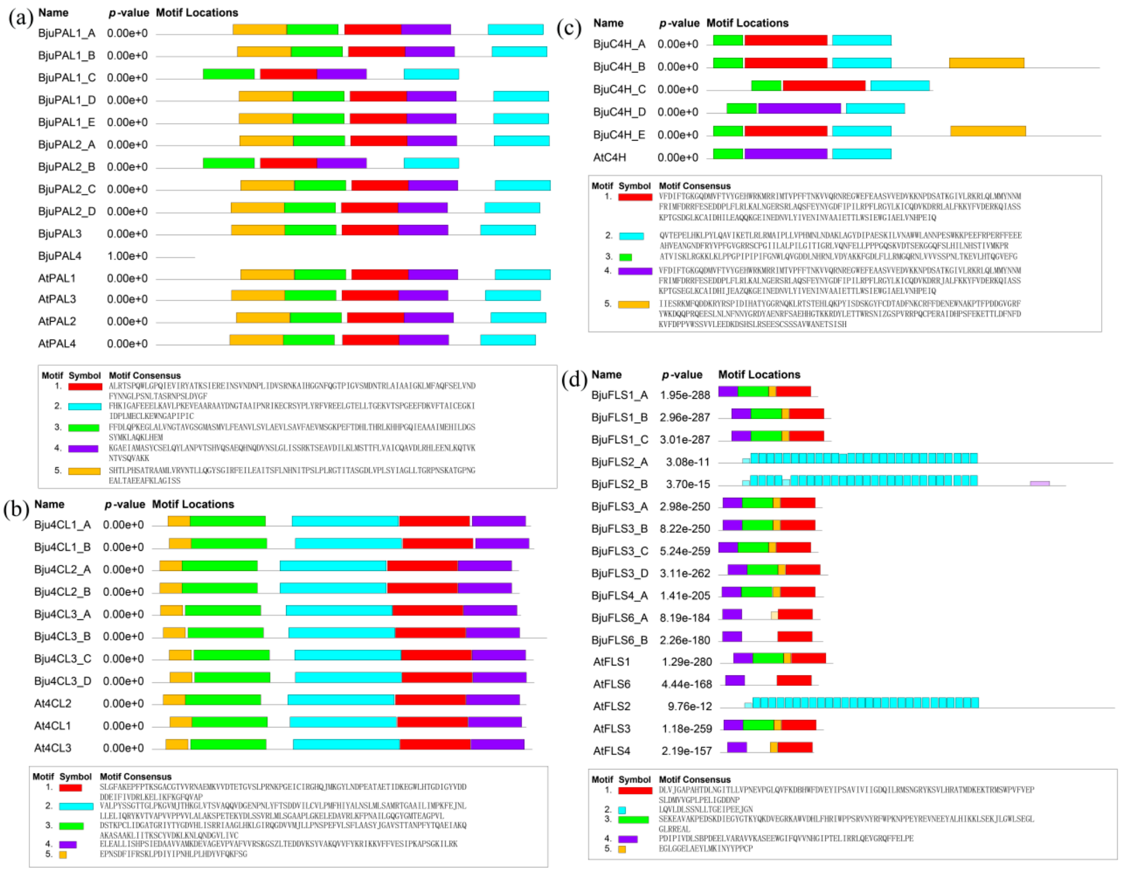

Supplementary Figure S4. Motif patterns of the flavonoid biosynthesis genes in this study

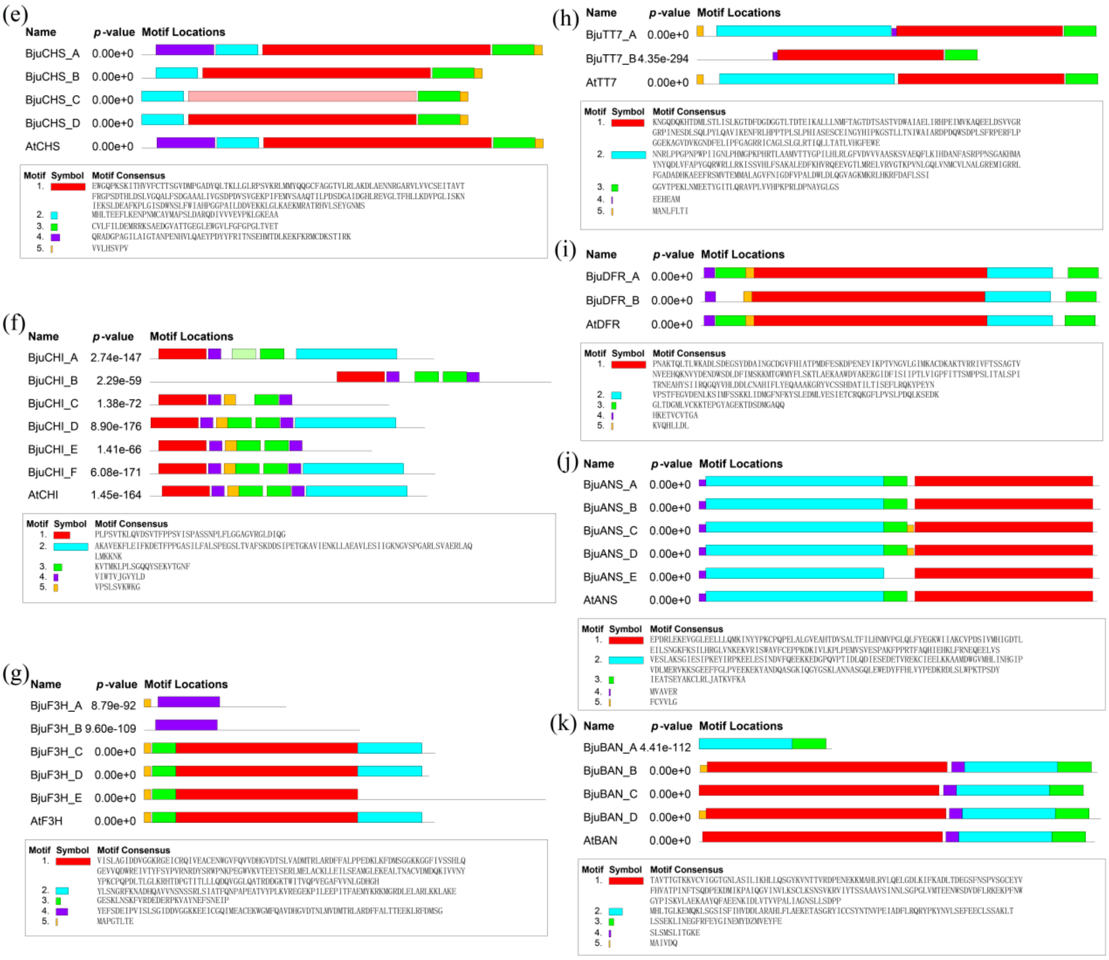

Supplementary Figure S4. Continually

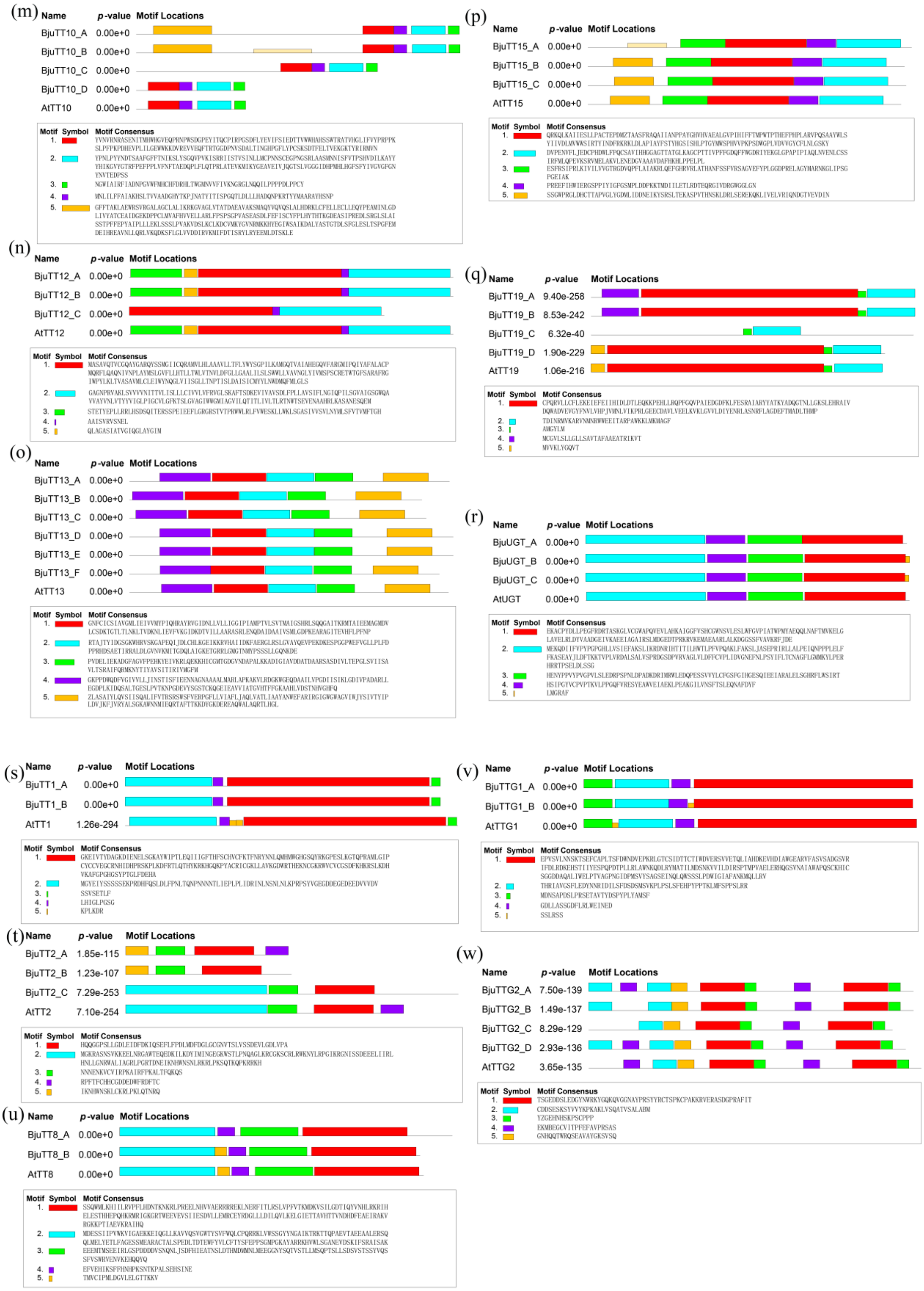

Supplementary Figure S4. Continually
