# Supplementary material for: Metabolite Profiling and Transcriptome Analysis Provide Insight into Seed Coat Color in Brassica juncea
Source: Int J Mol Sci. 2021 Jul 5;22(13):7215. doi: 10.3390/ijms22137215 (PMC8268557; doi:10.3390/ijms22137215)
Supplement: Supplementary file 1 [file ijms-22-07215-s001.zip › ijms-1245090-SI/Supplementary Figure S5.pdf]

```
extron1
                                                                       *
BrTT8A-b_g : ATGGATGAATTAAGTATCATACCGTTATGGAAAGTGATCGGGGCTGAGAAAGAAGAGATTCAAGGGCTACTTAAGGCGGTGGTGCAATCTGTGGGG :  96
BrTT8A-y_g : ATGGATGAATTAAGTATTATACCGTTATGGAAAGTGATCGGGGCTGAGAAAGAAGAGATTCAAGGGCTACTTAAGGCGGTGGTGCAATCTGTGGGG :  96
BnTT8A-b_g : ATGGATGAATTAAGTATCATACCGTTATGGAAAGTGATCGGGGCTGAGAAAGAAGAGATTCAAGGGCTACTTAAGGCGGTGGTGCAATCTGTGGGG :  96
BnTT8A-y_g : ATGGATGAATTAAGTATTATACCGTTATGGAAAGTGATCGGGGCTGAGAAAGAAGAGATTCAAGGGCTACTTAAGGCGGTGGTGCAATCTGTGGGG :  96
BjTT8A-d_g : ATGGATGAATTAAGTATCATACCGTTATGGAAAGTGATCGGGGCTGAGAAAGAAGAGATTCAAGGGCTACTTAAGGCGGTGGTGCAATCTGTGGGG :  96
BjTT8A-y_g : ATGGATGAATTAAGTATCATACCGTTATGGAAAGTGATCGGGGCTGAGAAAGAAGAGATTCAAGGGCTACTTAAGGCGGTGGTGCAATCTGTGGGG :  96
BjTT8A-y_r : ATGGATGAATTAAGTATCATACCGTTATGGAAAGTGATCGGGGCTGAGAAAGAAGAGATTCAAGGGCTACTTAAGGCGGTGGTGCAATCTGTGGAG :  96
             ATGGATGAATTAAGTAT ATACCGTTATGGAAAGTGATCGGGGCTGAGAAAGAAGAGATTCAAGGGCTACTTAAGGCGGTGGTGCAATCTGTGGgG

                100                                                        *
BrTT8A-b_g : TGGACTTATAGTGTCTTCTGGCAACTTTGTCCTCAACGAAGGTTCACTTTCTTTTCATTTTATCCATCTCTCACAGTATATAAAGCAATATAGTAA : 192
BrTT8A-y_g : TGGACTTATAGTGTCTTCTGGCAACTTTGTCCTCAACGAAGGTTCACTTTCTTTTCATTTTATCCATCTCTCACAGTATATAAAGCAATATAGTAA : 192
BnTT8A-b_g : TGGACTTATAGTGTCTTCTGGCAACTTTGTCCTCAACGAAGGTTCACTTTCTTTTCATTTTATCCATCTCTCACAGTATATAAAGCAATATAGTAA : 192
BnTT8A-y_g : TGGACTTATAGTGTCTTCTGGCAACTTTGTCCTCAACGAAGGTTCACTTTCTTTTCATTTTATCCATCTCTCACAGTATATAAAGCAATATAGTAA : 192
BjTT8A-d_g : TGGACTTATAGTGTCTTCTGGCAACTTTGTCCTCAACGAAGGTTCACTTTCTTTTCATTTTATCCATCTCTCACAGTATATAAAGCAATATAGTAA : 192
BjTT8A-y_g : TGGACTTATAGTGTCTTCTGGCAACTTTGTCCTCAACGAAGGTTCACTTTCTTTTCATTTTATCCATCTCTCACAGTATATAAAGCAATATAGTAA : 192
BjTT8A-y_r : TGGACTTATAGTGTCTTCTGGCAACTTTGTCCTCAACGAAGGTTCACTTTCTTTTCATTTTATCCATCTCTCACAGTATATAAAGCAATATAGTAA : 192
             TGGACTTATAGTGTCTTCTGGCAACTTTGTCCTCAACGAAGGTTCACTTTCTTTTCATTTTATCCATCTCTCACAGTATATAAAGCAATATAGTAA

                200                                       extron2*
BrTT8A-b_g : TCTTTATTAATTATAATAAGTAGAAGTGACTAAATGTTAAATCGATTAGGAAATTGATGTGGAGTAGTGGATACTACAACGGCGCAATAAAGACTA : 288
BrTT8A-y_g : TCTTTATTAATTATAATAAGTAGAAGTGACTAAATGTTAAATCGATTAGGAAATTGATGTGGAGTAGTGGATACTACAACGGCGCAATAAAGACTA : 288
BnTT8A-b_g : TCTTTATTAATTATAATAAGTAGAAGTGACTAAATGTTAAATCGATTAGGAAATTGATGTGGAGTAGTGGATACTACAACGGCGCAATAAAGACTA : 288
BnTT8A-y_g : TCTTTATTAATTATAATAAGTAGAAGTGACTAAATGTTAAATCGATTAGGAAATTGATGTGGAGTAGTGGATACTACAACGGCGCAATAAAGACTA : 288
BjTT8A-d_g : TCTTTATTAATTATAATAAGTAGAAGTGACTAAATGTTAAATCGATTAGGAAATTGATGTGGAGTAGTGGATACTACAACGGCGCAATAAAGACTA : 288
BjTT8A-y_g : TCTTTATTAATTATAATAAGTAGAAGTGACTAAATGTTAAATCGATTAGGAAATTGATGTGGAGTAGTGGATACTACAACGGCGCAATAAAGACTA : 288
BjTT8A-y_r : TCTTTATTAATTATAATAAGTAGAAGTGACTAAATGTTAAATCGATTAGGAAATTGATGTGGAGTAGTGGATACTACAACGGCGCAATAAAGACTA : 288
             TCTTTATTAATTATAATAAGTAGAAGTGACTAAATGTTAAATCGATTAGGAAATTGATGTGGAGTAGTGGATACTACAACGGCGCAATAAAGACTA

                        300                                                *
BrTT8A-b_g : GAAAGACAACTCAGCCGGCGGAAGTTACGGCTGAAGAGGCTGCGTCGGAGAGAAGCCAACAGCTCATGGAGCTTTACGAGACGCTTTTTGCTGGAG : 384
BrTT8A-y_g : GAAAGACAACTCAGCCGGCGGAAGTTACGGCTGAAGAGGCTGCGTCGGAGAGAAGCCAACAGCTCATGGAGCTTTACGAGACGCTTTTTGCTGGAG : 384
BnTT8A-b_g : GAAAGACAACTCAGCCGGCGGAAGTTACGGCTGAAGAGGCTGCGTCGGAGAGAAGCCAACAGCTCATGGAGCTTTACGAGACGCTTTTTGCTGGAG : 384
BnTT8A-y_g : GAAAGACAACTCAGCCGGCGGAAGTTACGGCTGAAGAGGCTGCGTCGGAGAGAAGCCAACAGCTCATGGAGCTTTACGAGACGCTTTTTGCTGGAG : 384
BjTT8A-d_g : GAAAGACAACTCAGCCGGCGGAAGTTACGGCTGAAGAGGCTGCGTCGGAGAGAAGCCAACAGCTCATGGAGCTTTACGAGACGCTTTTTGCTGGAG : 384
BjTT8A-y_g : GAAAGACAACTCAGCCGGCGGAAGTTACGGCTGAAGAGGCTGCGTCGGAGAGAAGCCAACAGCTCATGGAGCTTTACGAGACGCTTTTTGCTGGAG : 384
BjTT8A-y_r : GAAAGACAACTCAGCCGGCGGAAGTTACGGCTGAAGAGGCTGCGTCGGAGAGAAGCCAACAGCTCATGGAGCTTTACGAGACGCTTTTTGCTGGAG : 384
             GAAAGACAACTCAGCCGGCGGAAGTTACGGCTGAAGAGGCTGCGTCGGAGAGAAGCCAACAGCTCATGGAGCTTTACGAGACGCTTTTTGCTGGAG

                            400                                                *
BrTT8A-b_g : AATCATCGATGGAAGCGAGGGCTTGCACAGCACTGTCGCCGGAGGATTTGACGGATCCTGAATGGTTTTATGTGCTGTGTTTCACTTACTCTTTCG : 480
BrTT8A-y_g : AATCATCGATGGAAGCGAGGGCTTGCACAGCACTGTCGCCGGAGGATTTGACAGATCCTGAATGGTTTTATGTGCTGTGTTTCACTTACTCTTTCG : 480
BnTT8A-b_g : AATCATCGATGGAAGCGAGGGCTTGCACAGCACTGTCGCCGGAGGATTTGACGGATCCTGAATGGTTTTATGTGCTGTGTTTCACTTACTCTTTCG : 480
BnTT8A-y_g : AATCATCGATGGAAGCGAGGGCTTGCACAGCACTGTCGCCGGAGGATTTGACGGATCCTGAATGGTTTTATGTGCTGTGTTTCACTTACTCTTTCG : 480
BjTT8A-d_g : AATCATCGATGGAAGCGAGGGCTTGCACAGCACTGTCGCCGGAGGATTTGACGGATCCTGAATGGTTTTATGTGCTGTGTTTCACTTACTCTTTCG : 480
BjTT8A-y_g : AATCATCGATGGAAGCGAGGGCTTGCACAGCACTGTCGCCGGAGGATTTGACGGATCCTGAATGGTTTTATGTGCTGTGTTTCACTTACTCTTTCG : 480
BjTT8A-y_r : AATCATCGATGGAAGCGAGGGCTTGCACAGCACTGTCGCCGGAGGATTTGACGGATCCTGAATGGTTTTATGTGCTGTGTTTCACTTACTCTTTCG : 480
             AATCATCGATGGAAGCGAGGGCTTGCACAGCACTGTCGCCGGAGGATTTGACgGATCCTGAATGGTTTTATGTGCTGTGTTTCACTTACTCTTTCG

                                500                                                *
BrTT8A-b_g : AACCTCCTTCTGGGTACAACAACTCTCTCTCTGTCTTCAAAGTTTTTTTTTTCTTTTCAAAAAGACTACTCAAAGTTTCTTAATTTGTCTTTTTCA : 576
BrTT8A-y_g : AACCTCCTTCTGGGTACAACAACTCTCTCTCTGTCTTCAAAGTTTTTTTTTTCTTTTCAAAAAGACTACTCAGAGTTTCTTAATTTGTCTTTTTCA : 576
BnTT8A-b_g : AACCTCCTTCTGGGTACAACAACTCTCTCTCTGTCTTCAAAGTTTTTTTTTTCTTTTCAAAAAGACTACTCAAAGTTTCTTAATTTGTCTTTTTCA : 576
BnTT8A-y_g : AACCTCCTTCTGGGTACAACAACTCTCTCTCTGTCTTCAAAGTTTTTTTTTTCTTTTCAAAAAGACTACTCAAAGTTTCTTAATTTGTCTTTTTCA : 576
BjTT8A-d_g : AACCTCCTTCTGGGTACAACAACTCTCTCTCTGTCTTCAAAGTTTTTTTTTTCTTTTCAAAAAGACTACTCAAAGTTTCTTAATTTGTCTTTTTCA : 576
BjTT8A-y_g : AACCTCCTTCTGGGTACAACAACTCTCTCTCTGTCTTCAAAGTTTTTTTTTTCTTTTCAAAAAGACTACTCAAAGTTTCTTAATTTGTCTTTTTCA : 576
BjTT8A-y_r : AACCTCCTTCTGGGTACAACAACTCTCTCTCTGTCTTCAAAGTTTTTTTTTTCTTTTCAAAAAGACTACTCAAAGTTTCTTAATTTGTCTTTTTCA : 576
             AACCTCCTTCTGGGTACAACAACTCTCTCTCTGTCTTCAAAGTTTTTTTTTTCTTTTCAAAAAGACTACTCAaAGTTTCTTAATTTGTCTTTTTCA

                                    600                                                *
BrTT8A-b_g : TCTGCTCTTAGAGAAGACAAAAATAGTATTGTGTGTTAAATGCGAATCACAAATACTATGGAAGCATTAAAGACAAACTGGGGAGTTTAAGTTACT : 672
BrTT8A-y_g : TCTGCTCTTAGAGAAGACAAAAATAGTATTGTGTGTTAAATGCGAGTCACAAATACTATGGAAGCATTAAAGACAAACTGGGGAGTTTAAGTTACT : 672
BnTT8A-b_g : TCTGCTCTTAGAGAAGACAAAAATAGTATTGTGTGTTAAATGCGAACCACAAATACTATGGAAGCATTAAAGACAAACTGGGGAGTTTAAGTTACT : 672
BnTT8A-y_g : TCTGCTCTTAGAGAAGACAAAAATAGTATTGTGTGTTAAATGCGAACCACAAATACTATGGAAGCATTAAAGACAAACTGGGGAGTTTAAGTTACT : 672
BjTT8A-d_g : TCTGCTCTTAGAGAAGACAAAAATAGTATTGTGTGTTAAATGCGAATCACAAATACTATGGAAGCATTAAAGACAAACTGGGGAGTTTAAGTTACT : 672
BjTT8A-y_g : TCTGCTCTTAGAGAAGACAAAAATAGTATTGTGTGTTAAATGCGAATCACAAATACTATGGAAGCATTAAAGACAAACTGGGGAGTTTAAGTTACT : 672
BjTT8A-y_r : TCTGCTCTTAGAGAAGACAAAAATAGTATTGTGTGTTAAATGCGAATCACAAATACTATGGAAGCATTAAAGACAAACTGGGGAGTTTAAGTTACT : 672
             TCTGCTCTTAGAGAAGACAAAAATAGTATTGTGTGTTAAATGCGAa CACAAATACTATGGAAGCATTAAAGACAAACTGGGGAGTTTAAGTTACT

                                        700                                                *
BrTT8A-b_g : GAAAGTAGAAATGTATTGAAGTTTGTAAAAACGTACACTTCATTTTGGTGAACCTAATTGGACCGTTGAGATTCTTATTGGTTTGTTTATTGATTA : 768
BrTT8A-y_g : GAAAGTAGAAATGTATTGAAGTTTGTAAAAACGTACACTTCATTTTGGTGAACATAATTGGACCGTTGAGATTCTTATTGGTTTGTTTATTGATTA : 768
BnTT8A-b_g : GAAAGTAGAAATGTATTGAAGTTTGTAAAAACGTACACTTCATTTTGGTGAACCTAATTGGACCGTTGAGATTCTTATTGGTTTGTTTATTGATTA : 768
BnTT8A-y_g : GAAAGTAGAAATGTATTGAAGTTTGTAAAAACGTACACTTCATTTTGGTGAACCTAATTGGACCGTTGAGATTCTTATTGGTTTGTTTATTGATTA : 768
BjTT8A-d_g : GAAAGTAGAAATGTATTGAAGTTTGTAAAAACGTACACTTCATTTTGGTGAACCTAATTGGACCGTTGAGATTCTTATTGGTTTGTTTATTGATTA : 768
BjTT8A-y_g : GAAAGTAGAAATGTATTGAAGTTTGTAAAAACGTACACTTCATTTTGGTGAACCTAATTGGACCGTTGAGATTCTTATTGGTTTGTTTATTGATTA : 768
BjTT8A-y_r : GAAAGTAGAAATGTATTGAAGTTTGTAAAAACGTACACTTCATTTTGGTGAACCTAATTGGACCGTTGAGATTCTTATTGGTTTGTTTATTGATTA : 768
             GAAAGTAGAAATGTATTGAAGTTTGTAAAAACGTACACTTCATTTTGGTGAACcTAATTGGACCGTTGAGATTCTTATTGGTTTGTTTATTGATTA

                                    800                                                *
BrTT8A-b_g : TCTAAAGTAGGAGCATATATAGATGATAAATGCATAACAAAGTGTGTTAGTTATCGGTATAAT-----------TAATGTTTTTTCTCTATGGAGG : 853
BrTT8A-y_g : TCTAAAGTAGGAGCATATATAGATGATAAATGCATATCAAAGTGTGTTAGTTATCGGTATAAT-----------TAATGTTTTTTCTCTATGGAGG : 853
BnTT8A-b_g : TCTAAAGTAGGAGCATATATAGATGATAAATGCATAACAAAGTGTGTTAGTTATCGGTATAAT-----------TAATGTTTTTTCTCTATGGAGG : 853
BnTT8A-y_g : TCTAAAGTAGGAGCATATATAGATGATAAATGCATAACAAAGTGTGTTAGTTATCGGTATAAT-----------TAATGTTTTTTCTCTATGGAGG : 853
BjTT8A-d_g : TCTAAAGTAGGAGCATATATAGATGATAAATGCATAACAAAGTGTGTTAGTTATCGGTATAAT-----------TAATGTTTTTTCTCTATGGAGG : 853
BjTT8A-y_g : TCTAAAGTAGGAGCATATATAGATGATAAATGCATAACAAAGTGTGTTAGTTATCGGTATAAT-----------TAATGTTTTTTCTCTATGGAGG : 853
BjTT8A-y_r : TCTAAAGTAGGAGCATATATAGATGATAAATGCATAACAAAGTGTGTTAGTTATCGGTATAATTAATCAAAATATAATGTTTTTTCTCTATGGAGG : 864
             TCTAAAGTAGGAGCATATATAGATGATAAATGCATAaCAAAGTGTGTTAGTTATCGGTATAAT           TAATGTTTTTTCTCTATGGAGG
```

```
                                                  900 extron3                                                 *
BrTT8A-b_g : AACAAATCAAAATATAATGTGGAAGTATTAATTTGTAGGATGCCAGGAAAAGCGTATGCGAGGAGGAAGCACATATGGCTAAGTGGTGCAAATGAG :  949
BrTT8A-y_g : AACAAATCAAAATATAATGTGGAAGTATTAATTTGTAGGATGCCAGGAAAAGCGTATGCGAGGAGGAAGCACATATGGCTACGTGGTGCAAATGAG :  949
BnTT8A-b_g : AACAAATCAAAATATAATGTGGAAGTATTAATTTGTAGGATGCCAGGAAAAGCGTATGCGAGGAGGAAGCACATATGGCTAAGTGGTGCAAATGAG :  949
BnTT8A-y_g : AACAAATCAAAATATAATGTGGAAGTATTAATTTGTAGGATGCCAGGAAAAGCGTATGCGAGGAGGAAGCACATATGGCTAAGTGGTGCAAATGAG :  949
BjTT8A-d_g : AACAAATCAAAATATAATGTGGAAGTATTAATTTGTAGGATGCCAGGAAAAGCGTATGCGAGGAGGAAGCACATATGGCTAAGTGGTGCAAATGAG :  949
BjTT8A-y_g : AACAAATCAAAATATAATGTGGAAGTATTAATTTGTAGGATGCCAGGAAAAGCGTATGCGAGGAGGAAGCACATATGGCTAAGTGGTGCAAATGAG :  949
BjTT8A-y_r : AACA-----------AATGTGGAAGTATTAATTTGTAGGATGCCAGGAAAAGCGTATGCGAGGAGGAAGCACATATGGCTAAGTGGTGCAAATGAG :  949
             AACAaatcaaaatatAATGTGGAAGTATTAATTTGTAGGATGCCAGGAAAAGCGTATGCGAGGAGGAAGCACATATGGCTAaGTGGTGCAAATGAG

                                       1000                                                      *
BrTT8A-b_g : GTTGACAATAAAATCTTCTCTAGGGCTATTTCTGCAAAGGTTCACATCTTCTA--CATTCACCACTACACTGTGCATCTACTTCTACTTATTTAGA : 1043
BrTT8A-y_g : GTTGACAATAAAATCTTCTCTAGGGCTATTTCTGCAAAGGTTCACATCTTCTATTCATTCACCACTACACTGTGCATCTACTTCTACTTATTTAGA : 1045
BnTT8A-b_g : GTTGACAATAAAATCTTCTCTAGGGCTATTTCTGCAAAGGTTCACATCTTCTA--CATTCACCACTACACTGTGCATCTACTTCTACTTATTTAGA : 1043
BnTT8A-y_g : GTTGACAATAAAATCTTCTCTAGGGCTATTTCTGCAAAGGTTCACATCTTCTA--CATTCACCACTACACTGTGCATCTACTTCTACTTATTTAGA : 1043
BjTT8A-d_g : GTTGACAATAAAATCTTCTCTAGGGCTATTTCTGCAAAGGTTCACATCTTCTA--CATTCACCACTACACTGTGCATCTACTTCTACTTATTTAGA : 1043
BjTT8A-y_g : GTTGACAATAAAATCTTCTCTAGGGCTATTTCTGCAAAGGTTCACATCTTCTA--CATTCACCACTACACTGTGCATCTACTTCTACTTATTTAGA : 1043
BjTT8A-y_r : GTTGACAATAAAATCTTCTCTAGGGCTATTTCTGCAAAGGTTCACATCTTCTA--CATTCACCACTACACTGTGCATCTACTTCTACTTATTTAGA : 1043
             GTTGACAATAAAATCTTCTCTAGGGCTATTTCTGCAAAGGTTCACATCTTCTA  CATTCACCACTACACTGTGCATCTACTTCTACTTATTTAGA

                                       1100                                                 extron4 *
BrTT8A-b_g : TTTATGCAATTTTATATATCTCATTCTGCAAACTAATTAATTTTATCTTCTTCTTTACTTGAATG--CTCTTTCCACATTAGAGTGCCAAAATTCA : 1137
BrTT8A-y_g : TATATGCAATTTTATATATCTCATTCTGCAAACTAATTAATTTTATCTTCTTCTTTACTTGAATGCTCTCTTTCCACATTAGAGTGCCAAAATTCA : 1141
BnTT8A-b_g : TTTATGCAATTTTATATATCTCATTCTGCAAACTAATTAATTTTATCTTCTTCTTTACTTGAATG--CTCTTTCCACATTAGAGTGCCAAAATTCA : 1137
BnTT8A-y_g : TTTATGCAATTTTATATATCTCATTCTGCAAACTAATTAATTTTATCTTCTTCTTTACTTGAATG--CTCTTTCCACATTAGAGTGCCAAAATTCA : 1137
BjTT8A-d_g : TTTATGCAATTTTATATATCTCATTCTGCAAACTAATTAATTTTATCTTCTTCTTTACTTGAATGCTCTCTTTCCACATTAGAGTGCCAAAATTCA : 1139
BjTT8A-y_g : TTTATGCAATTTTATATATCTCATTCTGCAAACTAATTAATTTTATCTTCTTCTTTACTTGAATGCTCTCTTTCCACATTAGAGTGCCAAAATTCA : 1139
BjTT8A-y_r : TTTATGCAATTTTATATATCTCATTCTGCAAACTAATTAATTTTATCTTCTTCTTTACTTGAATGCTCTCTTTCCACATTAGAGTGCCAAAATTCA : 1139
             TtTATGCAATTTTATATATCTCATTCTGCAAACTAATTAATTTTATCTTCTTCTTTACTTGAATG  CTCTTTCCACATTAGAGTGCCAAAATTCA

                                               1200
BrTT8A-b_g : GGTAAATTTTGCTTTATTAATTAATTATTTAATTTTCGTAGAAATGAAAGGTATCAATTAATAAAGTTATTTTACATATTTGACAATTGTTTTGTG : 1233
BrTT8A-y_g : GGTAAATTTTGCTTTATTAATTAATTATTTAATTTTCGTAGAAATGAAAGGTATCAATTAATAAAGTTATCTTACATATTTGACAATTGTTTTGTG : 1237
BnTT8A-b_g : GGTAAATTTTGCTTTATTAATTAATTATTTAATTTTCGTAGAAATGAAAGGTATCAATTAATAAAGTTATTTTACATATTTGACAATTGTTTTGTG : 1233
BnTT8A-y_g : GGTAAATTTTGCTTTATTAATTAATTATTTAATTTTCGTAGAAATGAAAGGTATCAATTAATAAAGTTATTTTACATATTTGACAATTGTTTTGTG : 1233
BjTT8A-d_g : GGTAAATTTTGCTTTATTAATTAATTATTTAATTTTCGTAGAAATGAAAGGTATCAATTAATAAAGTTATTTTACATATTTGACAATTGTTTTGTG : 1235
BjTT8A-y_g : GGTAAATTTTGCTTTATTAATTAATTATTTAATTTTCGTAGAAATGAAAGGTATCAATTAATAAAGTTATTTTACATATTTGACAATTGTTTTGTG : 1235
BjTT8A-y_r : GGTAAATTTTGCTTTATTAATTAATTATTTAATTTTCGTAGAAATGAAAGGTATCAATTAATAAAGTTATTTTACATATTTGACAATTGTTTTGTG : 1235
             GGTAAATTTTGCTTTATTAATTAATTATTTAATTTTCGTAGAAATGAAAGGTATCAATTAATAAAGTTATtTTACATATTTGACAATTGTTTTGTG

               *                     extron5          1300
BrTT8A-b_g : ATGAAAAAAA-AAAAAT------AATCAGACAGTGGTTTGCATTCCCGTGCTTGATGGCGTTTTGGAACTAGGCACAACGAACAAGGTGAAAATCT : 1322
BrTT8A-y_g : ATGAAAAAAATAATAATGAAAAAAATCAGACAGTGGTTTGCATTCCCGTGCTTGATGGCGTTTTGGAACTAGGCACAACGAACAAGGTAAAAATCT : 1333
BnTT8A-b_g : ATGAAAAAA--AATAAT------AATCAGACAGTGGTTTGCATTCCCGTGCTTGATGGCGTTTTGGAACTAGGCACAACGAACAAGGTAAAAATCT : 1321
BnTT8A-y_g : ATGAAAAAA--AATAAT------AATCAGACAGTGGTTTGCATTCCCGTGCTTGATGGCGTTTTGGAACTAGGCACAACGAACAAGGTAAAAATCT : 1321
BjTT8A-d_g : ATGAAAAAAATAATAAT------AATCAGACAGTGGTTTGCATTCCCGTGCTTGATGGCGTTTTGGAACTAGGCACAACGAACAAGGTAAAAATCT : 1325
BjTT8A-y_g : ATGAAAAAAATAATAAT------AATCAGACAGTGGTTTGCATTCCCGTGCTTGATGGCGTTTTGGAACTAGGCACAACGAACAAGGTAAAAATCT : 1325
BjTT8A-y_r : ATGAAAAAAATAATAAT------AATCAGACAGTGGTTTGCATTCCCGTGCTTGATGGCGTTTTGGAACTAGGCACAACGAACAAGGTAAAAATCT : 1325
             ATGAAAAAA  AAtAAT      AATCAGACAGTGGTTTGCATTCCCGTGCTTGATGGCGTTTTGGAACTAGGCACAACGAACAAGGTaAAAATCT

                   *                                      1400
BrTT8A-b_g : CTATTTCTGTCGGTACCCAAAATGTAGTCGAATATATTCAGCTCATTCTATATGTTACTTAGAGTATCTCCAAAAAGACTATATATGGTAAAGTTT : 1418
BrTT8A-y_g : CTATTTATGTCGGTACCCAAAATGTAGTCGAATATATTCAGCTCATTCTATATGTTACTTAGATCATCTCCAAAAAGACTATATATGGTAAAGTTT : 1429
BnTT8A-b_g : CTATTTCTGTCGGTACCCAAAATGTAGTCGAATATATTCAGCTCATTCTATATGTTACTTAGAGTATCTCTAAAAAGACTATATATGGTAAAGTTT : 1417
BnTT8A-y_g : CTATTTCTGTCGGTACCCAAAATGTAGTCGAATATATTCAGCTCATTCTATATGTTACTTAGAGTATCTCTAAAAAGACTATATATGGTAAAGTTT : 1417
BjTT8A-d_g : CTATTTCTGTCGGTACCCAAAATATAGTCGAATATATTCAGCTCATTCTATATGTTACTTTGAGTATCTCCAAAAAGACTATATATGGTAAAGTTT : 1421
BjTT8A-y_g : CTATTTCTGTCGGTACCCAAAATATAGTCGAATATATTCAGCTCATTCTATATGTTACTTTGAGTATCTCCAAAAAGACTATATATGGTAAAGTTT : 1421
BjTT8A-y_r : CTATTTCTGTCGGTACCCAAAATATAGTCGAATATATTCAGCTCATTCTATATGTTACTTTGAGTATCTCCAAAAAGACTATATATGGTAAAGTTT : 1421
             CTATTTcTGTCGGTACCCAAAAT TAGTCGAATATATTCAGCTCATTCTATATGTTACTT GAgtATCTC AAAAAGACTATATATGGTAAAGTTT

                       *                                      1500
BrTT8A-b_g : CCAAAATTCTATATTCGAAGTTTCAAGGTATTTTTTTTTTCCAAAACAAAATTTCAAATTTAACTTCAAAATTATTTGTAATTTACAATATGATCCT : 1514
BrTT8A-y_g : CCAAAATTCTATATTCAAAGTTTCAAAGTG-CTTTTTCTCCAAAAACAAAATTTTAAATTTAACTTCAAAATTATTTGTAATTTACAGTATGATCCT : 1524
BnTT8A-b_g : TCAAAATTCTATATTCGAAGTTTCAAGGTATTTTTTTTTTCAAAAACAAAATTTCAAATTTAACTTCAAAATTATTTGTAATTTACAATATGATCCT : 1513
BnTT8A-y_g : TCAAAATTCTATATTCGAAGTTTCAAGGTATTTTTTTTTTCAAAAACAAAATTTCAAATTTAACTTCAAAATTATTTGTAATTTACAATATGATCCT : 1513
BjTT8A-d_g : CCAAAATTCTATATTCGAAGTTTTAAGGTATTTTTTTTTTCAAAAACAAAATTTCAAATTTAACTTTAAAATTATTTGTAATTTACAATATGATCCT : 1517
BjTT8A-y_g : CCAAAATTCTATATTCGAAGTTTTAAGGTATTTTTTTTTTCAAAAACAAAATTTCAAATTTAACTTTAAAATTATTTGTAATTTACAATATGATCCT : 1517
BjTT8A-y_r : CCAAAATTCTATATTCGAAGTTTTAAGGTATTTTTTTTTTCAAAAACAAAATTTCAAATTTAACTTTAAAATTATTTGTAATTTACAATATGATCCT : 1517
              CAAAATTCTATATTCgAAGTTT AAgGTattTTTTTtTtCaAAAACAAAATTTcAAATTTAACTT AAAATTATTTGTAATTTACAaTATGATCCT

                           *                                      1600
BrTT8A-b_g : TATACTTATCATAATTAATATAAATACATAAAACTTTTATAAATAACTAGCACATATAAAAAAATATTACAATAATATTAATTAATAAATTCTTACA : 1610
BrTT8A-y_g : TATACTTATCATAATTAATATAAATACATAAAACTTTTATAAATAACTAGCACATATAAAAAAATATTATAATAATATTAATTAATAAATTCTTACA : 1620
BnTT8A-b_g : TATACTTATCATAATTAATATAAATACATAAAACTTTTATAAATAACTAGCACATATAAAAAAATATTACAATAATATTAATTAATAAATTCTTACA : 1609
BnTT8A-y_g : TATACTTATCATAATTAATATAAATACATAAAACTTTTATAAATAACTAGCACATATAAAAAAATATTACAATAATATTAATTAATAAATTCTTACA : 1609
BjTT8A-d_g : TATACTTATCATAATTAATATAAATACATAAAACTTTTATAAATAACTAGCACATATAAAAAAATATTACAATAATATTAATTAATAAATTCTTACA : 1613
BjTT8A-y_g : TATACTTATCATAATTAATATAAATACATAAAACTTTTATAAATAACTAGCACATATAAAAAAATATTACAATAATATTAATTAATAAATTCTTACA : 1613
BjTT8A-y_r : TATACTTATCATAATTAATATAAATACATAAAACTTTTATAAATAACTAGCACATATAAAAAAATATTACAATAATATTAATTAATAAATTCTTACA : 1613
             TATACTTATCATAATTAATATAAATACATAAAACTTTTATAAATAACTAGCACATATAAAAAAATATTAcAATAATATTAATTAATAAATTCTTACA

                           *                                      1700
BrTT8A-b_g : CTAAAATATAAAATTATTAACAAAAATACATAATTAAATATT-AAATACAAGCAAAATATCACATTAGTCAATAAAATTATTTCTGCAATGCTCCA : 1705
BrTT8A-y_g : CTAAAATATAAAATTATTAACAAAAATACATAATTAAATATTAAAATACAAGCAAAATATCACATTAGTCAATAAAATTATTTCTGCAATGCTCCA : 1716
BnTT8A-b_g : CTAAAATATAAAATTATTAACAAAAATACATAATTAAATATT-AAATACAAGCAAAATATCACATTAGTCAATAAAATTATTTCTGCAATGCTCCA : 1704
BnTT8A-y_g : CTAAAATATAAAATTATTAACAAAAATACATAATTAAATATT-AAATACAAGCAAAATATCACATTAGTCAATAAAATTATTTCTGCAATGCTCCA : 1704
BjTT8A-d_g : CTAAAATATAAAATTATTAACAAAAATACATAATTAAATATT-AAATACAAGCAAAATATCACATTAGTCAATAAAATTATTTCTGCAATGCTCCA : 1708
BjTT8A-y_g : CTAAAATATAAAATTATTAACAAAAATACATAATTAAATATT-AAATACAAGCAAAATATCACATTAGTCAATAAAATTATTTCTGCAATGCTCCA : 1708
BjTT8A-y_r : CTAAAATATAAAATTATTAACAAAAATACATAATTAAATATT-AAATACAAGCAAAATATCACATTAGTCAATAAAATTATTTCTGCAATGCTCCA : 1708
             CTAAAATATAAAATTATTAACAAAAATACATAATTAAATATT AAATACAAGCAAAATATCACATTAGTCAATAAAATTATTTCTGCAATGCTCCA
```

```
                                        *                                                  1800
BrTT8A-b_g : TCTTCGGTTACACAAAATTTGTTTGGAAAATATTCTCGAGCTTCTGGAGGAAATTTACTAGACTATTAGTGTTATTGTAATATTTAAATTTGTGCA : 1801
BrTT8A-y_g : TCTTCGGTTACACAAAATTTGTTTGAAAAATATTCTAGAGCTTCTGGAGAAAATTTACTAGACTATTAGTGTTATTGTAATATTTAAATTTGTGCA : 1812
BnTT8A-b_g : TCTTCGGTTACACAAAATTTGTTTGGAAAATATTCTCGAGCTTCTGGAGCAAATTTACTAGACTATTAGTGTTATTGTAATATTTAAATTTATGCA : 1800
BnTT8A-y_g : TCTTCGGTTACACAAAATTTGTTTGGAAAATATTCTCGAGCTTCTGGAGCAAATTTACTAGACTATTAGTGTTATTGTAATATTTAAATTTATGCA : 1800
BjTT8A-d_g : TTTTCGGTTACACAAAATTTGTTTGGAAAATATTTTGGAGCTTCTGGAGCAAATTTACTAGACTATTAGTGTTATTGTAATATTTAAATTTGTGCA : 1804
BjTT8A-y_g : TTTTCGGTTACACAAAATTTGTTTGGAAAATATTTTGGAGCTTCTGGAGCAAATTTACTAGACTATTAGTGTTATTGTAATATTTAAATTTGTGCA : 1804
BjTT8A-y_r : TTTTCGGTTACACAAAATTTGTTTGGAAAATATTTTGGAGCTTCTGGAGCAAATTTACTAGACTATTAGTGTTATTGTAATATTTAAATTTGTGCA : 1804
             T TTCGGTTACACAAAATTTGTTTGgAAAATATT T GAGCTTCTGGAG AAATTTACTAGACTATTAGTGTTATTGTAATATTTAAATTT TGCA

                                        *                                                  1900
BrTT8A-b_g : ATAATTATGTCTTCATGTATTTTTTAAAAAATGTTT-TTTATTAAG---TTTTTTTGTAATATTCTTTTTGTGTAATTTTAGTTATAAAATATTAT : 1893
BrTT8A-y_g : ATAACTATGTCTTCATGTATTTTTTTAAAAATGTTTATTTATTGAGTTTTTTTTTTGTAATA-TCTTGTTGTGTAATTTTAGTTATAAAATATTAT : 1907
BnTT8A-b_g : ATAATTATGTCTTCATGTATTTTTTTAAAAATGTTT-TTTATTAAG--TTTTTTTTGTAATATTCTTTTTGTGTAATTTTAGTTATAAAATATTAT : 1893
BnTT8A-y_g : ATAATTATGTCTTCATGTATTTTTTTAAAAATGTTT-TTTATTAAG--TTTTTTTTGTAATATTCTTTTTGTGTAATTTTAGTTATAAAATATTAT : 1893
BjTT8A-d_g : ATAATTATGTCTTCATGTATTTTTT-AAAAATGTTT-TTTATTAAG--TTTTTTTTGTAATATTCTTTTTGTGTAATTTTAGTTATAAAATATTAT : 1896
BjTT8A-y_g : ATAATTATGTCTTCATGTATTTTTT-AAAAATGTTT-TTTATTAAG--TTTTTTTTGTAATATTCTTTTTGTGTAATTTTAGTTATAAAATATTAT : 1896
BjTT8A-y_r : ATAATTATGTCTTCATGTATTTTTT-AAAAATGTTT-TTTATTAAG--TTTTTTTTGTAATATTCTTTTTGTGTAATTTTAGTTATAAAATATTAT : 1896
             ATAAtTATGTCTTCATGTATTTTTT AAAAATGTTT TTTATTaAG  tTTTTTTTGTAATAtTCTTtTTGTGTAATTTTAGTTATAAAATATTAT

                                        *                                                  2000
BrTT8A-b_g : AAATCTTAACTTAAAATTTTTGTTTAATTTTATGCGTAAATTTTGAATTTAAAAAGTAATTTTGAAATATTTATGATATAAAAATGTTTTAAAGAT : 1989
BrTT8A-y_g : AAATCTTAACTTAAAATTTTTATTTAATTTTATGTGTAAATTTTGAATTTAAAAAGTAATTTTGAAATATTTATGAAATAAAAATGTCTTAAAGAT : 2003
BnTT8A-b_g : AAATCTTAACTTAAATTTTTTTTTTAATTTTATGCGTAAATTTTGAATTTAAAAAGTAATTTTGAAATATTTATGATATAAAAATGTTTTAAAGAT : 1989
BnTT8A-y_g : AAATCTTAACTTAAATTTTTTTTTTAATTTTATGCGTAAATTTTGAATTTAAAAAGTAATTTTGAAATATTTATGATATAAAAATGTTTTAAAGAT : 1989
BjTT8A-d_g : AAATCTTAACTTAAAATTTTAGTTTAATTTTATGCGTAAATTTTGAATTTAAAAAGTAATTTTGAAATATTTATGATATAAAAATGTTTTAAAGAT : 1992
BjTT8A-y_g : AAATCTTAACTTAAAATTTTAGTTTAATTTTATGCGTAAATTTTGAATTTAAAAAGTAATTTTGAAATATTTATGATATAAAAATGTTTTAAAGAT : 1992
BjTT8A-y_r : AAATCTTAACTTAAAATTTTAGTTTAATTTTATGCGTAAATTTTGAATTTAAAAAGTAATTTTGAAATATTTATGATATAAAAATGTTTTAAAGAT : 1992
             AAATCTTAACTTAAA TTTT  TTTAATTTTATGcGTAAATTTTGAATTTAAAAAGTAATTTTGAAATATTTATGAtATAAAAATGTtTTAAAGAT

                                        *                                                  2100
BrTT8A-b_g : TAATAAGATAAATGAGAAAATATTTAAAAATCATTAATATAATGTGTAATTAATTATAAGAACCAAAATACAAATAAAAAGAAAAAATTCTAAATT : 2085
BrTT8A-y_g : TAATAAGATAAATGAGAAAACATTTAAAAATTATTAATATAATGTGTAATTAAT--TAAAGACCAAAATACAAATAAAAAGAAGAAATTCCAAATT : 2097
BnTT8A-b_g : TAATAAGATAAATGATAAAATATTTAAAAATCATTAATATAATGTGTAATTAATTATAAGGACCAAAATACAAATAAAAAGAAGAAATTCTAAATT : 2085
BnTT8A-y_g : TAATAAGATAAATGATAAAATATTTAAAAATCATTAATATAATGTGTAATTAATTATAAGGACCAAAATACAAATAAAAAGAAGAAATTCTAAATT : 2085
BjTT8A-d_g : TAATAAGATAAATGAGAAAATATTTAAAAATCATTAATATAATGTGTAATTAATTATAAGGACCAAAATACAAATAAAAAGAAAAAATTCTAAATT : 2088
BjTT8A-y_g : TAATAAGATAAATGAGAAAATATTTAAAAATCATTAATATAATGTGTAATTAATTATAAGGACCAAAATACAAATAAAAAGAAAAAATTCTAAATT : 2088
BjTT8A-y_r : TAATAAGATAAATGAGAAAATATTTAAAAATCATTAATATAATGTGTAATTAATTATAAGGACCAAAATACAAATAAAAAGAAAAAATTCTAAATT : 2088
             TAATAAGATAAATGA AAAAtATTTAAAAATcATTAATATAATGTGTAATTAATtaTAAggACCAAAATACAAATAAAAAGAA AAATTCtAAATT

                                        *                                                  2200
BrTT8A-b_g : TGGAGTTTTGAGTAGTGAACTTCAAATATGAAGTTTCATTCTTTAAAACTCTAAATTCTAAGTTTGAAGTTTTAAAGTTATTTTTTGGAGAGAAAA : 2181
BrTT8A-y_g : TGGAGTTTTGAGTAATGAACTTCAAATATGAAGTTTCATTCTTTAAAACTCTAAATTCTAAGTTTGAAGTTTTGAAGTTATTTTTTGGAGAG-AAA : 2192
BnTT8A-b_g : TGGAGTTTTGAGTAGTGAACTTCAAATATGAAGTTTCATTTTTTAAAACTCTAAATTCTAAGTTTGAAGTTTTAAAGTTATTTTTTGGAGAG-AAA : 2180
BnTT8A-y_g : TGGAGTTTTGAGTAGTGAACTTCAAATATGAAGTTTCATTTTTTAAAACTCTAAATTCTAAGTTTGAAGTTTTAAAGTTATTTTTTGGAGAG-AAA : 2180
BjTT8A-d_g : TGGAGTTTTGAGTAGTGAACTTCAAATATGAAGTTTCATTCTTTAAAACTCAAAATTCTAAGTTTGAAGTTTTAAAGTTATATTTTGGAGAG-AAA : 2183
BjTT8A-y_g : TGGAGTTTTGAGTAGTGAACTTCAAATATGAAGTTTCATTCTTTAAAACTCAAAATTCTAAGTTTGAAGTTTTAAAGTTATATTTTGGAGAG-AAA : 2183
BjTT8A-y_r : TGGAGTTTTGAGTAGTGAACTTCAAATATGAAGTTTCATTCTTTAAAACTCAAAATTCTAAGTTTGAAGTTTTAAAGTTATATTTTGGAGAG-AAA : 2183
             TGGAGTTTTGAGTAgTGAACTTCAAATATGAAGTTTCATT TTTAAAACTC AAATTCTAAGTTTGAAGTTTTaAAGTTAT TTTTGGAGAG AAA

                                        *                                                  2300
BrTT8A-b_g : AAACTTTATATTTGAAGTCATAGAGTTTCATTTGGAGATGTTATTAGAGAAAAAAAA---TATATATATATACAGAAACATCAACCCTTCGCAGTC : 2274
BrTT8A-y_g : AAACTCTATATTTGAAGTTATAGAGTTTCATTTGGAGATATTCTTAGAGAAATAAAA----AAAAATATATACAGAAACATCAACCATTCGCAGTC : 2284
BnTT8A-b_g : AAACTTTATATTTGAAGTCATAGAGTTTCATTTGGAGATGCTATTAGAGAAAAAAAAATATATATATATATACAGAAACATCAACCCTTCGCAGTC : 2276
BnTT8A-y_g : AAACTTTATATTTGAAGTCATAGAGTTTCATTTGGAGATGCTATTAGAGAAAAAAAAATATATATATATATACAGAAACATCAACCCTTCGCAGTC : 2276
BjTT8A-d_g : AAACTTTATATTTGAAGTCATAGAGTTTCATTTGGAGATGTTATTAGAGAAAAAAAA----ATATATATATACAGAAACATCAACCCTTCGCAGTC : 2275
BjTT8A-y_g : AAACTTTATATTTGAAGTCATAGAGTTTCATTTGGAGATGTTATTAGAGAAAAAAAA----ATATATATATACAGAAACATCAACCCTTCGCAGTC : 2275
BjTT8A-y_r : AAACTTTATATTTGAAGTCATAGAGTTTCATTTGGAGATGTTATTAGAGAAAAAAAA----ATATATATATACAGAAACATCAACCCTTCGCAGTC : 2275
             AAACTtTATATTTGAAGTcATAGAGTTTCATTTGGAGATg TaTTAGAGAAAaAAAA    AtAtATATATACAGAAACATCAACCcTTCGCAGTC

                                        *                                                  2400
BrTT8A-b_g : ATATGAT-AAAAAAATATAGTTTTCGATTACATTAAATACACAACCAAATTATGTAAAACTATACAACCTAATGAAAATATGCATAAGCGGGAGAAC : 2369
BrTT8A-y_g : ATATGAT-AAAAAAATATAGTTTTCGATTACATTAAATACACAACCAAATTATGTAAAACTAAACAACCTAATGAAAATATGCATAAGCGGGAGAAC : 2379
BnTT8A-b_g : ATATGAT-AAAAAAATATAGTTTTCGATTACATTAAATACACAACCAAATTATGTAAAACTATACAACCTAATGAAAATATGCATAAGCGGGAGAAC : 2371
BnTT8A-y_g : ATATGAT-AAAAAAATATAGTTTTCGATTACATTAAATACACAACCAAATTATGTAAAACTATACAACCTAATGAAAATATGCATAAGCGGGAGAAC : 2371
BjTT8A-d_g : ATATGATAAAAAAAATATAGTTTTCGATTACATTAAATAGACAACCAAATTATGTAAAACTATACAACCTAATGAAAATATGCATAAGCGGGAGAAC : 2371
BjTT8A-y_g : ATATGATAAAAAAAATATAGTTTTCGATTACATTAAATAGACAACCAAATTATGTAAAACTATACAACCTAATGAAAATATGCATAAGCGGGAGAAC : 2371
BjTT8A-y_r : ATATGATAAAAAAAATATAGTTTTCGATTACATTAAATAGACAACCAAATTATGTAAAACTATACAACCTAATGAAAATATGCATAAGCGGGAGAAC : 2371
             ATATGAT AAAAAAATATAGTTTTCGATTACATTAAATA ACAACCAAATTATGTAAAACTAtACAACCTAATGAAAATATGCATAAGCGGGAGAAC
```

**extron6**

```
                                                 *
BrTT8A-b_g : CAGGGAGATGGATGCATGATATATTGTGTTTGTATGTGAAGGTCAAAGAGAGTGAAGAGTTTGTTGAGCACATAAAGAGTTTCTTCCACAACCACC : 2465
BrTT8A-y_g : CAGGGAGATGAATGTATGATATATTGTGTTTGTATGTGAAGGTCAAAGAGAGTGAAGAGTTTGTTGAGCACATAAAGAGTTTCTTCCACAACCACC : 2475
BnTT8A-b_g : CAGGGAGATGGATGCATGATATATTGTGTTTGTATGTGAAGGTCAAAGAGAGTGAAGAGTTTGTTGAGCACATAAAGAGTTTCTTCCACAACCACC : 2467
BnTT8A-y_g : CAGGGAGATGGATGCATGATATATTGTGTTTGTATGTGAAGGTCAAAGAGAGTGAAGAGTTTGTTGAGCACATAAAGAGTTTCTTCCACAACCACC : 2467
BjTT8A-d_g : CAGGGAGATGGATGCATGATATATTGTGTTTGTATGTGAAGGTCAAAGAGAGTGAAGAGTTTGTTGAGCACATAAAGAGTTTCTTCCACAACCACC : 2467
BjTT8A-y_g : CAGGGAGATGGATGCATGATATATTGTGTTTGTATGTGAAGGTCAAAGAGAGTGAAGAGTTTGTTGAGCACATAAAGAGTTTCTTCCACAACCACC : 2467
BjTT8A-y_r : CAGGGAGATGGATGCATGATATATTGTGTTTGTATGTGAAGGTCAAAGAGAGTGAAGAGTTTGTTGAGCACATAAAGAGTTTCTTCCACAACCACC : 2467
             CAGGGAGATGgATGcATGATATATTGTGTTTGTATGTGAAGGTCAAAGAGAGTGAAGAGTTTGTTGAGCACATAAAGAGTTTCTTCCACAACCACC

             2500                                         *
BrTT8A-b_g : CGAAGTCAAACACTAAGCCTACTCTTTCTGAACACTTCATCAACGAAGAGCATGAAGAAGACGAAGAAGAAGTAGAAGAAGAAGAAATGACAATGT : 2561
BrTT8A-y_g : CGAAGTCAAACATTAAGCCTACTCTTTCTGAACACTTCATCAACGAAGAGCATGAAGAAGACGAAGAAGAAGTAGAAGAAGAAGAAATGACAATGT : 2571
BnTT8A-b_g : CGAAGTCAAACACTAAGCCTACTCTTTCTGAACACTTCATCAACGAAGAGCATGAAGAAGACGAAGAAGAAGTAGAAGAAGAAGAAATGACAATGT : 2563
BnTT8A-y_g : CGAAGTCAAACACTAAGCCTACTCTTTCTGAACACTTCATCAACGAAGAGCATGAAGAAGACGAAGAAGAAGTAGAAGAAGAAGAAATGACAATGT : 2563
BjTT8A-d_g : CGAAGTCAAACACTAAGCCTACTCTTTCTGAACACTTCATCAACGAAGAGCATGAAGAAGACGAAGAAGAAGTAGAAGAAGAAGAAATGACAATGT : 2563
BjTT8A-y_g : CGAAGTCAAACACTAAGCCTACTCTTTCTGAACACTTCATCAACGAAGAGCATGAAGAAGACGAAGAAGAAGTAGAAGAAGAAGAAATGACAATGT : 2563
BjTT8A-y_r : CGAAGTCAAACACTAAGCCTACTCTTTCTGAACACTTCATCAACGAAGAGCATGAAGAAGACGAAGAAGAAGTAGAAGAAGAAGAAATGACAATGT : 2563
             CGAAGTCAAACAcTAAGCCTACTCTTTCTGAACACTTCATCAACGAAGAGCATGAAGAAGACGAAGAAGAAGTAGAAGAAGAAGAAATGACAATGT
```

```
            2600                                                            *
BrTT8A-b_g : CAGAAGAGATAAGACTTGGTTCTCCTGATGACGATGACGTCTCCAATCAAAATCTACTCTCTGATTTCCATATAGAAGCAACCAATAGTTTAGGTA : 2657
BrTT8A-y_g : CAGAAGAGATAAGACTTGGTTCTCCTGATGACGATGACGTCTCCAATCAAAATCTACTCTCTGATTTCCATATAGAAGCAACCAATAGTTTAGGTA : 2667
BnTT8A-b_g : CAGAAGAGATAAGACTTGGTTCTCCTGATGACGATGACGTCTCCAATCAAAATCTACTCTCTGATTTCCATATAGAAGCAACCAATAGTTTAGGTA : 2659
BnTT8A-y_g : CAGAAGAGATAAGACTTGGTTCTCCTGATGACGATGACGTCTCCAATCAAAATCTACTCTCTGATTTCCATATAGAAGCAACCAATAGTTTAGGTA : 2659
BjTT8A-d_g : CAGAAGAGATAAGACTTGGTTCTCCTGATGACGATGACGTCTCCAATCAAAATCTACTCTCTGATTTCCATATAGAAGCAACCAATAGTTTAGGTA : 2659
BjTT8A-y_g : CAGAAGAGATAAGACTTGGTTCTCCTGATGACGATGACGTCTCCAATCAAAATCTACTCTCTGATTTCCATATAGAAGCAACCAATAGTTTAGGTA : 2659
BjTT8A-y_r : CAGAAGAGATAAGACTTGGTTCTCCTGATGACGATGACGTCTCCAATCAAAATCTACTCTCTGATTTCCATATAGAAGCAACCAATAGTTTAGGTA : 2659
             CAGAAGAGATAAGACTTGGTTCTCCTGATGACGATGACGTCTCCAATCAAAATCTACTCTCTGATTTCCATATAGAAGCAACCAATAGTTTAGGTA

                2700                                                            *
BrTT8A-b_g : TACCGTACACACCTTTCTTATTACATTAAATTAGTTAACAATATCATTATAATTAATTTTCTAATAATAAATTTTTTAAACTGGTTCTCATGTCTG : 2753
BrTT8A-y_g : TACCGTACACACCTCTCTTATTACATTAAATTAGTTAACAATATCATTATAATTAATTTTCTAATAATAAATTTTTTAAACTGGTTCTCATGTCTG : 2763
BnTT8A-b_g : TACCGTACACACCTTTCTTATTACATTAAATTAGTTAACAATATCATTATAATTAATTTTCTAATAATAAATTTTTTAAACTGGTTCTCATGTCTG : 2755
BnTT8A-y_g : TACCGTACACACCTTTCTTATTACATTAAATTAGTTAACAATATCATTATAATTAATTTTCTAATAATAAATTTTTTAAACTGGTTCTCATGTCTG : 2755
BjTT8A-d_g : TACCGTACACACCTTTCTTATTACATTAAATTAGTTAACAATATCATTATAATTAATTTTCTAATAATAAATTTTTTAAACTGGTTCTCATGTCTG : 2755
BjTT8A-y_g : TACCGTACACACCTTTCTTATTACATTAAATTAGTTAACAATATCATTATAATTAATTTTCTAATAATAAATTTTTTAAACTGGTTCTCATGTCTG : 2755
BjTT8A-y_r : TACCGTACACACCTTTCTTATTACATTAAATTAGTTAACAATATCATTATAATTAATTTTCTAATAATAAATTTTTTAAACTGGTTCTCATGTCTG : 2755
             TACCGTACACACCTtTCTTATTACATTAAATTAGTTAACAATATCATTATAATTAATTTTCTAATAATAAATTTTTTAAACTGGTTCTCATGTCTG

                    2800       extron7                                        *
BrTT8A-b_g : GTAATTCTAACATCTATCATTATATAAATAGATACACACATGGACATGATGAATCTAATGGAGGAAGGAGGAAATTATTCTCAGACAGTATCAACA : 2849
BrTT8A-y_g : GTAATTCTAACATCTATCATTGTATAAATAGATACACACATGGACATGATGAATCTAATGGAGGAAGGCGGAAATTATTCTCAGACAGTATCAACA : 2859
BnTT8A-b_g : GTAATTCTAACATCTATCATTGTATAAATAGATACACACATGGACATGATGAATCTAATGGAGGAAGGCGGAAATTATTCTCAGACAGTATCAACA : 2851
BnTT8A-y_g : GTAATTCTAACATCTATCATTGTATAAATAGATACACACATGGACATGATGAATCTAATGGAGGAAGGCGGAAATTATTCTCAGACAGTATCAACA : 2851
BjTT8A-d_g : GTAATTCTAACATCTATCATTGTATAAATAGATACACACATGGACATGATGAATCTAATGGAGGAAGGAGGAAATTATTCTCAGACAGTATCAACA : 2851
BjTT8A-y_g : GTAATTCTAACATCTATCATTGTATAAATAGATACACACATGGACATGATGAATCTAATGGAGGAAGGAGGAAATTATTCTCAGACAGTATCAACA : 2851
BjTT8A-y_r : GTAATTCTAACATCTATCATTGTATAAATAGATACACACATGGACATGATGAATCTAATGGAGGAAGGAGGAAATTATTCTCAGACAGTATCAACA : 2851
             GTAATTCTAACATCTATCATTgTATAAATAGATACACACATGGACATGATGAATCTAATGGAGGAAGG GGAAATTATTCTCAGACAGTATCAACA

                        2900                                                            *
BrTT8A-b_g : CTTCTCATGTCACAACCCACAAGTCTTCTTTCAGATTCAGTTTCCACATCTTCTTACGTTCAATCATCGTTTGTCTCGTGGAGAGTTGAGAATGTC : 2945
BrTT8A-y_g : CTTCTCATGTCACAACCCACAAGTCTTCTTTCAGATTCAGTTTCCACATCTTCTTACGTTCAATCATCGTTTGTCTCGTGGAGAGTTGAGAATGTC : 2955
BnTT8A-b_g : CTTCTCATGTCACAACCCACAAGTCTTCTTTCAGATTCAGTTTCCACATCTTCTTACGTTCAATCATCGTTTGTCTCGTGGAGAGTTGAGAATGTC : 2947
BnTT8A-y_g : CTTCTCATGTCACAACCCACAAGTCTTCTTTCAGATTCAGTTTCCACATCTTCTTACGTTCAATCATCGTTTGTCTCGTGGAGAGTTGAGAATGTC : 2947
BjTT8A-d_g : CTTCTCATGTCACAACCCACAAGTCTTCTTTCAGAATCAGTTTCCACATCTTCTTACGTTCAATCATCGTTTGTCTCGTGGAGAGTTGAGAATGTC : 2947
BjTT8A-y_g : CTTCTCATGTCACAACCCACAAGTCTTCTTTCAGAATCAGTTTCCACATCTTCTTACGTTCAATCATCGTTTGTCTCGTGGAGAGTTGAGAATGTC : 2947
BjTT8A-y_r : CTTCTCATGTCACAACCCACAAGTCTTCTTTCAGAATCAGTTTCCACATCTTCTTACGTTCAATCATCGTTTGTCTCGTGGAGAGTTGAGAATGTC : 2947
             CTTCTCATGTCACAACCCACAAGTCTTCTTTCAGA TCAGTTTCCACATCTTCTTACGTTCAATCATCGTTTGTCTCGTGGAGAGTTGAGAATGTC

                            3000                                                            *
BrTT8A-b_g : AAAGAGCATCAGCAATATCAGCGAGTGGAGAAAGCGGCGTGGTCATCGTCGCAATGGATGCTCAAACACATAATCTTGAAAGTTCCTTTCCTCCAC : 3041
BrTT8A-y_g : AAAGAGCATCAGCAATATCAGCGAGTGGAGAAAGCGGCGTGGTCATCGTCGCAATGGATGCTCAAACACATAATCTTGAAAGTTCCTTTCCTCCAC : 3051
BnTT8A-b_g : AAAGAGCATCAGCAATATCAGCGAGTGGAGAAAGCGGCGTGGTCATCGTCGCAATGGATGCTCAAACACATAATCTTGAAAGTTCCTTTCCTCCAC : 3043
BnTT8A-y_g : AAAGAGCATCAGCAATATCAGCGAGTGGAGAAAGCGGCGTGGTCATCGTCGCAATGGATGCTCAAACACATAATCTTGAAAGTTCCTTTCCTCCAC : 3043
BjTT8A-d_g : AAAGAGCATCAGCAATATCAGCGAGTGGAGAAAGCGGCGTGGTCATCGTCGCAATGGATGCTCAAACACATAATCTTGAAAGTTCCTTTCCTCCAC : 3043
BjTT8A-y_g : AAAGAGCATCAGCAATATCAGCGAGTGGAGAAAGCGGCGTGGTCATCGTCGCAATGGATGCTCAAACACATAATCTTGAAAGTTCCTTTCCTCCAC : 3043
BjTT8A-y_r : AAAGAGCATCAGCAATATCAGCGAGTGGAGAAAGCGGCGTGGTCATCGTCGCAATGGATGCTCAAACACATAATCTTGAAAGTTCCTTTCCTCCAC : 3043
             AAAGAGCATCAGCAATATCAGCGAGTGGAGAAAGCGGCGTGGTCATCGTCGCAATGGATGCTCAAACACATAATCTTGAAAGTTCCTTTCCTCCAC

             ↓insertion strat 3100                                                               *
BrTT8A-b_g : G----------------------------------------------------------------------------------------------- : 3042
BrTT8A-y_g : G----------------------------------------------------------------------------------------------- : 3052
BnTT8A-b_g : G----------------------------------------------------------------------------------------------- : 3044
BnTT8A-y_g : G----------------------------------------------------------------------------------------------- : 3044
BjTT8A-d_g : G----------------------------------------------------------------------------------------------- : 3044
BjTT8A-y_g : GTCCCGTCGTCCCTCTTCTGTATCTTCTCCGGTGGCCGACGGTGGGCCTCGTCCCGTCGGTCGCCACCCCCACTTCGCCCCGCCGTATCCAATCCT : 3139
BjTT8A-y_r : GTCCCGTCGTCCCTCTTCTGTATCTTCTCCGGTGGCCGACGGTGGGCCTCGTCCCGTCGGTCGCCACCCCCACTTCGCCCCGCCGTATCCAATCCT : 3139
             G

                                        3200                                                    *
BrTT8A-b_g : ------------------------------------------------------------------------------------------------ :    -
BrTT8A-y_g : ------------------------------------------------------------------------------------------------ :    -
BnTT8A-b_g : ------------------------------------------------------------------------------------------------ :    -
BnTT8A-y_g : ------------------------------------------------------------------------------------------------ :    -
BjTT8A-d_g : ------------------------------------------------------------------------------------------------ :    -
BjTT8A-y_g : CTCCCTGTCAAGATACGGTCACTTCACCTCCTCTTTGCTTCTCCTTCTCTGGTTTTCACCGGATTGCGGCCGGATCCGGTGACCAAATCGATTTTT : 3235
BjTT8A-y_r : CTCCCTGTCAAGATACGGTCACTTCACCTCCTCTTTGCTTCTCCTTCTCTGGTTTTCACCGGATTGCGGCCGGATCCGGTGACCAAATCGATTTTT : 3235

                                            3300                                                    *
BrTT8A-b_g : ------------------------------------------------------------------------------------------------ :    -
BrTT8A-y_g : ------------------------------------------------------------------------------------------------ :    -
BnTT8A-b_g : ------------------------------------------------------------------------------------------------ :    -
BnTT8A-y_g : ------------------------------------------------------------------------------------------------ :    -
BjTT8A-d_g : ------------------------------------------------------------------------------------------------ :    -
BjTT8A-y_g : GGGTGTGTCCCCAATGTCTCTGGAGAGAGGGCGAGGTCGTGACTGTCCGGAAGCTTACAGGAGGCACCAATTGTTTTGTAGATCTAGGGTTTGTTT : 3331
BjTT8A-y_r : GGGTGTGTCCCCAATGTCTCTGGAGAGAGGGCGAGGTCGTGACTGTCCGGAAGCTTACAGGAGGCACCAATTGTTTTGTAGATCTAGGGTTTGTTT : 3331

                                                3400                                                    *
BrTT8A-b_g : ------------------------------------------------------------------------------------------------ :    -
BrTT8A-y_g : ------------------------------------------------------------------------------------------------ :    -
BnTT8A-b_g : ------------------------------------------------------------------------------------------------ :    -
BnTT8A-y_g : ------------------------------------------------------------------------------------------------ :    -
BjTT8A-d_g : ------------------------------------------------------------------------------------------------ :    -
BjTT8A-y_g : TGTCGCGGTTCTGGGTCAGATCTGTGTCGGAGGTTTGCCGGACCGAGAGCGTCGCTGCCTCTCTTCCACCGGG-TTATCGCCTCCTCTGCTCTCCC : 3426
BjTT8A-y_r : TGTCGCGGTTCTGGGTCAGATCTGTGTCGGAGGTTTGCCGGACCGAGAGCGTCGCTGCCTCTCTTCCACCGGGTTTATCGCCTCCTCTGCTCTCCC : 3427
```

```
                                                 3500                                                         *
BrTT8A-b_g : ------------------------------------------------------------------------------------------------ :    -
BrTT8A-y_g : ------------------------------------------------------------------------------------------------ :    -
BnTT8A-b_g : ------------------------------------------------------------------------------------------------ :    -
BnTT8A-y_g : ------------------------------------------------------------------------------------------------ :    -
BjTT8A-d_g : ------------------------------------------------------------------------------------------------ :    -
BjTT8A-y_g : CTGTCTCTCTCTCTTCGGTTGCTGTTGCAGGTGCTTCCCCATCTTCGTACTCTGGTCCGAGATGTAAAGGCGAGTCTGAGGAACTCGTTGGGGACC : 3522
BjTT8A-y_r : CTGTCTCTCTCTCTTCGGTTGCTGTTGCAGGTGCTTCCCCATCTTCGTACTCTGGTCCGAGATGTAAAGGCGAGTCTGAGGAACTCGTTGGGGACC : 3523

                                                     3600
BrTT8A-b_g : ------------------------------------------------------------------------------------------------ :    -
BrTT8A-y_g : ------------------------------------------------------------------------------------------------ :    -
BnTT8A-b_g : ------------------------------------------------------------------------------------------------ :    -
BnTT8A-y_g : ------------------------------------------------------------------------------------------------ :    -
BjTT8A-d_g : ------------------------------------------------------------------------------------------------ :    -
BjTT8A-y_g : TCCTCCCGTTCTGATGTCTGAGCATTTACCATGGTGTTTGGTTGGTGATCCTTTCGATGGGTTCAAGATGGTCTAGGGTTCGTGCCGTTCTCTCTT : 3618
BjTT8A-y_r : TCCTCCCGTTCTGATGTCTGAGCATTTACCATGGTGTTTGGTTGGTGATCCTTTCGATGGGTTCAAGATGGTCTAGGGTTCGTGCCGTTCTCTCTT : 3619

              *                                          3700
BrTT8A-b_g : ------------------------------------------------------------------------------------------------ :    -
BrTT8A-y_g : ------------------------------------------------------------------------------------------------ :    -
BnTT8A-b_g : ------------------------------------------------------------------------------------------------ :    -
BnTT8A-y_g : ------------------------------------------------------------------------------------------------ :    -
BjTT8A-d_g : ------------------------------------------------------------------------------------------------ :    -
BjTT8A-y_g : TCGTCAGATCTCCATAGTCGGGTCTAGCCTCTCTTCCACCTGCTGATTGGAGC--TTTCGTCTCCTCCGCCCTTCAAGTTTCAGAAGGAAGCCGTC : 3712
BjTT8A-y_r : TCGTCAGATCTCCATAGTCGGGTCTAGCCTCTCTTCCACCTGCTGATTGGAGCTTTTTCGTCTCCTCCGCCCTTCAAGTTTCAGAAGGAAGCCGTC : 3715

                  *                                          3800
BrTT8A-b_g : ------------------------------------------------------------------------------------------------ :    -
BrTT8A-y_g : ------------------------------------------------------------------------------------------------ :    -
BnTT8A-b_g : ------------------------------------------------------------------------------------------------ :    -
BnTT8A-y_g : ------------------------------------------------------------------------------------------------ :    -
BjTT8A-d_g : ------------------------------------------------------------------------------------------------ :    -
BjTT8A-y_g : AGCCGTTACCTGCCTTTCCGATGCTCTGCAGCCAATCCTTCTGTGCGGTACTTGCGTGATGGGACCGGAGCTGAGCTGTATAACCTCCGCTTCCAT : 3808
BjTT8A-y_r : AGCCGTTACCTGCCTTTCCGATGCTCTGCAGCCAATCCTTCTGTGCGGTACTTGCGTGATGGGACCGGAGCTGAGCTGTATAACCTCCGCTTCCAT : 3811

                      *                                          3900
BrTT8A-b_g : ------------------------------------------------------------------------------------------------ :    -
BrTT8A-y_g : ------------------------------------------------------------------------------------------------ :    -
BnTT8A-b_g : ------------------------------------------------------------------------------------------------ :    -
BnTT8A-y_g : ------------------------------------------------------------------------------------------------ :    -
BjTT8A-d_g : ------------------------------------------------------------------------------------------------ :    -
BjTT8A-y_g : CAGCCTGTCCCTCACCTGCTCTTCATCTCCTGGCTTATTTGTGTTGGGTGGGACTCACAGGGACACGCCTTGCCGTCTTCTGGAGTGGACAGCGTC : 3904
BjTT8A-y_r : CAGCCTGTCCCTCACCTGCTCTTCATCTCCTGGCTTATTTGTGTTGGGTGGGACTCACAGGGACACGCCTTGCCGTCTTCTGGAGTGGACAGCGTC : 3907

                          *                                          4000
BrTT8A-b_g : ------------------------------------------------------------------------------------------------ :    -
BrTT8A-y_g : ------------------------------------------------------------------------------------------------ :    -
BnTT8A-b_g : ------------------------------------------------------------------------------------------------ :    -
BnTT8A-y_g : ------------------------------------------------------------------------------------------------ :    -
BjTT8A-d_g : ------------------------------------------------------------------------------------------------ :    -
BjTT8A-y_g : TGGAGTAGATTTCACTGGGTCTCGAAGGCCGGA-CCAAGGCTTGGTCTCCCTTGCTTCGCCTGTCGCCCCTGTTGGAGTCGCCTTCGGGATGGTCT : 3999
BjTT8A-y_r : TGGAGTAGATTTCACTGGGTCTCGAAGGCCGGACCCAAGGCTTGGTCTCCCTTGCTTCGCCTGTCGCCCCTGTTGGAGTCGCCTTCGGGATGGTCT : 4003

                              *                                          4100
BrTT8A-b_g : ------------------------------------------------------------------------------------------------ :    -
BrTT8A-y_g : ------------------------------------------------------------------------------------------------ :    -
BnTT8A-b_g : ------------------------------------------------------------------------------------------------ :    -
BnTT8A-y_g : ------------------------------------------------------------------------------------------------ :    -
BjTT8A-d_g : ------------------------------------------------------------------------------------------------ :    -
BjTT8A-y_g : GCGGGTCCTTGGACCATTCTCCGGTTGAATCCTCCATTGATCTCTGTTGTTTGATGCAGGTCCCGGCATCATGTCTGTTCCGAGATCTACTGAAGC : 4095
BjTT8A-y_r : GCGGGTCCTTGGACCATTCTCCGGTTGAATCCTCCATTGATCTCTGTTGTTTGATGCAGGTCCCGGCATCATGTCTGTTCCGAGATCTACTGAAGC : 4099

                                  *                                          4200
BrTT8A-b_g : ------------------------------------------------------------------------------------------------ :    -
BrTT8A-y_g : ------------------------------------------------------------------------------------------------ :    -
BnTT8A-b_g : ------------------------------------------------------------------------------------------------ :    -
BnTT8A-y_g : ------------------------------------------------------------------------------------------------ :    -
BjTT8A-d_g : ------------------------------------------------------------------------------------------------ :    -
BjTT8A-y_g : TAAATGTTTGCTCCTGGTATCTTCAATTGTCAAGCTTTGGTCACATTCTTTGATTGTGATCAAACCTTTCATTGTCCTAGAAGAGTCTTTTTTACC : 4191
BjTT8A-y_r : TAAATGTTTGCTCCTGGTATCTTCAATTGTCAAGCTTTGGTCACATTCTTTGATTGTGATCAAACCTTTCATTGTCCTAGAAGAGTCTTTTTTACC : 4195

                                      *                                          4300
BrTT8A-b_g : ------------------------------------------------------------------------------------------------ :    -
BrTT8A-y_g : ------------------------------------------------------------------------------------------------ :    -
BnTT8A-b_g : ------------------------------------------------------------------------------------------------ :    -
BnTT8A-y_g : ------------------------------------------------------------------------------------------------ :    -
BjTT8A-d_g : ------------------------------------------------------------------------------------------------ :    -
BjTT8A-y_g : TTTTTTTGTGGCTCTTTTAGGCAGCTTGCTTTGATAAGCTTCTTAGATGCTAGGATTTGATCTTTCAAAACCTTTGTATCCCCTGTGTCATTAGTT : 4287
BjTT8A-y_r : TTTTTTTGTGGCTCTTTTAGGCAGCTTGCTTTGATAAGCTTCTTAGATGCTAGGATTTGATCTTTCAAAACCTTTGTATCCCCTGTGTCATTAGTT : 4291
```

```
                                     *    ↓insertion end                    4400
BrTT8A-b_g : ---------------------------------ACAACACTAAAAATAAGAGGCTACCGCGAGAAGAGCTTAACCATGTGGTGGCCGAGCGACGCA : 3105
BrTT8A-y_g : ---------------------------------ACAACACTAAAAATAAGAGGCTACCGCGAGAAGAGCTTAACCATGTGGTGGCCGAGCGACGCA : 3115
BnTT8A-b_g : ---------------------------------ACAACACTAAAAATAAGAGGCTACCGCGAGAAGAGCTTAACCATGTGGTGGCCGAGCGACGCA : 3107
BnTT8A-y_g : ---------------------------------ACAACACTAAAAATAAGAGGCTACCGCGAGAAGAGCTTAACCATGTGGTGGCCGAGCGACGCA : 3107
BjTT8A-d_g : ---------------------------------ACAACACTAAAAATAAGAGGCTACCGCGAGAAGAGCTTAACCATGTGGTGGCCGAGCGACGCA : 3107
BjTT8A-y_g : TCTAATGAAAATTCACATACTTAACAAAAAAAAAAAACACTAAAAATAAGAGGCTACCGCGAGAAGAGCTTAACCATGTGGTGGCCGAGCGACGCA : 4383
BjTT8A-y_r : TCTAATGAAAATTCACATACTTAAC-AAAAAAAAAAACACTAAAAATAAGAGGCTACCGCGAGAAGAGCTTAACCATGTGGTGGCCGAGCGACGCA : 4386
                                              A AACACTAAAAATAAGAGGCTACCGCGAGAAGAGCTTAACCATGTGGTGGCCGAGCGACGCA

                                          *                                        4500
BrTT8A-b_g : GAAGAGAGAAGCTAAATGAGAGATTCATAACGTTGAGATCATTGGTTCCATTTGTGACCAAGATGGATAAAGTCTCGATCCTTGGAGACACCATTG : 3201
BrTT8A-y_g : GAAGAGAGAAGCTAAATGAGAGATTCATAACGTTGAGATCATTGGTTCCATTTGTGACCAAGATGGATAAAGTCTCGATCCTTGGAGACACCATTG : 3211
BnTT8A-b_g : GAAGAGAGAAGCTAAATGAGAGATTCATAACGTTGAGATCATTGGTTCCATTTGTGACCAAGATGGATAAAGTCTCGATCCTTGGAGACACCATTG : 3203
BnTT8A-y_g : GAAGAGAGAAGCTAAATGAGAGATTCATAACGTTGAGATCATTGGTTCCATTTGTGACCAAGATGGATAAAGTCTCGATCCTTGGAGACACCATTG : 3203
BjTT8A-d_g : GAAGAGAGAAGCTAAATGAGAGATTCATAACGTTGAGATCATTGGTTCCATTTGTGACCAAGATGGATAAAGTCTCGATCCTTGGAGACACCATTG : 3203
BjTT8A-y_g : GAAGAGAGAAGCTAAATGAGAGATTCATAACGTTGAGATCATTGGTTCCATTTGTGACCAAGATGGATAAAGTCTCGATCCTTGGAGACACCATTG : 4479
BjTT8A-y_r : GAAGAGAGAAGCTAAATGAGAGATTCATAACGTTGAGATCATTGGTTCCATTTGTGACCAAGATGGATAAAGTCTCGATCCTTGGAGACACCATTG : 4482
             GAAGAGAGAAGCTAAATGAGAGATTCATAACGTTGAGATCATTGGTTCCATTTGTGACCAAGATGGATAAAGTCTCGATCCTTGGAGACACCATTG

                                              *                                        4600
BrTT8A-b_g : AATACGTAAACCATCTTTCTAAGAGGATACATGAGCTGGAATCTACTCATCACGAGCCAAACCAAAAGCGGATGCGTATCGGTAAGGGAAGAACTT : 3297
BrTT8A-y_g : AATACGTAAACCATCTTTCTAAGAGGATACATGAGCTGGAATCTACTCATCACGAGCCAAACCAAAAGCGGATGCGTATCGGTAAGGGAAGAACTT : 3307
BnTT8A-b_g : AATACGTAAACCATCTTTCTAAGAGGATACATGAGCTGGAATCTACTCATCACGAGCCAAACCAAAAGCGGATGCGTATCGGTAAGGGAAGAACTT : 3299
BnTT8A-y_g : AATACGTAAACCATCTTTCTAAGAGGATACATGAGCTGGAATCTACTCATCACGAGCCAAACCAAAAGCGGATGCGTATCGGTAAGGGAAGAACTT : 3299
BjTT8A-d_g : AATACGTAAACCATCTTTCTAAGAGGATACATGAGCTGGAATCTACTCATCACGAGCCAAACCAAAAGCGGATGCGTATCGGTAAGGGAAGAACTT : 3299
BjTT8A-y_g : AATACGTAAACCATCTTTCTAAGAGGATACATGAGCTGGAATCTACTCATCACGAGCCAAACCAAAAGCGGATGCGTATCGGTAAGGGAAGAACTT : 4575
BjTT8A-y_r : AATACGTAAACCATCTTTCTAAGAGGATACATGAGCTGGAATCTACTCATCACGAGCCAAACCAAAAGCGGATGCGTATCGGTAAGGGAAGAACTT : 4578
             AATACGTAAACCATCTTTCTAAGAGGATACATGAGCTGGAATCTACTCATCACGAGCCAAACCAAAAGCGGATGCGTATCGGTAAGGGAAGAACTT

                                                  *                                        4700
BrTT8A-b_g : GGGAAGAGGTGGAGGTTTCCATTATAGAGAGCGATGTTTTGTTAGAGATGAGATGCGAGTACCGAGATGGTTTATTGCTCAACATTCTTCAGGTAC : 3393
BrTT8A-y_g : GGGAAGAGGTGGAGGTTTCCATTATAGAGAGCGATGTTTTGTTAGAGATGAGATGCGAGTACCGAGATGGTTTATTGCTCAACATTCTTCAGGTAC : 3403
BnTT8A-b_g : GGGAAGAGGTGGAGGTTTCCATTATAGAGAGCGATGTTTTGTTAGAGATGAGATGCGAGTACCGAGATGGTTTATTGCTCAACATTCTTCAGGTAC : 3395
BnTT8A-y_g : GGGAAGAGGTGGAGGTTTCCATTATAGAGAGCGATGTTTTGTTAGAGATGAGATGCGAGTACCGAGATGGTTTATTGCTCAACATTCTTCAGGTAC : 3395
BjTT8A-d_g : GGGAAGAGGTGGAGGTTTCCATTATAGAGAGCGATGTTTTGTTAGAGATGAGATGCGAGTACCGAGATGGTTTATTGCTCAACATTCTTCAGGTAC : 3395
BjTT8A-y_g : GGGAAGAGGTGGAGGTTTCCATTATAGAGAGCGATGTTTTGTTAGAGATGAGATGCGAGTACCGAGATGGTTTATTGCTCAACATTCTTCAGGTAC : 4671
BjTT8A-y_r : GGGAAGAGGTGGAGGTTTCCATTATAGAGAGCGATGTTTTGTTAGAGATGAGATGCGAGTACCGAGATGGTTTATTGCTCAACATTCTTCAGGTAC : 4674
             GGGAAGAGGTGGAGGTTTCCATTATAGAGAGCGATGTTTTGTTAGAGATGAGATGCGAGTACCGAGATGGTTTATTGCTCAACATTCTTCAGGTAC

                                                      *                                        4800
BrTT8A-b_g : TTAAGGAGCTGGGTATAGAGACCACTGCGGTTCACACTGCCTTGAACGACAATCATTTTGAGGCAGAGATAAGGGCGAAAGTGAGAGGGAAGAAAC : 3489
BrTT8A-y_g : TTAAGGAGCTGGGTATAGAGACCACTGCGGTTCACACTGCCTTGAACGACAATCATTTTGAGGCAGAGATAAGGGCGAAAGTGAGAGGGAAGAAAC : 3499
BnTT8A-b_g : TTAAGGAGCTGGGTATAGAGACCACTGCCGTTCACACTGCCTTGAACGACAATCATTTTGAGGCAGAGATAAGGGCGAAAGTGAGAGGGAAGAAAC : 3491
BnTT8A-y_g : TTAAGGAGCTGGGTATAGAGACCACTGCCGTTCACACTGCCTTGAACGACAATCATTTTGAGGCAGAGATAAGGGCGAAAGTGAGAGGGAAGAAAC : 3491
BjTT8A-d_g : TTAAGGAGCTGGGTATAGAGACCACTGCGGTTCACACTGCCTTGAACGACAATCATTTTGAGGCAGAGATAAGGGCGAAAGTGAGAGGGAAGAAAC : 3491
BjTT8A-y_g : TTAAGGAGCTGGGTATAGAGACCACTGCGGTTCACACTGCCTTGAACGACAATCATTTTGAGGCAGAGATAAGGGCGAAAGTGAGAGGGAAGAAAC : 4767
BjTT8A-y_r : TTAAGGAGCTGGGTATAGAGACCACTGCGGTTCACACTGCCTTGAACGACAATCATTTTGAGGCAGAGATAAGGGCGAAAGTGAGAGGGAAGAAAC : 4770
             TTAAGGAGCTGGGTATAGAGACCACTGC GTTCACACTGCCTTGAACGACAATCATTTTGAGGCAGAGATAAGGGCGAAAGTGAGAGGGAAGAAAC

                                                          *
BrTT8A-b_g : CAACCATTGCTGAGGTTAAAATAGCCATCCATCAAATCATATATAATAATAAACTCTAG : 3548
BrTT8A-y_g : CAACCATTGCTGAGGTTAAAATAGCCATCCATCAAATCATATATAATAATAAACTCTAG : 3558
BnTT8A-b_g : CAACCATTGCTGAGGTTAAAATAGCCATCCATCAAATCATATATAATAATAAACTCTAG : 3550
BnTT8A-y_g : CAACCATTGCTGAGGTTAAAATAGCCATCCATCAAATCATATATAATAATAAACTCTAG : 3550
BjTT8A-d_g : CAACCATTGCTGAGGTTAAAATAGCCATCCATCAAATCATATATAATAATAAACTCTAG : 3550
BjTT8A-y_g : CAACCATTGCTGAGGTTAAAATAGCCATCCATCAAATCATATATAATAATAAACTCTAG : 4826
BjTT8A-y_r : CAACCATTGCTGAGGTTAAAATAGCCATCCATCAAATCATATATAATAATAAACTCTAG : 4829
             CAACCATTGCTGAGGTTAAAATAGCCATCCATCAAATCATATATAATAATAAACTCTAG
```
