# Supplementary material for: Metabolite Profiling and Transcriptome Analysis Provide Insight into Seed Coat Color in Brassica juncea
Source: Int J Mol Sci. 2021 Jul 5;22(13):7215. doi: 10.3390/ijms22137215 (PMC8268557; doi:10.3390/ijms22137215)
Supplement: Supplementary file 1 [file ijms-22-07215-s001.zip › ijms-1245090-SI/Supplementary Figure S6.pdf]

```
extron1                                                                 *
BniTT8B-b : ATGGATGAATCAAGTATTATACAGGTATGGAAAGTGATCGGAGCTGAGGAAAAAGAGATTCAAGGGCTACTTAAGGCGGTGGTACAATCTGTGGGGT :   97
BcaTT8B-b : ATGGATGAATCAAGTATTATACAGGTATGGAAAGTGATCGGAGCTGAGGAAAAAGAGATTCAAGGGCTACTTAAGGCGGTGGTACAATCTGTGGGGT :   97
BcaTT8B-y : ATGGATGAATCAAGTATTATACAGGTATGGAAAGTGATCGGAGCTGAGGAAAAAGAGATTCAAGGGCTACTTAAGGCGGTGGTACAATCTGTGGGGT :   97
BjuTT8B-d : ATGGATGAATCAAGTATTATACAGGTATGGAAAGTGATCGGAGCTGAGGAAAAAGAGATTCAAGGGCTACTTAAGGCGGTGGTACAATCTGTGGGGT :   97
BjuTT8B-y : ATGGATGAATCAAGTATTATACAGGTATGGAAAGTGATCGGAGCTGAGGAAAAAGAGATTCAAGGGCTACTTAAGGCGGTGGTACAATCTGTGGGGT :   97
            ATGGATGAATCAAGTATTATACAGGTATGGAAAGTGATCGGAGCTGAGGAAAAAGAGATTCAAGGGCTACTTAAGGCGGTGGTACAATCTGTGGGGT

            100                                                                     *
BniTT8B-b : GGACTTATAGTCTCTTCTGGCAACTTTGTCCTCAACGAAGGTTCTCTTTTCATTTCATCCATCTCTCACAATATATTTATAATCCTTATCAATTATA :  194
BcaTT8B-b : GGACTTATAGTCTCTTCTGGCAACTTTGTCCTCAACGAAGGTTCTCTTTTCATTTCATCCATCTCTCACAATATATTTATAATCCTTATCAATTATA :  194
BcaTT8B-y : GGACTTATAGTCTCTTCTGGCAACTTTGTCCTCAACGAAGGTTCTCTTTTCATTTCATCCATCTCTCACAATATATTTATAATCCTTATCAATTATA :  194
BjuTT8B-d : GGACTTATAGTCTCTTCTGGCAACTTTGTCCTCAACGAAGGTTCTCTTTTCATTTCATCCATCTCTCACAATATATTTATAATCCTTATCAATTATA :  194
BjuTT8B-y : GGACTTATAGTCTCTTCTGGCAACTTTGTCCTCAACGAAGGTTCTCTTTTCATTTCATCCATCTCTCACAATATATTTATAATCCTTATCAATTATA :  194
            GGACTTATAGTCTCTTCTGGCAACTTTGTCCTCAACGAAGGTTCTCTTTTCATTTCATCCATCTCTCACAATATATTTATAATCCTTATCAATTATA

                200                                  extron2                    *
BniTT8B-b : ATAAGTAGAAGTGACCAAATGTTAAATCGATTAGGAAATTGGTGTGGAGTAGTGGATTCTACAACGGTGCAATAAAGACTAGAAAGACAACTCAGCC :  291
BcaTT8B-b : ATAAGTAGAAGTGACCAAATGTTAAATCGATTAGGAAATTGGTGTGGAGTAGTGGATTCTACAACGGTGCAATAAAGACTAGAAAGACAACTCAGCC :  291
BcaTT8B-y : ATAAGTAGAAGTGACCAAATGTTAAATCGATTAGGAAATTGGTGTGGAGTAGTGGATTCTACAACGGTGCAATAAAGACTAGAAAGACAACTCAGCC :  291
BjuTT8B-d : ATAAGTAGAAGTGACCAAATGTTAAATCGATTAGGAAATTGGTGTGGAGTAGTGGATTCTACAACGGTGCAATAAAGACTAGAAAGACAACTCAGCC :  291
BjuTT8B-y : ATAAGTAGAAGTGACCAAATGTTAAATCGATTAGGAAATTGGTGTGGAGTAGTGGATTCTACAACGGTGCAATAAAGACTAGAAAGACAACTCAGCC :  291
            ATAAGTAGAAGTGACCAAATGTTAAATCGATTAGGAAATTGGTGTGGAGTAGTGGATTCTACAACGGTGCAATAAAGACTAGAAAGACAACTCAGCC

                    300                                                                 *
BniTT8B-b : GGCGGAAGTTACGGCTGAAGAGGCTGCGTTGGAGAGAACCCAACAGCTCATGGAGCTTTACCAGACGCTTTTTTCCGGAGAATCATCGATGGAAGCG :  388
BcaTT8B-b : GGCGGAAGTTACGGCTGAAGAGGCTGCGTTGGAGAGAACCCAACAGCTCATGGAGCTTTACCAGACGCTTTTTTCCGGAGAATCATCGATGGAAGCG :  388
BcaTT8B-y : GGCGGAAGTTACGGCTGAAGAGGCTGCGTTGGAGAGAACCCAACAGCTCATGGAGCTTTACCAGACGCTTTTTTCCGGAGAATCATCGATGGAAGCG :  388
BjuTT8B-d : GGCGGAAGTTACGGCTGAAGAGGCTGCGTTGGAGAGAACCCAACAGCTCATGGAGCTTTACCAGACGCTTTTTTCCGGAGAATCATCGATGGAAGCG :  388
BjuTT8B-y : GGCGGAAGTTACGGCTGAAGAGGCTGCGTTGGAGAGAACCCAACAGCTCATGGAGCTTTACCAGACGCTTTTTTCCGGAGAATCATCGATGGAAGCG :  388
            GGCGGAAGTTACGGCTGAAGAGGCTGCGTTGGAGAGAACCCAACAGCTCATGGAGCTTTACCAGACGCTTTTTTCCGGAGAATCATCGATGGAAGCG

                        400                                                                 *
BniTT8B-b : AGGGCTTGCACAGCACTGTCGCCGGAGGATTTGACGGACACTGAGTGGTTTTATGTGCTGTGTTTCACTTACTCTTTTGAACCTCCTTCTGGGTACA :  485
BcaTT8B-b : AGGGCTTGCACAGCACTGTCGCCGGAGGATTTGACGGACACTGAGTGGTTTTATGTGCTGTGTTTCACTTACTCTTTTGAACCTCCTTCTGGGTACA :  485
BcaTT8B-y : AGGGCTTGCACAGCACTGTCGCCGGAGGATTTGACGGACACTGAGTGGTTTTATGTGCTGTGTTTCACTTACTCTTTTGAACCTCCTTCTGGGTACA :  485
BjuTT8B-d : AGGGCTTGCACAGCACTGTCGCCGGAGGATTTGACGGACACTGAGTGGTTTTATGTGCTGTGTTTCACTTACTCTTTTGAACCTCCTTCTGGGTACA :  485
BjuTT8B-y : AGGGCTTGCACAGCACTGTCGCCGGAGGATTTGACGGACACTGAGTGGTTTTATGTGCTGTGTTTCACTTACTCTTTTGAACCTCCTTCTGGGTACA :  485
            AGGGCTTGCACAGCACTGTCGCCGGAGGATTTGACGGACACTGAGTGGTTTTATGTGCTGTGTTTCACTTACTCTTTTGAACCTCCTTCTGGGTACA

                            500                                                                 *
BniTT8B-b : ACAACTCTCTCTCTCTATCTCTTCAAAGTTTTTTTTTTCTTTTCAAAAAGACTACTCAGAGTTTCTTAATTTGTCTTTTTCATCTTCTCTTAGTGAA :  582
BcaTT8B-b : ACAACTCTCTCTCTCTATCTCTTCAAAGTTTTTTTTTTCTTTTCAAAAAGACTACTCAGAGTTTCTTAATTTGTCTTTTTCATCTTCTCTTAGTGAA :  582
BcaTT8B-y : ACAACTCTCTCTCTCTATCTCTTCAAAGTTTTTTTTTTCTTTTCAAAAAGACTACTCAGAGTTTCTTAATTTGTCTTTTTCATCTTCTCTTAGTGAA :  582
BjuTT8B-d : ACAACTCTCTCTCTCTATCTCTTCAAAGTTTTTTTTTTCTTTTCAAAAAGACTACTCAGAGTTTCTTAATTTGTCTTTTTCATCTTCTCTTAGTGAA :  582
BjuTT8B-y : ACAACTCTCTCTCTCTATCTCTTCAAAGTTTTTTTTTTCTTTTCAAAAAGACTACTCAGAGTTTCTTAATTTGTCTTTTTCATCTTCTCTTAGTGAA :  582
            ACAACTCTCTCTCTCTATCTCTTCAAAGTTTTTTTTTTCTTTTCAAAAAGACTACTCAGAGTTTCTTAATTTGTCTTTTTCATCTTCTCTTAGTGAA

                                600                                                                 *
BniTT8B-b : GACAAAAATAGCATTGTGTGTTAAATGCGAATCACAAATACTATGGAAGCATTAAAGACAAACTGGGGAGTTTAAGTTACTGAAAGAAGAAATGTAT :  679
BcaTT8B-b : GACAAAAATAGCATTGTGTGTTAAATGCGAATCACAAATACTATGGAAGCATTAAAGACAAACTGGGGAGTTTAAGTTACTGAAAGAAGAAATGTAT :  679
BcaTT8B-y : GACAAAAATAGCATTGTGTGTTAAATGCGAATCACAAATACTATGGAAGCATTAAAGACAAACTGGGGAGTTTAAGTTACTGAAAGAAGAAATGTAT :  679
BjuTT8B-d : GACAAAAATAGCATTGTGTGTTAAATGCGAATCACAAATACTATGGAAGCATTAAAGACAAACTGGGGAGTTTAAGTTACTGAAAGAAGAAATGTAT :  679
BjuTT8B-y : GACAAAAATAGCATTGTGTGTTAAATGCGAATCACAAATACTATGGAAGCATTAAAGACAAACTGGGGAGTTTAAGTTACTGAAAGAAGAAATGTAT :  679
            GACAAAAATAGCATTGTGTGTTAAATGCGAATCACAAATACTATGGAAGCATTAAAGACAAACTGGGGAGTTTAAGTTACTGAAAGAAGAAATGTAT

                                    700                                                                 *
BniTT8B-b : TAAAGTTTGTGAAAACGTACACTTCATTTTGGTGAACATATCTGGACCGTTGAGATGATCTTATTGGTTTGTTTATTGATTATCTAAAGTAGAAGCA :  776
BcaTT8B-b : TAAAGTTTGTGAAAACGTACACTTCATTTTGGTGAACATATCTGGACCGTTGAGATGATCTTATTGGTTTGTTTATTGATTATCTAAAGTAGAAGCA :  776
BcaTT8B-y : TAAAGTTTGTGAAAACGTACACTTCATTTTGGTGAACATATCTGGACCGTTGAGATGATCTTATTGGTTTGTTTATTGATTATCTAAAGTAGAAGCA :  776
BjuTT8B-d : TAAAGTTTGTGAAAACGTACACTTCATTTTGGTGAACATATCTGGACCGTTGAGATGATCTTATTGGTTTGTTTATTGATTATCTAAAGTAGAAGCA :  776
BjuTT8B-y : TAAAGTTTGTGAAAACGTACACTTCATTTTGGTGAACATATCTGGACCGTTGAGATGATCTTATTGGTTTGTTTATTGATTATCTAAAGTAGAAGCA :  776
            TAAAGTTTGTGAAAACGTACACTTCATTTTGGTGAACATATCTGGACCGTTGAGATGATCTTATTGGTTTGTTTATTGATTATCTAAAGTAGAAGCA

                                        800                                                                 *
BniTT8B-b : TAGATGATAAATGCATAACAAAGTGTGTTAGTTATCGGTATAATTAATGTTTTTTCTCTATGGAGGAAAAAAATCAAAATATAAATGTGGAAGTATT :  873
BcaTT8B-b : TAGATGATAAATGCATAACAAAGTGTGTTAGTTATCGGTATAATTAATGTTTTTTCTCTATGGAGGAAAAAAATCAAAATATAAATGTGGAAGTATT :  873
BcaTT8B-y : TAGATGATAAATGCATAACAAAGTGTGTTAGTTATCGGTATAATTAATGTTTTTTCTCTATGGAGGAAAAAAATCAAAATATAAATGTGGAAGTATT :  873
BjuTT8B-d : TAGATGATAAATGCATAACAAAGTGTGTTAGTTATCGGTATAATTAATGTTTTTTCTCTATGGAGGAAAAAAATCAAAATATAAATGTGGAAGTATT :  873
BjuTT8B-y : TAGATGATAAATGCATAACAAAGTGTGTTAGTTATCGGTATAATTAATGTTTTTTCTCTATGGAGGAAAAAAATCAAAATATAAATGTGGAAGTATT :  873
            TAGATGATAAATGCATAACAAAGTGTGTTAGTTATCGGTATAATTAATGTTTTTTCTCTATGGAGGAAAAAAATCAAAATATAAATGTGGAAGTATT

                      extron3                   900                                                                 *
BniTT8B-b : AATTTGTAGGATGCCAGGAAAGGCGTATGCCAGGAGGAAGCACGTATGGATAAGTGGTGCAAATGAGGTTGACAGTAAAATCTTCTCTAGGGCTATT :  970
BcaTT8B-b : AATTTGTAGGATGCCAGGAAAGGCGTATGCCAGGAGGAAGCACGTATGGATAAGTGGTGCAAATGAGGTTGACAGTAAAATCTTCTCTAGGGCTATT :  970
BcaTT8B-y : AATTTGTAGGATGCCAGGAAAGGCGTATGCCAGGAGGAAGCACGTATGGATAAGTGGTGCAAATGAGGTTGACAGTAAAATCTTCTCTAGGGCTATT :  970
BjuTT8B-d : AATTTGTAGGATGCCAGGAAAGGCGTATGCCAGGAGGAAGCACGTATGGATAAGTGGTGCAAATGAGGTTGACAGTAAAATCTTCTCTAGGGCTATT :  970
BjuTT8B-y : AATTTGTAGGATGCCAGGAAAGGCGTATGCCAGGAGGAAGCACGTATGGATAAGTGGTGCAAATGAGGTTGACAGTAAAATCTTCTCTAGGGCTATT :  970
            AATTTGTAGGATGCCAGGAAAGGCGTATGCCAGGAGGAAGCACGTATGGATAAGTGGTGCAAATGAGGTTGACAGTAAAATCTTCTCTAGGGCTATT

                                                1000                                                                *
BniTT8B-b : TCTGCAAAGGTTTATTTCCTTTTATTCATTCACCACTACACTGTGTATTTACTTGTACTTATTTAGATATACGCAATTTTATATATCTCATTCTTCA : 1067
BcaTT8B-b : TCTGCAAAGGTTTATTTCCTTTTATTCATTCACCACTACACTGTGTATTTACTTGTACTTATTTAGATATACGCAATTTTATATATCTCATTCTTCA : 1067
BcaTT8B-y : TCTGCAAAGGTTTATTTCCTTTTATTCATTCACCACTACACTGTGTATTTACTTGTACTTATTTAGATATACGCAATTTTATATATCTCATTCTTCA : 1067
BjuTT8B-d : TCTGCAAAGGTTTATTTCCTTTTATTCATTCACCACTACACTGTGTATTTACTTGTACTTATTTAGATATACGCAATTTTATATATCTCATTCTTCA : 1067
BjuTT8B-y : TCTGCAAAGGTTTATTTCCTTTTATTCATTCACCACTACACTGTGTATTTACTTGTACTTATTTAGATATACGCAATTTTATATATCTCATTCTTCA : 1067
            TCTGCAAAGGTTTATTTCCTTTTATTCATTCACCACTACACTGTGTATTTACTTGTACTTATTTAGATATACGCAATTTTATATATCTCATTCTTCA
```

```
                                    1100           extron4                    *
BniTT8B-b : AACTAATTAATTTATCTTCTTCTTTACTTGAATGCTCTTTCTACATTAGAGTGCCAAAATTCAGGTAAATGTTGCCGTTATTAATTGATTATTTCAT : 1164
BcaTT8B-b : AACTAATTAATTTATCTTCTTCTTTACTTGAATGCTCTTTCTACATTAGAGTGCCAAAATTCAGGTAAATGTTGCCGTTATTAATTGATTATTTCAT : 1164
BcaTT8B-y : AACTAATTAATTTATCTTCTTCTTTACTTGAATGCTCTTTCTACATTAGAGTGCCAAAATTCAGGTAAATGTTGCCGTTATTAATTGATTATTTCAT : 1164
BjuTT8B-d : AACTAATTAATTTATCTTCTTCTTTACTTGAATGCTCTTTCTACATTAGAGTGCCAAAATTCAGGTAAATGTTGCCGTTATTAATTGATTATTTCAT : 1164
BjuTT8B-y : AACTAATTAATTTATCTTCTTCTTTACTTGAATGCTCTTTCTACATTAGAGTGCCAAAATTCAGGTAAATGTTGCCGTTATTAATTGATTATTTCAT : 1164
            AACTAATTAATTTATCTTCTTCTTTACTTGAATGCTCTTTCTACATTAGAGTGCCAAAATTCAGGTAAATGTTGCCGTTATTAATTGATTATTTCAT

                                        1200                                                *   extron5
BniTT8B-b : TTTCGTAGAAATGAAAGGTATCAATTAATAAAGTTATTTTACATATTTGAGAATTGTTTTGTGATGAAAAACAAATAAAAACAGACAATGGTTTGCA : 1261
BcaTT8B-b : TTTCGTAGAAATGAAAGGTATCAATTAATAAAGTTATTTTACATATTTGAGAATTGTTTTGTGATGAAAAACAAATAAAAACAGACAATGGTTTGCA : 1261
BcaTT8B-y : TTTCGTAGAAATGAAAGGTATCAATTAATAAAGTTATTTTACATATTTGAGAATTGTTTTGTGATGAAAAACAAATAAAAACAGACAATGGTTTGCA : 1261
BjuTT8B-d : TTTCGTAGAAATGAAAGGTATCAATTAATAAAGTTATTTTACATATTTGAGAATTGTTTTGTGATGAAAAACAAATAAAAACAGACAATGGTTTGCA : 1261
BjuTT8B-y : TTTCGTAGAAATGAAAGGTATCAATTAATAAAGTTATTTTACATATTTGAGAATTGTTTTGTGATGAAAAACAAATAAAAACAGACAATGGTTTGCA : 1261
            TTTCGTAGAAATGAAAGGTATCAATTAATAAAGTTATTTTACATATTTGAGAATTGTTTTGTGATGAAAAACAAATAAAAACAGACAATGGTTTGCA

                                            1300                                                  *
BniTT8B-b : TTCCCGTGCTTGATGGCGTTTTGGAACTCGGCACAACGAACAAGGTAAATTCTCTATTTATGTCGGTACAAAAATGTAGACGAGTACATCCAGCTCG : 1358
BcaTT8B-b : TTCCCGTGCTTGATGGCGTTTTGGAACTCGGCACAACGAACAAGGTAAATTCTCTATTTATGTCGGTACAAAAATGTAGACGAGTACATCCAGCTCG : 1358
BcaTT8B-y : TTCCCGTGCTTGATGGCGTTTTGGAACTCGGCACAACGAACAAGGTAAATTCTCTATTTATGTCGGTACAAAAATGTAGACGAGTACATCCAGCTCG : 1358
BjuTT8B-d : TTCCCGTGCTTGATGGCGTTTTGGAACTCGGCACAACGAACAAGGTAAATTCTCTATTTATGTCGGTACAAAAATGTAAACGAGTACATCCAGCTCG : 1358
BjuTT8B-y : TTCCCGTGCTTGATGGCGTTTTGGAACTCGGCACAACGAACAAGGTAAATTCTCTATTTATGTCGGTACAAAAATGTAAACGAGTACATCCAGCTCG : 1358
            TTCCCGTGCTTGATGGCGTTTTGGAACTCGGCACAACGAACAAGGTAAATTCTCTATTTATGTCGGTACAAAAATGTA ACGAGTACATCCAGCTCG

                                                1400                                                  *
BniTT8B-b : TTCTATATTACTTCGACAAATAAAGAAAAATAATATACAGACACATCAACCATTTCGCAGTCATATGATAAAAAATATAGTTATTTCATACATTATA : 1455
BcaTT8B-b : TTCTATATTACTTCGACAAATAAAGAAAAATAATATACAGACACATCAACCATTTCGCAGTCATATGATAAAAAATATAGTTATTTCATACATTATA : 1455
BcaTT8B-y : TTCTATATTACTTCGACAAATAAAGAAAAATAATATACAGACACATCAACCATTTCGCAGTCATATGATAAAAAATATAGTTATTTCATACATTATA : 1455
BjuTT8B-d : TTCTATATTACTTCGACAAATAAAG-AAAATAATATACAGACACATCAACCATTTCGCAGTCATATGAT-AAAAAATATAGTTATTTCATACATTATA : 1453
BjuTT8B-y : TTCTATATTACTTCGACAAATAAAG-AAAATAATATACAGACACATCAACCATTTCGCAGTCATATGAT-AAAAAATATAGTTATTTCATACATTATA : 1453
            TTCTATATTACTTCGACAAATAAAG AAAATAATATACAGACACATCAACCATTTCGCAGTCATATGAT AAAAAATATAGTTATTTCATACATTATA

                                                    1500                         extron6                *
BniTT8B-b : TACACAAGCAAATGATGTCAAAATACACAACCTAATGAAAAATATGCGTGATATGTTGTGTTTATATGTGAAGGTCAAAGAAAGTGAAGAGTTTGTT : 1552
BcaTT8B-b : TACACAAGCAAATGATGTCAAAATACACAACCTAATGAAAAATATGCGTGATATGTTGTGTTTATATGTGAAGGTCAAAGAAAGTGAAGAGTTTGTT : 1552
BcaTT8B-y : TACACAAGCAAATGATGTCAAAATACACAACCTAATGAAAAATATGCGTGATATGTTGTGTTTATATGTGAAGGTCAAAGAAAGTGAAGAGTTTGTT : 1552
BjuTT8B-d : TACACAAGCAAATGATGTCAAAATACACAACCTAATGAAAAATATGCGTGATATGTTGTGTTTATATGTGAAGGTCAAAGAAAGTGAAGAGTTTGTT : 1550
BjuTT8B-y : TACACAAGCAAATGATGTCAAAATACACAACCTAATGAAAAATATGCGTGATATGTTGTGTTTATATGTGAAGGTCAAAGAAAGTGAAGAGTTTGTT : 1550
            TACACAAGCAAATGATGTCAAAATACACAACCTAATGAAAAATATGCGTGATATGTTGTGTTTATATGTGAAGGTCAAAGAAAGTGAAGAGTTTGTT

                                                        1600
BniTT8B-b : GAGCACATAAAGAGTTTCTTCCACAACCACCCGAAGTCAAACACTAAGCCTGCTCTTTTTGAACACTCAATCAACGAAGAGCATGAAGAAGTAGAAG : 1649
BcaTT8B-b : GAGCACATAAAGAGTTTCTTCCACAACCACCCGAAGTCAAACACTAAGCCTGCTCTTTTTGAACACTCAATCAACGAAGAGCATGAAGAAGTAGAAG : 1649
BcaTT8B-y : GAGCACATAAAGAGTTTCTTCCACAACCACCCGAAGTCAAACACTAAGCCTGCTCTTTTTGAACACTCAATCAACGAAGAGCATGAAGAAGTAGAAG : 1649
BjuTT8B-d : GAGCACATAAAGAGTTTCTTCCACAACCACCCGAAGTCAAACACTAAGCCTGCTCTTTTTGAACACTCAATCAACGAAGAGCATGAAGAAGTAGAAG : 1647
BjuTT8B-y : GAGCACATAAAGAGTTTCTTCCACAACCACCCGAAGTCAAACACTAAGCCTGCTCTTTTTGAACACTCAATCAACGAAGAGCATGAAGAAGTAGAAG : 1647
            GAGCACATAAAGAGTTTCTTCCACAACCACCCGAAGTCAAACACTAAGCCTGCTCTTTTTGAACACTCAATCAACGAAGAGCATGAAGAAGTAGAAG

            *                                                   1700
BniTT8B-b : AAGAAGAAATGACAATGTCAGAGGAGATAAGACTTGGTTCTCCTGATGACGATGACGTCTCCAATCAAAATCTAATCTCTGATTTTCATATAGAAGC : 1746
BcaTT8B-b : AAGAAGAAATGACAATGTCAGAGGAGATAAGACTTGGTTCTCCTGATGACGATGACGTCTCCAATCAAAATCTAATCTCTGATTTTCATATAGAAGC : 1746
BcaTT8B-y : AAGAAGAAATGACAATGTCAGAGGAGATAAGACTTGGTTCTCCTGATGACGATGACGTCTCCAATCAAAATCTAATCTCTGATTTTCATATAGAAGC : 1746
BjuTT8B-d : AAGAAGAAATGACAATGTCAGAGGAGATAAGACTTGGTTCTCCTGATGACGATGACGTCTCCAATCAAAATCTAATCTCTGATTTTCATATAGAAGC : 1744
BjuTT8B-y : AAGAAGAAATGACAATGTCAGAGGAGATAAGACTTGGTTCTCCTGATGACGATGACGTCTCCAATCAAAATCTAATCTCTGATTTTCATATAGAAGC : 1744
            AAGAAGAAATGACAATGTCAGAGGAGATAAGACTTGGTTCTCCTGATGACGATGACGTCTCCAATCAAAATCTAATCTCTGATTTTCATATAGAAGC

                *                                                   1800
BniTT8B-b : AACCAATAGTTTAGGTATACCGTACACCTTTCTTATTACGTTAAATTAGAATAACATTTTAATTAATTTTCTAATAATATATTTTTAAATCTGCTCT : 1843
BcaTT8B-b : AACCAATAGTTTAGGTATACCGTACACCTTTCTTATTACGTTAAATTAGAATAACATTTTAATTAATTTTCTAATAATATATTTTTAAATCTGCTCT : 1843
BcaTT8B-y : AACCAATAGTTTAGGTATACCGTACACCTTTCTTATTACGTTAAATTAGAATAACATTTTAATTAATTTTCTAATAATATATTTTTAAATCTGCTCT : 1843
BjuTT8B-d : AACCAATAGTTTAGGTATACCGTACACCTTTCTTATTACGTTAAATTAGAATAACATTTTAATTAATTTTCTAATAATATATTTTTAAATCTGCTCT : 1841
BjuTT8B-y : AACCAATAGTTTAGGTATACCGTACACCTTTCTTATTACGTTAAATTAGAATAACATTTTAATTAATTTTCTAATAATATATTTTTAAATCTGCTCT : 1841
            AACCAATAGTTTAGGTATACCGTACACCTTTCTTATTACGTTAAATTAGAATAACATTTTAATTAATTTTCTAATAATATATTTTTAAATCTGCTCT

                    *                                                   1900
BniTT8B-b : CTGATTTCCATATAGAAGCAACCAATAGTGTAGGTATACCGTGCACCTTTCCTGTTACATTAAATTAGTTAACAATATCATTATAATTAATTTCCTA : 1940
BcaTT8B-b : CTGATTTCCATATAGAAGCAACCAATAGTGTAGGTATACCGTGCACCTTTCCTGTTACATTAAATTAGTTAACAATATCATTATAATTAATTTCCTA : 1940
BcaTT8B-y : CTGATTTCCATATAGAAGCAACCAATAGTGTAGGTATACCGTGCACCTTTCCTGTTACATTAAATTAGTTAACAATATCATTATAATTAATTTCCTA : 1940
BjuTT8B-d : CTGATTTCCATATAGAAGCAACCAATAGTGTAGGTATACCGTGCACCTTTCCTGTTACATTAAATTAGTTAACAATATCATTATAATTAATTTCCTA : 1938
BjuTT8B-y : CTGATTTCCATATAGAAGCAACCAATAGTGTAGGTATACCGTGCACCTTTCCTGTTACATTAAATTAGTTAACAATATCATTATAATTAATTTCCTA : 1938
            CTGATTTCCATATAGAAGCAACCAATAGTGTAGGTATACCGTGCACCTTTCCTGTTACATTAAATTAGTTAACAATATCATTATAATTAATTTCCTA

                        *                                                 2000 extron7
BniTT8B-b : ACAATATAGATTTTTAAGTGTTTCTCATGTCTGGTAATTCTAACATCTATCATTGTATATATAGATACACATATGGACATGATGAATCTAATGGAGG : 2037
BcaTT8B-b : ACAATATAGATTTTTAAGTGTTTCTCATGTCTGGTAATTCTAACATCTATCATTGTATATATAGATACACATATGGACATGATGAATCTAATGGAGG : 2037
BcaTT8B-y : ACAATATAGATTTTTAAGTGTTTCTCATGTCTGGTAATTCTAACATCTATCATTGTATATATAGATACACATATGGACATGATGAATCTAATGGAGG : 2037
BjuTT8B-d : ACAATATAGATTTTTAAGTGTTTCTCATGTCTGGTAATTCTAACATCTATCATTGTATATATAGATACACATATGGACATGATGAATCTAATGGAGG : 2035
BjuTT8B-y : ACAATATAGATTTTTAAGTGTTTCTCATGTCTGGTAATTCTAACATCTATCATTGTATATATAGATACACATATGGACATGATGAATCTAATGGAGG : 2035
            ACAATATAGATTTTTAAGTGTTTCTCATGTCTGGTAATTCTAACATCTATCATTGTATATATAGATACACATATGGACATGATGAATCTAATGGAGG

                            *                                                   2100
BniTT8B-b : AAGGCGGAAAATATTCTCAGACAGTATCAACACTTCTCATGTCACAACCCACCAGTCTTCTTTCAGATTCAGTTTCCACATCTTCTTACGTTCAATC : 2134
BcaTT8B-b : AAGGCGGAAAATATTCTCAGACAGTATCAACACTTCTCATGTCACAACCCACCAGTCTTCTTTCAGATTCAGTTTCCACATCTTCTTACGTTCAATC : 2134
BcaTT8B-y : AAGGCGGAAAATATTCTCAGACAGTATCAACACTTCTCATGTCACAACCCACCAGTCTTCTTTCAGATTCAGTTTCCACATCTTCTTACGTTCAATC : 2134
BjuTT8B-d : AAGGCGGAAAATATTCTCAGACAGTATCAACACTTCTCATGTCACAACCCACCAGTCTTCTTTCAGATTCAGTTTCCACATCTTCTTACGTTCAATC : 2132
BjuTT8B-y : AAGGCGGAAAATATTCTCAGACAGTATCAACACTTCTCATGTCACAACCCACCAGTCTTCTTTCAGATTCAGTTTCCACATCTTCTTACGTTCAATC : 2132
            AAGGCGGAAAATATTCTCAGACAGTATCAACACTTCTCATGTCACAACCCACCAGTCTTCTTTCAGATTCAGTTTCCACATCTTCTTACGTTCAATC
```

```
                                *                                              2200
BniTT8B-b : ATCGTTTGTCTCGTGGAGAGTTGAGAATGTCAAAGAGCATCAGCAATATCAGCGAGTGGAGAAAGCGTCGTCGTCGTCGTCTCAATGGATGCTCAAA : 2231
BcaTT8B-b : ATCGTTTGTCTCGTGGAGAGTTGAGAATGTCAAAGAGCATCAGCAATATCAGCGAGTGGAGAAAGCGTCGTCGTCGTCGTCTCAATGGATGCTCAAA : 2231
BcaTT8B-y : ATCGTTTGTCTCGTGGAGAGTTGAGAATGTCAAAGAGCATCAGCAATATCAGCGAGTGGAGAAAGCGTCGTCGTCGTCGTCTCAATGGATGCTCAAA : 2231
BjuTT8B-d : ATCGTTTGTCTCGTGGAGAGTTGAGAATGTCAAAGAGCATCAGCAATATCAGCGAGTGGAGAAAGCGTCGTCGTCGTCGTCTCAATGGATGCTCAAA : 2229
BjuTT8B-y : ATCGTTTGTCTCGTGGAGAGTTGAGAATGTCAAAGAGCATCAGCAATATCAGCGAGTGGAGAAAGCGTCGTCGTCGTCGTCTCAATGGATGCTCAAA : 2229
            ATCGTTTGTCTCGTGGAGAGTTGAGAATGTCAAAGAGCATCAGCAATATCAGCGAGTGGAGAAAGCGTCGTCGTCGTCGTCTCAATGGATGCTCAAA

                                   *                                              2300
BniTT8B-b : CACATAATCTTGAGAGTTCCTTTCCTCCACGACAACACTAAAAATAAGAAGCTACCGCGGGAAGAGCTTAACCATGTGGTGGCTGAGCGACGCAGAA : 2328
BcaTT8B-b : CACATAATCTTGAGAGTTCCTTTCCTCCACGACAACACTAAAAATAAGAAGCTACCGCGGGAAGAGCTTAACCATGTGGTGGCTGAGCGACGCAGAA : 2328
BcaTT8B-y : CACATAATCTTGAGAGTTCCTTTCCTCCACGACAACACTAAAAATAAGAAGCTACCGCGGGAAGAGCTTAACCATGTGGTGGCTGAGCGACGCAGAA : 2328
BjuTT8B-d : CACATAATCTTGAGAGTTCCTTTCCTCCACGACAACACTAAAAATAAGAAGCTACCGCGGGAAGAGCTTAACCATGTGGTGGCTGAGCGACGCAGAA : 2326
BjuTT8B-y : CACATAATCTTGAGAGTTCCTTTCCTCCACGACAACACTAAAAATAAGAAGCTACCGCGGGAAGAGCTTAACCATGTGGTGGCTGAGCGACGCAGAA : 2326
            CACATAATCTTGAGAGTTCCTTTCCTCCACGACAACACTAAAAATAAGAAGCTACCGCGGGAAGAGCTTAACCATGTGGTGGCTGAGCGACGCAGAA

                                      *                                              2400
BniTT8B-b : GAGAGAAGCTAAACGAGAGATTCATAACGTTGAGATCATTGGTTCCATTTGTGACCAAGATGGATAAAGTCTCAATCCTTGGAGACACCATTCAGTA : 2425
BcaTT8B-b : GAGAGAAGCTAAACGAGAGATTCATAACGTTGAGATCATTGGTTCCATTTGTGACCAAGATGGATAAAGTCTCAATCCTTGGAGACACCATTCAGTA : 2425
BcaTT8B-y : GAGAGAAGCTAAACGAGAGATTCATAACGTTGAGATCATTGGTTCCATTTGTGACCAAGATGGATAAAGTCTCAATCCTTGGAGACACCATTCAGTA : 2425
BjuTT8B-d : GAGAGAAGCTAAACGAGAGATTCATAACGTTGAGATCATTGGTTCCATTTGTGACCAAGATGGATAAAGTCTCAATCCTTGGAGACACCATTCAGTA : 2423
BjuTT8B-y : GAGAGAAGCTAAACGAGAGATTCATAACGTTGAGATCATTGGTTCCATTTGTGACCAAGATGGATAAAGTCTCAATCCTTGGAGACACCATTCAGTA : 2423
            GAGAGAAGCTAAACGAGAGATTCATAACGTTGAGATCATTGGTTCCATTTGTGACCAAGATGGATAAAGTCTCAATCCTTGGAGACACCATTCAGTA

                                         *                                              2500
BniTT8B-b : CGTAAACCATCTTTGTAAGAGGATCCATGAGCTAGAATCTACTCATCACGAGCCAGCACATAAGCGGATGCGTATCGGTAAGGGAAGAACTTGGGAA : 2522
BcaTT8B-b : CGTAAACCATCTTTGTAAGAGGATCCATGAGCTAGAATCTACTCATCACGAGCCAGCACATAAGCGGATGCGTATCGGTAAGGGAAGAACTTGGGAA : 2522
BcaTT8B-y : CGTAAACCATCTTTGTAAGAGGATCCATGAGCTAGAATCTACTCATCACGAGCCAGCACATAAGCGGATGCGTATCGGTAAGGGAAGAACTTGGGAA : 2522
BjuTT8B-d : CGTAAACCATCTTTGTAAGAGGATCCATGAGCTAGAATCTACTCATCACGAGCCAGCACATAAGCGGATGCGTATCGGTAAGGGAAGAACTTGGGAA : 2520
BjuTT8B-y : CGTAAACCATCTTTGTAAGAGGATCCATGAGCTAGAATCTACTCATCACGAGCCAGCACATAAGCGGATGCGTATCGGTAAGGGAAGAACTTGGGAA : 2520
            CGTAAACCATCTTTGTAAGAGGATCCATGAGCTAGAATCTACTCATCACGAGCCAGCACATAAGCGGATGCGTATCGGTAAGGGAAGAACTTGGGAA

                                            *                                              2600
BniTT8B-b : GAGGTGGAGGTTTCCATTATAGAGAGCGATGTTTTATTAGAGATGAGATGCGTGTACCGAGATGGTTTATTGCTCGACATTCTTCAGGTACTTAAGG : 2619
BcaTT8B-b : GAGGTGGAGGTTTCCATTATAGAGAGCGATGTTTTATTAGAGATGAGATGCGTGTACCGAGATGGTTTATTGCTCGACATTCTTCAGGTACTTAAGG : 2619
BcaTT8B-y : GAGGTGGAGGTTTCCATTATAGAGAGCGATGTTTTATTAGAGATGAGATGCGTGTACCGAGATGGTTTATTGCTCGACATTCTTCAGGTACTTAAGG : 2619
BjuTT8B-d : GAGGTGGAGGTTTCCATTATAGAGAGCGATGTTTTATTAGAGATGAGATGCGTGTACCGAGATGGTTTATTGCTCGACATTCTTCAGGTACTTAAGG : 2617
BjuTT8B-y : GAGGTGGAGGTTTCCATTATAGAGAGCGATGTTTTATTAGAGATGAGATGCGTGTACCGAGATGGTTTATTGCTCGACATTCTTCAGGTACTTAAGG : 2617
            GAGGTGGAGGTTTCCATTATAGAGAGCGATGTTTTATTAGAGATGAGATGCGTGTACCGAGATGGTTTATTGCTCGACATTCTTCAGGTACTTAAGG

                                               *                                              2700
BniTT8B-b : TGCTCGGTATAGAGACCACTGCGGTTCACACCGTCGTGAACGACCATGATTTTGAGGCAGAGATAAGGGCGAAGGTGAGAGGGAAGAAACCAACCAT : 2716
BcaTT8B-b : TGCTCGGTATAGAGACCACTGCGGTTCACACCGTCGTGAACGACCATGATTTTGAGGCAGAGATAAGGGCGAAGGTGAGAGGGAAGAAACCAACCAT : 2716
BcaTT8B-y : TGCTCGGTATAGAGACCACTGCGGTTCACACCGTCGTGAACGACCATGATTTTGAGGCAGAGATAAGGGCGAAGGTGAGAGGGAAGAAACCAACCAT : 2716
BjuTT8B-d : TGCTGGGTATAGAGACCACTGCGGTTCACACCGTCGTGAACGACCATGATTTTGAGGCAGAGATAAGGGCGAAGGTGAGAGGGAAGAAACCAACCAT : 2714
BjuTT8B-y : TGCTGGGTATAGAGACCACTGCGGTTCACACCGTCGTGAACGACCATGATTTTGAGGCAGAGATAAGGGCGAAGGTGAGAGGGAAGAAACCAACCAT : 2714
            TGCT GGTATAGAGACCACTGCGGTTCACACCGTCGTGAACGACCATGATTTTGAGGCAGAGATAAGGGCGAAGGTGAGAGGGAAGAAACCAACCAT

                                                  *
BniTT8B-b : TGCTGAGGTTAAAATAGCCATCCATCAAATCATATCTCAAAATAAACTCTAG : 2768
BcaTT8B-b : TGCTGAGGTTAAAATAGCCATCCATCAAATCATATCTCAAAATAAACTCTAG : 2768
BcaTT8B-y : TGCTGAGGTTAAAATAGCCATCCATCAAATCATATCTCAAAATAAACTCTAG : 2768
BjuTT8B-d : TGCTGAGGTTAAAATAGCCATCCATCAAATCATATCTCAAAATAAACTCTAG : 2766
BjuTT8B-y : TGCTGAGGTTAAAATAGCCATCCATCAAATCATATCTCAAAATAAACTCTAG : 2766
            TGCTGAGGTTAAAATAGCCATCCATCAAATCATATCTCAAAATAAACTCTAG
```
