# Supplementary material for: Metabolite Profiling and Transcriptome Analysis Provide Insight into Seed Coat Color in Brassica juncea
Source: Int J Mol Sci. 2021 Jul 5;22(13):7215. doi: 10.3390/ijms22137215 (PMC8268557; doi:10.3390/ijms22137215)
Supplement: Supplementary file 1 [file ijms-22-07215-s001.zip › ijms-1245090-SI/Supplementary Figure S7.pdf]

```
              extron1
                                                                     *
BolTT8C-b : ATGGATGAATTAAGTATTATACCGTTATGGAAAGTGATCGGGGCTGAGAAAAAAGAGATTCAAGGGCTACTTAAGGCGGTGGTGCAATCTGTGGGGT :  97
BolTT8C-y : ATGGATGAATTAAGTATTATACCGTTATGGAAAGTGATCGGGGCTGAGAAAGAAGAGATTCAAGGGCTACTTAAGGCGGTGGTGCAATCTGTGGGGT :  97
BnaTT8C-b : ATGGATGAATTAAGTATTATACCGTTATGGAAAGTGATCGGGGCTGAGAAAGAAGAGATTCAAGGGCTACTTAAGGCGGTGGTGCAATCTGTGGGGT :  97
BnaTT8C-y : ATGGATGAATTAAGTATTATACCGTTATGGAAAGTGATCGGGGCTGAGAAAGAAGAGATTCAAGGGCTACTTAAGGCGGTGGTGCAATCTGTGGGGT :  97
BcaTT8C-b : ATGGATGAATTAAGTATTATACCGTTATGGAAAGTGATCGGGGCTGAGAAAAAAGAGATTCAAGGGCTACTTAAGGCGGTGGTGCAATCTGTGGGGT :  97
BcaTT8C-y : ATGGATGAATTAAGTATTATACCGTTATGGAAAGTGATCGGGGCTGAGAAAGAAGAGATTCAAGGGCTACTTAAGGCGGTGGTGCAATCTGTGGGGT :  97
            ATGGATGAATTAAGTATTATACCGTTATGGAAAGTGATCGGGGCTGAGAAA AAGAGATTCAAGGGCTACTTAAGGCGGTGGTGCAATCTGTGGGGT

            100                                                      *
BolTT8C-b : GGACTTATAGTGTCTTCTGGCAACTTTGTCCTCAACGAAGGTTCTCTTTTCATTTCATCCATCTCTCACAATATATAAAGCAATATATTTATCTTTA :  194
BolTT8C-y : GGACTTATAGTGTCTTCTGGCAACTTTGTCCTCAACGAAGGTTCTCTTTTCATTTCATCCATCTCTCACAATATATAAAGCAATATATTTATCTTTA :  194
BnaTT8C-b : GGACTTATAGTGTCTTCTGGCAACTTTGTCCTCAACGAAGGTTCTCTTTTCATTTCATCCATCTCTCACAATATATAAAGCAATATATTTATCTTTA :  194
BnaTT8C-y : GGACTTATAGTGTCTTCTGGCAACTTTGTCCTCAACGAAGGTTCTCTTTTCATTTCATCCATCTCTCACAATATATAAAGCAATATATTTATCTTTA :  194
BcaTT8C-b : GGACTTATAGTGTCTTCTGGCAACTTTGTCCTCAACGAAGGTTCTCTTTTCATTTCATCCATCTCTCACAATATATAAAGCAATATATTTATCTTTA :  194
BcaTT8C-y : GGACTTATAGTGTCTTCTGGCAACTTTGTCCTCAACGAAGGTTCTCTTTTCATTTCATCCATCTCTCACAATATATAAAGCAATATATTTATCTTTA :  194
            GGACTTATAGTGTCTTCTGGCAACTTTGTCCTCAACGAAGGTTCTCTTTTCATTTCATCCATCTCTCACAATATATAAAGCAATATATTTATCTTTA

               200                                   extron2       *
BolTT8C-b : TTAATTATAATAAGTAGAAGTTACTAAATGTTAAATCGATTAGGAAATTGTTGTGGAGTAGTGGAAACTATAACGGTGCAATAAAGACTAGAAAGAC :  291
BolTT8C-y : TTAATTATAATAAGTAGAAGTTACTAAATGTTAAATCGATTAGGAAATTGTTGTGGAGTAGTGGAAACTATAACGGTGCAATAAAGACTAGAAAGAC :  291
BnaTT8C-b : TTAATTATAATAAGTAGAAGTTACTAAATGTTAAATCGATTAGGAAATTGTTGTGGAGTAGTGGAAACTATAACGGTGCAATAAAGACTAGAAAGAC :  291
BnaTT8C-y : TTAATTATAATAAGTAGAAGTTACTAAATGTTAAATCGATTAGGAAATTGTTGTGGAGTAGTGGAAACTATAACGGTGCAATAAAGACTAGAAAGAC :  291
BcaTT8C-b : TTAATTATAATAAGTAGAAGTTACTAAATGTTAAATCGATTAGGAAATTGTTGTGGAGTAGTGGAAACTATAACGGTGCAATAAAGACTAGAAAGAC :  291
BcaTT8C-y : TTAATTATAATAAGTAGAAGTTACTAAATGTTAAATCGATTAGGAAATTGTTGTGGAGTAGTGGAAACTATAACGGTGCAATAAAGACTAGAAAGAC :  291
            TTAATTATAATAAGTAGAAGTTACTAAATGTTAAATCGATTAGGAAATTGTTGTGGAGTAGTGGAAACTATAACGGTGCAATAAAGACTAGAAAGAC

                  300                                                      *
BolTT8C-b : AACTCAGCCGGCGGAAGTTACGGCTGAAGAGGCTGCGTCGGAAAGAAGCCAACAGCTCATGGAGCTTTACGAGACGCTTTTTGCTGGAGAATCATCG :  388
BolTT8C-y : AACTCAGCCGGCGGAAGTTACGGCTGAAGAGGCTGCGTCGGAAAGAAGCCAACAGCTCATGGAGCTTTACGAGACGCTTTTTGCTGGAGAATCATCG :  388
BnaTT8C-b : AACTCAGCCGGCGGAAGTTACGGCTGAAGAGGCTGCGTCGGAAAGAAGCCAACAGCTCATGGAGCTTTACGAGACGCTTTTTGCTGGAGAATCATCG :  388
BnaTT8C-y : AACTCAGCCGGCGGAAGTTACGGCTGAAGAGGCTGCGTCGGAAAGAAGCCAACAGCTCATGGAGCTTTACGAGACGCTTTTTGCTGGAGAATCATCG :  388
BcaTT8C-b : AACTCAGCCGGCGGAAGTTACGGCTGAAGAGGCTGCGTCGGAAAGAAGCCAACAGCTCATGGAGCTTTACGAGACGCTTTTTGCTGGAGAATCATCG :  388
BcaTT8C-y : AACTCAGCCGGCGGAAGTTACGGCTGAAGAGGCTGCGTCGGAAAGAAGCCAACAGCTCATGGAGCTTTACGAGACGCTTTTTGCTGGAGAATCATCG :  388
            AACTCAGCCGGCGGAAGTTACGGCTGAAGAGGCTGCGTCGGAAAGAAGCCAACAGCTCATGGAGCTTTACGAGACGCTTTTTGCTGGAGAATCATCG

                     400                                                      *
BolTT8C-b : ATGGAAGCGAGGGCTTGCACAGCACTGTCGCCGGAGGATTTGACGGATCCTGAATGGTTTTATGTGCTGTGTTTCACTTACTCTTTCGAACCTCCTT :  485
BolTT8C-y : ATGGAAGCGAGGGCTTGCACAGCACTGTCGCCGGAGGATTTGACGGATCCTGAATGGTTTTATGTGCTGTGTTTCACTTACTCTTTCGAACCTCCTT :  485
BnaTT8C-b : ATGGAAGCGAGGGCTTGCACAGCACTGTCGCCGGAGGATTTGACGGATCCTGAATGGTTTTATGTGCTGTGTTTCACTTACTCTTTCGAACCTCCTT :  485
BnaTT8C-y : ATGGAAGCGAGGGCTTGCACAGCACTGTCGCCGGAGGATTTGACGGATCCTGAATGGTTTTATGTGCTGTGTTTCACTTACTCTTTCGAACCTCCTT :  485
BcaTT8C-b : ATGGAAGCGAGGGCTTGCACAGCACTGTCGCCGGAGGATTTGACGGATCCTGAATGGTTTTATGTGCTGTGTTTCACTTACTCTTTCGAACCTCCTT :  485
BcaTT8C-y : ATGGAAGCGAGGGCTTGCACAGCACTGTCGCCGGAGGATTTGACGGATCCTGAATGGTTTTATGTGCTGTGTTTCACTTACTCTTTCGAACCTCCTT :  485
            ATGGAAGCGAGGGCTTGCACAGCACTGTCGCCGGAGGATTTGACGGATCCTGAATGGTTTTATGTGCTGTGTTTCACTTACTCTTTCGAACCTCCTT

                        500                                                      *
BolTT8C-b : CTGGGTACAACAGCTCTCTCTCTGTCTTCAAAGTTTTTTTTTCTTTTCAAAAAGACTCTACCCAGAGTTTCTTAATTTGTCCTTTTCATCTTCTCTT :  582
BolTT8C-y : CTGGGTACAACAGCTCTCTCTCTGTCTTCAAAGTTTTTTTTTCTTTTCAAAAAGACTCTACCCAGAGTTTCTTAATTTGTCCTTTTCATCTTCTCTT :  582
BnaTT8C-b : CTGGGTACAACAGCTCTCTCTCTGTCTTCAAAGTTTTTTTTTCTTTTCAAAAAGACTCTACCCAGAGTTTCTTAATTTGTCCTTTTCATCTTCTCTT :  582
BnaTT8C-y : CTGGGTACAACAGCTCTCTCTCTGTCTTCAAAGTTTTTTTTTCTTTTCAAAAAGACTCTACCCAGAGTTTCTTAATTTGTCCTTTTCATCTTCTCTT :  582
BcaTT8C-b : CTGGGTACAACAGCTCTCTCTCTGTCTTCAAAGTTTTTTTTTCTTTTCAAAAAGACTCTACCCAGAGTTTCTTAATTTGTCCTTTTCATCTTCTCTT :  582
BcaTT8C-y : CTGGGTACAACAGCTCTCTCTCTGTCTTCAAAGTTTTTTTTTCTTTTCAAAAAGACTCTACCCAGAGTTTCTTAATTTGTCCTTTTCATCTTCTCTT :  582
            CTGGGTACAACAGCTCTCTCTCTGTCTTCAAAGTTTTTTTTTCTTTTCAAAAAGACTCTACCCAGAGTTTCTTAATTTGTCCTTTTCATCTTCTCTT

                           600                                                      *
BolTT8C-b : AGAGAAGACAAAAATAGTATTGTGTGTTAAATGTGAATCACAAATACTACGGAAGCATTAAAGACAAACTGGGGACTTTAAGTTACTGAAAGTAGAA :  679
BolTT8C-y : AGAGAAGACAAAAATAGTATTGTGTGTTAAATGCGAATCACAAATACTATGGAAGCATTAAAGACAAACTGGGGACTTTAAGTTACTGAAAGTAGAA :  679
BnaTT8C-b : AGAGAAGACAAAAATAGTATTGTGTGTTAAATGTGAATCACAAATACTACGGAAGCATTAAAGACAAACTGGGGACTTTAAGTTACTGAAAGTAGAA :  679
BnaTT8C-y : AGAGAAGACAAAAATAGTATTGTGTGTTAAATGTGAATCACAAATACTACGGAAGCATTAAAGACAAACTGGGGACTTTAAGTTACTGAAAGTAGAA :  679
BcaTT8C-b : AGAGAAGACAAAAATAGTATTGTGTGTTAAATGTGAATCACAAATACTACGGAAGCATTAAAGACAAACTGGGGACTTTAAGTTACTGAAAGTAGAA :  679
BcaTT8C-y : AGAGAAGACAAAAATAGTATTGTGTGTTAAATGCGAATCACAAATACTATGGAAGCATTAAAGACAAACTGGGGACTTTAAGTTACTGAAAGTAGAA :  679
            AGAGAAGACAAAAATAGTATTGTGTGTTAAATG GAATCACAAATACTA GGAAGCATTAAAGACAAACTGGGGACTTTAAGTTACTGAAAGTAGAA

                              700                                                      *
BolTT8C-b : ATGTATTGAATTTAGTGAAAACGTACACTTCATTTTGGTGAACATAATTGGACCGTTGAGATTCTTATTGGTTTGTTTATTGATTATCTAAAGTAGG :  776
BolTT8C-y : ATGTATTGAATTTAGTGAAAACGTACACTTCATTTTGGTGAACATAATTGGACCGTTGAGATTCTTATTGGTTTGTTTATTGAGTATCTAAAGTAGG :  776
BnaTT8C-b : ATGTATTGAATTTAGTGAAAACGTACACTTCATTTTGGTGAACATAATTGGACCGTTGAGATTCTTATTGGTTTGTTTATTGATTATCTAAAGTAGG :  776
BnaTT8C-y : ATGTATTGAATTTAGTGAAAACGTACACTTCATTTTGGTGAACATAATTGGACCGTTGAGATTCTTATTGGTTTGTTTATTGATTATCTAAAGTAGG :  776
BcaTT8C-b : ATGTATTGAATTTAGTGAAAACGTACACTTCATTTTGGTGAACATAATTGGACCGTTGAGATTCTTATTGGTTTGTTTATTGATTATCTAAAGTAGG :  776
BcaTT8C-y : ATGTATTGAATTTAGTGAAAACGTACACTTCATTTTGGTGAACATAATTGGACCGTTGAGATTCTTATTGGTTTGTTTATTGAGTATCTAAAGTAGG :  776
            ATGTATTGAATTTAGTGAAAACGTACACTTCATTTTGGTGAACATAATTGGACCGTTGAGATTCTTATTGGTTTGTTTATTGA TATCTAAAGTAGG

                                 800                                                      *
BolTT8C-b : AGCATATATAGATGATAAATGCATAACAAAGTGTGGTAGTTATCGGTATAATTAATGTTTTTTCTCTATGGAGGAAAAAATCAAAATATAATGCGGA :  873
BolTT8C-y : AGCATATATAGATGATAAATGCATAACAAAGTGTGTTAGTTATCGGTATAATTAATGTTTTTTCTCTATGGAGGAAAAAATCAAAATATAATGCGGA :  873
BnaTT8C-b : AGCATATATAGATGATAAATGCATAACAAAGTGTGGTAGTTATCGGTATAATTAATGTTTTTTCTCTATGGAGGAAAAAATCAAAATATAATGCGGA :  873
BnaTT8C-y : AGCATATATAGATGATAAATGCATAACAAAGTGTGGTAGTTATCGGTATAATTAATGTTTTTTCTCTATGGAGGAAAAAATCAAAATATAATGCGGA :  873
BcaTT8C-b : AGCATATATAGATGATAAATGCATAACAAAGTGTGGTAGTTATCGGTATAATTAATGTTTTTTCTCTATGGAGGAAAAAATCAAAATATAATGCGGA :  873
BcaTT8C-y : AGCATATATAGATGATAAATGCATAACAAAGTGTGTTAGTTATCGGTATAATTAATGTTTTTTCTCTATGGAGGAAAAAATCAAAATATAATGCGGA :  873
            AGCATATATAGATGATAAATGCATAACAAAGTGTG TAGTTATCGGTATAATTAATGTTTTTTCTCTATGGAGGAAAAAATCAAAATATAATGCGGA

                          extron3900                                                      *
BolTT8C-b : AGTATTAATTTGTAGGATGCCAGGAAAGGCGTATGCGAGGAGGAAGCACATATGGCTAAGTGGTGCAAATGAGGTTGACAATAAAATCTTCTCTAGG :  970
BolTT8C-y : AGTATTAATTTGTAGGATGCCAGGAAAGGCGTATGCGAGGAGGAAGCACATATGGCTAAGTGGTGCAAATGAGGTTGACAATAAAATCTTCTCTAGG :  970
BnaTT8C-b : AGTATTAATTTGTAGGATGCCAGGAAAGGCGTATGCGAGGAGGAAGCACATATGGCTAAGTGGTGCAAATGAGGTTGACAATAAAATCTTCTCTAGG :  970
BnaTT8C-y : AGTATTAATTTGTAGGATGCCAGGAAAGGCGTATGCGAGGAGGAAGCACATATGGCTAAGTGGTGCAAATGAGGTTGACAATAAAATCTTCTCTAGG :  970
BcaTT8C-b : AGTATTAATTTGTAGGATGCCAGGAAAGGCGTATGCGAGGAGGAAGCACATATGGCTAAGTGGTGCAAATGAGGTTGACAATAAAATCTTCTCTAGG :  970
BcaTT8C-y : AGTATTAATTTGTAGGATGCCAGGAAAGGCGTATGCGAGGAGGAAGCACATATGGCTAAGTGGTGCAAATGAGGTTGACAATAAAATCTTCTCTAGG :  970
            AGTATTAATTTGTAGGATGCCAGGAAAGGCGTATGCGAGGAGGAAGCACATATGGCTAAGTGGTGCAAATGAGGTTGACAATAAAATCTTCTCTAGG
```

```
                                       1000                                                                          *
BolTT8C-b : GCTATTTCTGCAAAGGTTTATTTCCTTTTATTCATTCACCACTACACTGTGCATCTATTTCTACTTATTTAGATATACGCAATTTTATATATATCTC : 1067
BolTT8C-y : GCTATTTCTGCAAAGGTTTATTTCCTTTTATTCATTCACCACTACACTGTGCATCTATTTCTACTTATTTAGATATACGCAATTTTATATATATCTC : 1067
BnaTT8C-b : GCTATTTCTGCAAAGGTTTATTTCCTTTTATTCATTCACCACTACACTGTGCATCTATTTCTACTTATTTAGATATACGCAATTTTATATATATCTC : 1067
BnaTT8C-y : GCTATTTCTGCAAAGGTTTATTTCCTTTTATTCATTCACCACTACACTGTGCATCTATTTCTACTTATTTAGATATACGCAATTTTATATATATCTC : 1067
BcaTT8C-b : GCTATTTCTGCAAAGGTTTATTTCCTTTTATTCATTCACCACTACACTGTGCATCTATTTCTACTTATTTAGATATACGCAATTTTATATATATCTC : 1067
BcaTT8C-y : GCTATTTCTGCAAAGGTTTATTTCCTTTTATTCATTCACCACTACACTGTGCATCTATTTCTACTTATTTAGATATACGCAATTTTATATATATCTC : 1067
            GCTATTTCTGCAAAGGTTTATTTCCTTTTATTCATTCACCACTACACTGTGCATCTATTTCTACTTATTTAGATATACGCAATTTTATATATATCTC

                                          1100                                      extron4                 *
BolTT8C-b : ATTCTTCAAACTAATTAATTTTATCTTCGTCTTTACTTGAATGCTCGCTCTTTCCACATTAGAGTGCCAAAATTCAGGTAAATTTCGCCGTCATTAA : 1164
BolTT8C-y : ATTCTTCAAACTAATTAATTTTATCTTCGTCTTTACTTGAATGCTCGCTCTTTCCACATTAGAGTGCCAAAATTCAGGTAAATTTCGCCGTCATTAA : 1164
BnaTT8C-b : ATTCTTCAAACTAATTAATTTTATCTTCGTCTTTACTTGAATGCTCGCTCTTTCCACATTAGAGTGCCAAAATTAAGGTAAATTTCGCCGTCATTAA : 1164
BnaTT8C-y : ATTCTTCAAACTAATTAATTTTATCTTCGTCTTTACTTGAATGCTCGCTCTTTCCACATTAGAGTGCCAAAATTCAGGTAAATTTCGCCGTCATTAA : 1164
BcaTT8C-b : ATTCTTCAAACTAATTAATTTTATCTTCGTCTTTACTTGAATGCTCGCTCTTTCCACATTAGAGTGCCAAAATTCAGGTAAATTTCGCCGTCATTAA : 1164
BcaTT8C-y : ATTCTTCAAACTAATTAATTTTATCTTCGTCTTTACTTGAATGCTCGCTCTTTCCACATTAGAGTGCCAAAATTCAGGTAAATTTCGCCGTCATTAA : 1164
            ATTCTTCAAACTAATTAATTTTATCTTCGTCTTTACTTGAATGCTCGCTCTTTCCACATTAGAGTGCCAAAATTcAGGTAAATTTCGCCGTCATTAA

                                             1200                                                              *
BolTT8C-b : TTAATTATTTAATTTTCGTAGAAACGAAGGTATCAATTAATAAAAGTTATTTTACATGTTGACAAAAAAGAAGAAGTTATTTTACATATTTGAGAAT : 1261
BolTT8C-y : TTAATTATTTAATTTTCGTAGAAACGAAGGTATCAATTAATAAAAGTTATTTTACATGTTGACAAAAAAGAAAAAGTTATTTTACATATTTGAAAAT : 1261
BnaTT8C-b : TTAATTATTTAATTTTCGTAGAAACGAAGGTATCAATTAATAAAAGTTATTTTACATGTTGACAAAAAAGAAGAAGTTATTTTACATATTTGAGAAT : 1261
BnaTT8C-y : TTAATTATTTAATTTTCGTAGAAACGAAGGTATCAATTAATAAAAGTTATTTTACATGTTGACAAAAAAGAAGAAGTTATTTTACATATTTGAGAAT : 1261
BcaTT8C-b : TTAATTATTTAATTTTCGTAGAAACGAAGGTATCAATTAATAAAAGTTATTTTACATGTTGACAAAAAAGAAGAAGTTATTTTACATATTTGAGAAT : 1261
BcaTT8C-y : TTAATTATTTAATTTTCGTAGAAACGAAGGTATCAATTAATAAAAGTTATTTTACATGTTGACAAAAAAGAAAAAGTTATTTTACATATTTGAAAAT : 1261
            TTAATTATTTAATTTTCGTAGAAACGAAGGTATCAATTAATAAAAGTTATTTTACATGTTGACAAAAAAGAA AAGTTATTTTACATATTTGA AAT

                                          1300 extron5                                                          *
BolTT8C-b : TTTTTTGTGATGAAAAAAAAAAACAAAAATAAAAACAGACAGTGGTTTGCATTCCCGTGCTTGATGGCGTTTTGGAACTAGGCACAACGAACAAGGTA : 1358
BolTT8C-y : TTTTTTGTGATGAAAAAAAAAAACAAATAAAAAAACAGACAGTGGTTTGCATTCCCGTGCTTGATGGCGTTTTGGAACTAGGCACAACGAACAAGGTA : 1358
BnaTT8C-b : TTTTTTGTGATGAAAAAAAAAAACAAATAAAAAAACAGACAGTGGTTTGCATTCCCGTGCTTGATGGCGTTTTGGAACTAGGCACAACGAACAAGGTA : 1358
BnaTT8C-y : TTTTTTGTGATGAAAAAAAAAAACAAATAAAAAAACAGACAGTGGTTTGCATTCCCGTGCTTGATGGCGTTTTGGAACTAGGCACAACGAACAAGGTA : 1358
BcaTT8C-b : TTTTTTGTGATGAAAAAAAAAAACAAAAATAAAAACAGACAGTGGTTTGCATTCCCGTGCTTGATGGCGTTTTGGAACTAGGCACAACGAACAAGGTA : 1358
BcaTT8C-y : TTTTTTGTGATGAAAAAAAAAAACAAATAAAAAAACAGACAGTGGTTTGCATTCCCGTGCTTGATGGCGTTTTGGAACTAGGCACAACGAACAAGGTA : 1358
            TTTTTTGTGATGAAAAAAAAAAACAAA A AAAAACAGACAGTGGTTTGCATTCCCGTGCTTGATGGCGTTTTGGAACTAGGCACAACGAACAAGGTA

                                                1400                                                                *
BolTT8C-b : AAAATCTCTATTTATGTCGGTACCCAAAATGTAGACGAATATAGTCAGCTCATTCTATGTTACTTAGAGAAATAAAGAAAAATAATATACAGAAACA : 1455
BolTT8C-y : AAAATCTCTATTTATGTCGGTACCCAAAATGTAGACGAATATAGTCAGCTCATTCTATGTTACTTAGAGAAATAAAGAAAAATAATATACAGAAACA : 1455
BnaTT8C-b : AAAATCTCTATTTATGTCGGTACCCAAAATGTAGACGAATATAGTCAGCTCATTCTATGTTACTTAGAGAAATAAAGAAAAATAATATACAGAAACA : 1455
BnaTT8C-y : AAAATCTCTATTTATGTCGGTACCCAAAATGTAGACGAATATAGTCAGCTCATTCTATGTTACTTAGAGAAATAAAGAAAAATAATATACAGAAACA : 1455
BcaTT8C-b : AAAATCTCTATTTATGTCGGTACCCAAAATGTAGACGAATATAGTCAGCTCATTCTATGTTACTTAGAGAAATAAAGAAAAATAATATACAGAAACA : 1455
BcaTT8C-y : AAAATCTCTATTTATGTCGGTACCCAAAATGTAGACGAATATAGTCAGCTCATTCTATGTTACTTAGAGAAATAAAGAAAAATAATATACAGAAACA : 1455
            AAAATCTCTATTTATGTCGGTACCCAAAATGTAGACGAATATAGTCAGCTCATTCTATGTTACTTAGAGAAATAAAGAAAAATAATATACAGAAACA

                                                       1500                                                          *
BolTT8C-b : TCAACCATTCGCAGTCATATGATAAAAAATATAGTTTTTCGATTACATTATATACACAACCAAATGATGTAAAATATACAACCTAATGAAAATATGC : 1552
BolTT8C-y : TCAACCATTCGCAGTCATATGATAAAAAATATAGTTTTTCGATTACATTATATACACAACCAAATGATGTAAAATATACAACCTAATGAAAATATGC : 1552
BnaTT8C-b : TCAACCATTCGCAGTCATATGATAAAAAATATAGTTTTTCGATTACATTATATACACAACCAAATGATGTAAAATATACAACCTAATGAAAATATGC : 1552
BnaTT8C-y : TCAACCATTCGCAGTCATATGATAAAAAATATAGTTTTTCGATTACATTATATACACAACCAAATGATGTAAAATATACAACCTAATGAAAATATGC : 1552
BcaTT8C-b : TCAACCATTCGCAGTCATATGATAAAAAATATAGTTTTTCGATTACATTATATACACAACCAAATGATGTAAAATATACAACCTAATGAAAATATGC : 1552
BcaTT8C-y : TCAACCATTCGCAGTCATATGATAAAAAATATAGTTTTTCGATTACATTATATACACAACCAAATGATGTAAAATATACAACCTAATGAAAATATGC : 1552
            TCAACCATTCGCAGTCATATGATAAAAAATATAGTTTTTCGATTACATTATATACACAACCAAATGATGTAAAATATACAACCTAATGAAAATATGC

                                                            1600    extron6
BolTT8C-b : ATAAGAGGAAGAACCAGGGAGATGGATGCATGATATATTGTGTTTGTATGTGAAGGTCAAAGAGAGTGAAGAGTTTGTTGACCACATAAAGAGTTTC : 1649
BolTT8C-y : ATAAGAGGAAGAACCAGGGAGATGGATGCATGATATATTGTGTTTGTATGTGAAGGTCAAAGAGAGTGAAGAGTTTGTTGACCACATAAAGAGTTTC : 1649
BnaTT8C-b : ATAAGAGGAAGAACCAGGGAGATGGATGCATGATATATTGTGTTTGTATGTGAAGGTCAAAGAGAGTGAAGAGTTTGTTGACCACATAAAGAGTTTC : 1649
BnaTT8C-y : ATAAGAGGAAGAACCAGGGAGATGGATGCATGATATATTGTGTTTGTATGTGAAGGTCAAAGAGAGTGAAGAGTTTGTTGACCACATAAAGAGTTTC : 1649
BcaTT8C-b : ATAAGAGGAAGAACCAGGGAGATGGATGCATGATATATTGTGTTTGTATGTGAAGGTCAAAGAGAGTGAAGAGTTTGTTGACCACATAAAGAGTTTC : 1649
BcaTT8C-y : ATAAGAGGAAGAACCAGGGAGATGGATGCATGATATATTGTGTTTGTATGTGAAGGTCAAAGAGAGTGAAGAGTTTGTTGACCACATAAAGAGTTTC : 1649
            ATAAGAGGAAGAACCAGGGAGATGGATGCATGATATATTGTGTTTGTATGTGAAGGTCAAAGAGAGTGAAGAGTTTGTTGACCACATAAAGAGTTTC

            *                                    1700
BolTT8C-b : TTCCACAACTACCCGAAGTCAAACACTAAGCCTACTCTTTCTGAACACTTCATCAACGAAGAGCGTGAAGAAGACGAAGACGAAGTAGAAGAAGAAG : 1746
BolTT8C-y : TTCCACAACTACCCGAAGTCAAACACTAAGCCTACTCTTTCTGAACACTTCATCAACGAAGAGCGTGAAGAAGACGAAGACGAAGTAGAAGAAGAAG : 1746
BnaTT8C-b : TTCCACAACTACCCGAAGTCAAACACTAAGCCTACTCTTTCTGAACACTTCATCAACGAAGAGCGTGAAGAAGACGAAGACGAAGTAGAAGAAGAAG : 1746
BnaTT8C-y : TTCCACAACTACCCGAAGTCAAACACTAAGCCTACTCTTTCTGAACACTTCATCAACGAAGAGCGTGAAGAAGACGAAGACGAAGTAGAAGAAGAAG : 1746
BcaTT8C-b : TTCCACAACTACCCGAAGTCAAACACTAAGCCTACTCTTTCTGAACACTTCATCAACGAAGAGCGTGAAGAAGACGAAGACGAAGTAGAAGAAGAAG : 1746
BcaTT8C-y : TTCCACAACTACCCGAAGTCAAACACTAAGCCTACTCTTTCTGAACACTTCATCAACGAAGAGCGTGAAGAAGACGAAGACGAAGTAGAAGAAGAAG : 1746
            TTCCACAACTACCCGAAGTCAAACACTAAGCCTACTCTTTCTGAACACTTCATCAACGAAGAGCGTGAAGAAGACGAAGACGAAGTAGAAGAAGAAG

               *                                        1800
BolTT8C-b : AAATGACAATGTCAGAGGAGATAAGACTTGGTTCTCCTGATGACGATGACGTCTCCAATCAAAATCTACTCTCTGATTTCCATATAGAAGCAACCAA : 1843
BolTT8C-y : AAATGACAATGTCAGAGGAGATAAGACTTGGTTCTCCTGATGACGATGACGTCTCCAATCAAAATCTACTCTCTGATTTCCATATAGAAGCAACCAA : 1843
BnaTT8C-b : AAATGACAATGTCAGAGGAGATAAGACTTGGTTCTCCTGATGACGATGACGTCTCCAATCAAAATCTACTCTCTGATTTCCATATAGAAGCAACCAA : 1843
BnaTT8C-y : AAATGACAATGTCAGAGGAGATAAGACTTGGTTCTCCTGATGACGATGACGTCTCCAATCAAAATCTACTCTCTGATTTCCATATAGAAGCAACCAA : 1843
BcaTT8C-b : AAATGACAATGTCAGAGGAGATAAGACTTGGTTCTCCTGATGACGATGACGTCTCCAATCAAAATCTACTCTCTGATTTCCATATAGAAGCAACCAA : 1843
BcaTT8C-y : AAATGACAATGTCAGAGGAGATAAGACTTGGTTCTCCTGATGACGATGACGTCTCCAATCAAAATCTACTCTCTGATTTCCATATAGAAGCAACCAA : 1843
            AAATGACAATGTCAGAGGAGATAAGACTTGGTTCTCCTGATGACGATGACGTCTCCAATCAAAATCTACTCTCTGATTTCCATATAGAAGCAACCAA

                  *                                          1900
BolTT8C-b : TAGTTTAGGTATACCGTACACACCTTTCTTATTACATTAAATTAGTTAACAATATCATTATAATAAATTTTCTAATAATAAATTATTTAAACTGGTT : 1940
BolTT8C-y : TAGTTTAGGTATACCGTACACACCTTTCTTATTACATTAAATTAGTTAACAATATCATTATAATAAATTTTCTAATAATAAATTATTTAAACTGGTT : 1940
BnaTT8C-b : TAGTTTAGGTATACCGTACACACCTTTCTTATTACATTAAATTAGTTAACAATATCATTATAATAAATTTTCTAATAATAAATTATTTAAACTGGTT : 1940
BnaTT8C-y : TAGTTTAGGTATACCGTACACACCTTTCTTATTACATTAAATTAGTTAACAATATCATTATAATAAATTTTCTAATAATAAATTATTTAAACTGGTT : 1940
BcaTT8C-b : TAGTTTAGGTATACCGTACACACCTTTCTTATTACATTAAATTAGTTAACAATATCATTATAATAAATTTTCTAATAATAAATTATTTAAACTGGTT : 1940
BcaTT8C-y : TAGTTTAGGTATACCGTACACACCTTTCTTATTACATTAAATTAGTTAACAATATCATTATAATAAATTTTCTAATAATAAATTATTTAAACTGGTT : 1940
            TAGTTTAGGTATACCGTACACACCTTTCTTATTACATTAAATTAGTTAACAATATCATTATAATAAATTTTCTAATAATAAATTATTTAAACTGGTT
```

extron7

```
                    *                    2000
BolTT8C-b : CTCATGTCTGGTAATTCTAACATCTATCATTGTATATATAGATACACACATGGACATGATGAATCTAATGGAGGAAGGCGGAAATTATTCTCAGACA : 2037
BolTT8C-y : CTCATGTCTGGTAATTCTAACATCTATCATTGTATATATAGATACACACATGGACATGATGAATCTAATGGAGGAAGGCGGAAATTATTCTCAGACA : 2037
BnaTT8C-b : CTCATGTCTGGTAATTCTAACATCTATCATTGTATATATAGATACACACATGGACATGATGAATCTAATGGAGGAAGGCGGAAATTATTCTCAGACA : 2037
BnaTT8C-y : CTCATGTCTGGTAATTCTAACATCTATCATTGTATATATAGATACACACATGGACATGATGAATCTAATGGAGGAAGGCGGAAATTATTCTCAGACA : 2037
BcaTT8C-b : CTCATGTCTGGTAATTCTAACATCTATCATTGTATATATAGATACACACATGGACATGATGAATCTAATGGAGGAAGGCGGAAATTATTCTCAGACA : 2037
BcaTT8C-y : CTCATGTCTGGTAATTCTAACATCTATCATTGTATATATAGATACACACATGGACATGATGAATCTAATGGAGGAAGGCGGAAATTATTCTCAGACA : 2037
            CTCATGTCTGGTAATTCTAACATCTATCATTGTATATATAGATACACACATGGACATGATGAATCTAATGGAGGAAGGCGGAAATTATTCTCAGACA

                        *                                       2100
BolTT8C-b : GTATCAACACTTCTCATGTCACAACCCACCAGTCTTCTTTCAGATTCAGTTTCCACATCTTCTTACGTTCAATCATCGTTTATATCGTGGAGAGTTG : 2134
BolTT8C-y : GTATCAACACTTCTCATGTCACAACCCACCAGTCTTCTTTCAGATTCAGTTTCCACATCTTCTTACGTTCAATCATCGTTTATATCGTGGAGAGTTG : 2134
BnaTT8C-b : GTATCAACACTTCTCATGTCACAACCCACCAGTCTTCTTTCAGATTCAGTTTCCACATCTTCTTACGTTCAATCATCGTTTATATCGTGGAGAGTTG : 2134
BnaTT8C-y : GTATCAACACTTCTCATGTCACAACCCACCAGTCTTCTTTCAGATTCAGTTTCCACATCTTCTTACGTTCAATCATCGTTTATATCGTGGAGAGTTG : 2134
BcaTT8C-b : GTATCAACACTTCTCATGTCACAACCCACCAGTCTTCTTTCAGATTCAGTTTCCACATCTTCTTACGTTCAATCATCGTTTATATCGTGGAGAGTTG : 2134
BcaTT8C-y : GTATCAACACTTCTCATGTCACAACCCACCAGTCTTCTTTCAGATTCAGTTTCCACATCTTCTTACGTTCAATCATCGTTTATATCGTGGAGAGTTG : 2134
            GTATCAACACTTCTCATGTCACAACCCACCAGTCTTCTTTCAGATTCAGTTTCCACATCTTCTTACGTTCAATCATCGTTTATATCGTGGAGAGTTG

                            *                                       2200
BolTT8C-b : AGAATGTCAAAGAGCATCAGCAATATCAGCGAGTGGAAAAAGCGGCGTCTTCGTCGTCGCAATGGATGCTCAAACACATAATCTTGAAAGTTCCTTT : 2231
BolTT8C-y : AGAATGTCAAAGAGCATCAGCAATATCAGCGAGTGGAAAAAGCGGCGTCTTCGTCGTCGCAATGGATGCTCAAACACATAATCTTGAAAGTTCCTTT : 2231
BnaTT8C-b : AGAATGTCAAAGAGCATCAGCAATATCAGCGAGTGGAAAAAGCGGCGTCTTCGTCGTCGCAATGGATGCTCAAACACATAATCTTGAAAGTTCCTTT : 2231
BnaTT8C-y : AGAATGTCAAAGAGCATCAGCAATATCAGCGAGTGGAAAAAGCGGCGTCTTCGTCGTCGCAATGGATGCTCAAACACATAATCTTGAAAGTTCCTTT : 2231
BcaTT8C-b : AGAATGTCAAAGAGCATCAGCAATATCAGCGAGTGGAAAAAGCGGCGTCTTCGTCGTCGCAATGGATGCTCAAACACATAATCTTGAAAGTTCCTTT : 2231
BcaTT8C-y : AGAATGTCAAAGAGCATCAGCAATATCAGCGAGTGGAAAAAGCGGCGTCTTCGTCGTCGCAATGGATGCTCAAACACATAATCTTGAAAGTTCCTTT : 2231
            AGAATGTCAAAGAGCATCAGCAATATCAGCGAGTGGAAAAAGCGGCGTCTTCGTCGTCGCAATGGATGCTCAAACACATAATCTTGAAAGTTCCTTT

                                *                                       2300
BolTT8C-b : CCTCCACGACAACACTAAAAATAAGAGGCTGCCGCGAGAAGAGCTTAACCATGTGGTGGCCGAGCGACGCAGAAGAGAGAAGCTAAATGAGAGATTC : 2328
BolTT8C-y : CCTCCACGACAACACTAAAAATAAGAGGCTGCCGCGAGAAGAGCTTAACCATGTGGTGGCCGAGCGACGCAGAAGAGAGAAGCTAAATGAGAGATTC : 2328
BnaTT8C-b : CCTCCACGACAACACTAAAAATAAGAGGCTGCCGCGAGAAGAGCTTAACCATGTGGTGGCCGAGCGACGCAGAAGAGAGAAGCTAAATGAGAGATTC : 2328
BnaTT8C-y : CCTCCACGACAACACTAAAAATAAGAGGCTGCCGCGAGAAGAGCTTAACCATGTGGTGGCCGAGCGACGCAGAAGAGAGAAGCTAAATGAGAGATTC : 2328
BcaTT8C-b : CCTCCACGACAACACTAAAAATAAGAGGCTGCCGCGAGAAGAGCTTAACCATGTGGTGGCCGAGCGACGCAGAAGAGAGAAGCTAAATGAGAGATTC : 2328
BcaTT8C-y : CCTCCACGACAACACTAAAAATAAGAGGCTGCCGCGAGAAGAGCTTAACCATGTGGTGGCCGAGCGACGCAGAAGAGAGAAGCTAAATGAGAGATTC : 2328
            CCTCCACGACAACACTAAAAATAAGAGGCTGCCGCGAGAAGAGCTTAACCATGTGGTGGCCGAGCGACGCAGAAGAGAGAAGCTAAATGAGAGATTC

                                    *                                       2400
BolTT8C-b : ATAACGTTGAGATCATTGGTTCCATTTGTGACCAAGATGGATAAAGTCTCGATCCTTGGAGACACCATTGAGTACGTAAACCATCTTTCTAAGAGGA : 2425
BolTT8C-y : ATAACGTTGAGATCATTGGTTCCATTTGTGACCAAGATGGATAAAGTCTCGATCCTTGGAGACACCATTGAGTACGTAAACCATCTTTCTAAGAGGA : 2425
BnaTT8C-b : ATAACGTTGAGATCATTGGTTCCATTTGTGACCAAGATGGATAAAGTCTCGATCCTTGGAGACACCATTGAGTACGTAAACCATCTTTCTAAGAGGA : 2425
BnaTT8C-y : ATAACGTTGAGATCATTGGTTCCATTTGTGACCAAGATGGATAAAGTCTCGATCCTTGGAGACACCATTGAGTACGTAAACCATCTTTCTAAGAGGA : 2425
BcaTT8C-b : ATAACGTTGAGATCATTGGTTCCATTTGTGACCAAGATGGATAAAGTCTCGATCCTTGGAGACACCATTGAGTACGTAAACCATCTTTCTAAGAGGA : 2425
BcaTT8C-y : ATAACGTTGAGATCATTGGTTCCATTTGTGACCAAGATGGATAAAGTCTCGATCCTTGGAGACACCATTGAGTACGTAAACCATCTTTCTAAGAGGA : 2425
            ATAACGTTGAGATCATTGGTTCCATTTGTGACCAAGATGGATAAAGTCTCGATCCTTGGAGACACCATTGAGTACGTAAACCATCTTTCTAAGAGGA

                                        *                                       2500
BolTT8C-b : TCCATGAGCTGGAATCTACTCATCACGAGCCAAACCAAAAGCGGATGCGTATCGGTAAGGGAAGAACTTGGGAAGAGGTGGAGGTTTCCATTATAGA : 2522
BolTT8C-y : TCCATGAGCTGGAATCTACTCATCACGAGCCAAACCAAAAGCGGATGCGTATCGGTAAGGGAAGAACTTGGGAAGAGGTGGAGGTTTCCATTATAGA : 2522
BnaTT8C-b : TCCATGAGCTGGAATCTACTCATCACGAGCCAAACCAAAAGCGGATGCGTATCGGTAAGGGAAGAACTTGGGAAGAGGTGGAGGTTTCCATTATAGA : 2522
BnaTT8C-y : TCCATGAGCTGGAATCTACTCATCACGAGCCAAACCAAAAGCGGATGCGTATCGGTAAGGGAAGAACTTGGGAAGAGGTGGAGGTTTCCATTATAGA : 2522
BcaTT8C-b : TCCATGAGCTGGAATCTACTCATCACGAGCCAAACCAAAAGCGGATGCGTATCGGTAAGGGAAGAACTTGGGAAGAGGTGGAGGTTTCCATTATAGA : 2522
BcaTT8C-y : TCCATGAGCTGGAATCTACTCATCACGAGCCAAACCAAAAGCGGATGCGTATCGGTAAGGGAAGAACTTGGGAAGAGGTGGAGGTTTCCATTATAGA : 2522
            TCCATGAGCTGGAATCTACTCATCACGAGCCAAACCAAAAGCGGATGCGTATCGGTAAGGGAAGAACTTGGGAAGAGGTGGAGGTTTCCATTATAGA

                                            *                                       2600
BolTT8C-b : GAGCGATGTTTTGTTAGAGATGAGATGCGAGTACCGAGATGGTTTATTGCTCAACATTCTTCAGGTACTTAAGGAGCTAGGTATAGAGACCACTGCG : 2619
BolTT8C-y : GAGCGATGTTTTGTTAGAGATGAGATGCGAGTACCGAGATGGTTTATTGCTCAACATTCTTCAGGTACTTAAGGAGCTAGGTATAGAGACCACTGCG : 2619
BnaTT8C-b : GAGCGATGTTTTGTTAGAGATGAGATGCGAGTACCGAGATGGTTTATTGCTCAACATTCTTCAGGTACTTAAGGAGCTAGGTATAGAGACCACTGCG : 2619
BnaTT8C-y : GAGCGATGTTTTGTTAGAGATGAGATGCGAGTACCGAGATGGTTTATTGCTCAACATTCTTCAGGTACTTAAGGAGCTAGGTATAGAGACCACTGCG : 2619
BcaTT8C-b : GAGCGATGTTTTGTTAGAGATGAGATGCGAGTACCGAGATGGTTTATTGCTCAACATTCTTCAGGTACTTAAGGAGCTAGGTATAGAGACCACTGCG : 2619
BcaTT8C-y : GAGCGATGTTTTGTTAGAGATGAGATGCGAGTACCGAGATGGTTTATTGCTCAACATTCTTCAGGTACTTAAGGAGCTAGGTATAGAGACCACTGCG : 2619
            GAGCGATGTTTTGTTAGAGATGAGATGCGAGTACCGAGATGGTTTATTGCTCAACATTCTTCAGGTACTTAAGGAGCTAGGTATAGAGACCACTGCG

                                                *                                       2700
BolTT8C-b : GTTCACACCGCCTTGAACGACCACCATTTTGAGGCAGAGATAAGGGCGAAAGTGAGAGGGAAGAAACCAACCATTGCTGAGGTTAAAATAGCCATCC : 2716
BolTT8C-y : GTTCACACCGCCTTGAACGACCACCATTTTGAGGCAGAGATAAGGGCGAAAGTGAGAGGGAAGAAACCAACCATTGCTGAGGTTAAAATAGCCATCC : 2716
BnaTT8C-b : GTTCACACCGCCTTGAACGACCACCATTTTGAGGCAGAGATAAGGGCGAAAGTGAGAGGGAAGAAACCAACCATTGCTGAGGTTAAAATAGCCATCC : 2716
BnaTT8C-y : GTTCACACCGCCTTGAACGACCACCATTTTGAGGCAGAGATAAGGGCGAAAGTGAGAGGGAAGAAACCAACCATTGCTGAGGTTAAAATAGCCATCC : 2716
BcaTT8C-b : GTTCACACCGCCTTGAACGACCACCATTTTGAGGCAGAGATAAGGGCGAAAGTGAGAGGGAAGAAACCAACCATTGCTGAGGTTAAAATAGCCATCC : 2716
BcaTT8C-y : GTTCACACCGCCTTGAACGACCACCATTTTGAGGCAGAGATAAGGGCGAAAGTGAGAGGGAAGAAACCAACCATTGCTGAGGTTAAAATAGCCATCC : 2716
            GTTCACACCGCCTTGAACGACCACCATTTTGAGGCAGAGATAAGGGCGAAAGTGAGAGGGAAGAAACCAACCATTGCTGAGGTTAAAATAGCCATCC

BolTT8C-b : ATCAAATCATATATAATAATAAACTCTAG : 2745
BolTT8C-y : ATCAAATCATATATAATAATAAACTCTAG : 2745
BnaTT8C-b : ATCAAATCATATATAATAATAAACTCTAG : 2745
BnaTT8C-y : ATCAAATCATATATAATAATAAACTCTAG : 2745
BcaTT8C-b : ATCAAATCATATATAATAATAAACTCTAG : 2745
BcaTT8C-y : ATCAAATCATATATAATAATAAACTCTAG : 2745
            ATCAAATCATATATAATAATAAACTCTAG
```
